# A crystalline radical cation derived from Thiele’s hydrocarbon with redox range beyond 1 V

**DOI:** 10.1038/s41467-021-27104-y

**Published:** 2021-12-03

**Authors:** Ying Kai Loh, Petra Vasko, Caitilín McManus, Andreas Heilmann, William K. Myers, Simon Aldridge

**Affiliations:** 1 Inorganic Chemistry Laboratory, Department of Chemistry, University of Oxford, South Parks Road, Oxford, OX1 3QR UK; 2 Department of Chemistry, Nanoscience Center, University of Jyväskylä, P. O. Box 35, Jyväskylä, FI-40014 Finland

**Keywords:** Electronic materials, Inorganic chemistry

## Abstract

Thiele’s hydrocarbon occupies a central role as an open-shell platform for new organic materials, however little is known about its redox behaviour. While recent synthetic approaches involving symmetrical carbene substitution of the CPh_2_ termini yield isolable neutral/dicationic analogues, the intervening radical cations are much more difficult to isolate, due to narrow compatible redox ranges (typically < 0.25 V). Here we show that a hybrid BN/carbene approach allows access to an unsymmetrical analogue of Thiele’s hydrocarbon **1**, and that this strategy confers markedly enhanced stability on the radical cation. **1**^•**+**^ is stable across an exceptionally wide redox range (> 1 V), permitting its isolation in crystalline form. Further single-electron oxidation affords borenium dication **1**^**2+**^, thereby establishing an organoboron redox system fully characterized in all three redox states. We perceive that this strategy can be extended to other transient organic radicals to widen their redox stability window and facilitate their isolation.

## Abstract (web summary)

Organic molecules that can access various redox states have potential applications in electronics, batteries, catalysis, among others. Here the authors report the preparation of an unsymmetrical organoboron analogue of Thiele’s hydrocarbon and study its one- and two-electron oxidation reactions; remarkably, the radical cation is stable over a redox range of > 1 V and can also be isolated.

## Introduction

**Fig. 1:**
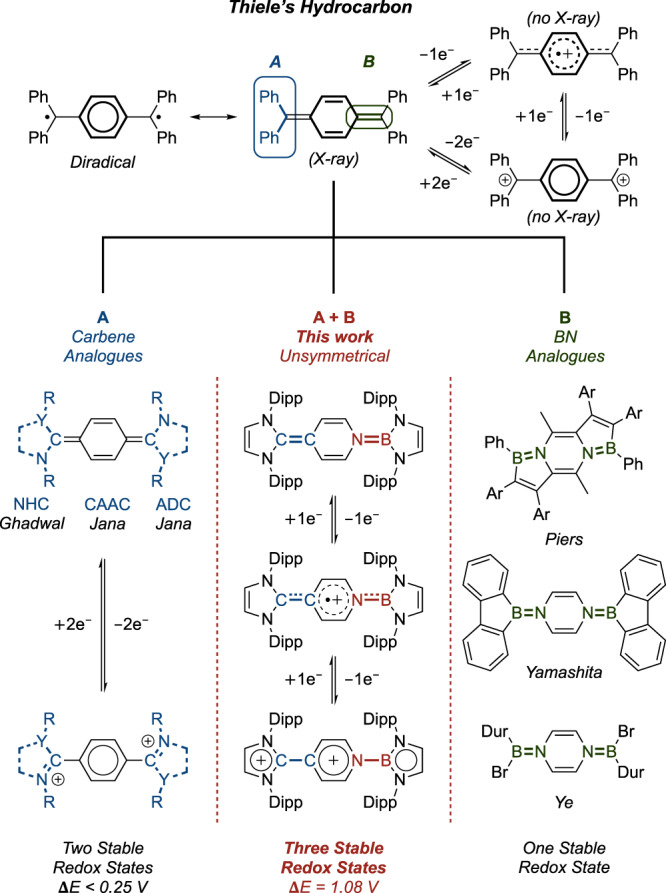
Systems derived from Thiele’s hydrocarbon. Thiele’s hydrocarbon, its radical cation and dication; previous modification methods **A**, **B** and present work. (Dipp = 2,6-diisopropylphenyl, NHC = N-heterocyclic carbene, CAAC = cyclic (alkyl)aminocarbene, ADC = acyclic diaminocarbene).

Shortly after Gomberg’s pioneering discovery of the persistent triphenylmethyl radical in 1900^1^, Thiele reported the first stable organic diradicaloid (‘Thiele’s hydrocarbon’; Fig. 1)^2^, derived from *p*-quinodimethane. Thiele’s hydrocarbon, which can attain aromaticity in its central ring, can be described by both closed-shell quinoidal (Kekulé) and open-shell diradical (non-Kekulé) resonance forms, accounting for its inherently high reactivity. Subsequently, its X-ray structure was determined, confirming its diradicaloid character^3^. Since then, Thiele’s hydrocarbon has been widely exploited and integrated into the design of new organic materials with small HOMO – LUMO energy gaps^4^.

Organic molecules that can reversibly access three stable redox states have received considerable recent attention^5–14^, reflecting applications as super-electron donors^15^, photo-/electro-catalysts^16^, redox switches^17^, or in redox-flow batteries etc^18^. However, such systems are rarely isolated, due to the redox instability of the intervening radical species^13^. Thiele’s hydrocarbon, which can gain aromaticity upon successive one-electron oxidations, presents itself as a privileged platform for such applications. However, its redox properties are far less studied, restricted by the transient nature of its radical monocation (*t*_1/2_ = 3 s), and structural information for both its mono- and dication are unknown (Fig. 1)^19^.

Carbenes have recently emerged as highly efficient ligands to stabilize organic radicals^20–27^, and have granted access to simple non-annulated versions of Thiele’s hydrocarbon (Method **A**, Fig. 1)^28–31^. This strategy contrasts with traditional (synthetically challenging) approaches which necessitate the use of multiple planar-fused π-conjugated rings to facilitate spin delocalization^4^. Importantly, the diverse library of available carbenes also allows for modular tuning of the CPh_2_ termini^32^. That said, recently reported analogues based on N-heterocyclic carbenes (NHCs) by Ghadwal et al., cyclic (alkyl)(amino)carbenes (CAACs) and acyclic diaminocarbenes (ADCs) by Jana et al. are characterized either by two single-electron transfer events at potentials which are very close (Δ*E* < 0.25 V)^28,29^, or by a single two-electron redox wave^30,31^, alluding to the instability of the respective mixed-valence radical cations towards disproportionation^13,33^. As such, these systems are typically limited to only two stable redox states, *viz.* the neutral and dicationic forms. Most recently, Jana et al. pushed this strategy further, reporting that symmetrical CH(NDippMe) disubstitution accesses a more stable radical cation with a redox range extended to 0.48 V^34^.

Main group heteroatoms have been introduced to modify the electronic properties of π-conjugated organic molecules^35,36^, leading to the synthesis of silicon^37^, mono-/dianionic boron^38,39^, and dicationic nitrogen analogues of Thiele’s hydrocarbon^40^. In regards to anionic boron analogues, three-state redox systems have been reported very recently, but the redox ranges associated with the intermediate radicals are also very narrow (Δ*E* = 0.20 V), thus limiting the stability of these radicals.

An attractive strategy to incorporate boron while still maintaining charge neutrality is to exploit the isoelectronic B=N/C=C relationship^41^. Interestingly, this approach has recently been shown to enhance the electronic coupling in electron-rich olefins, leading to isolable olefin radical cations, albeit at the expense of decreasing the stability of the dicationic species, meaning that a three-state redox system could not be achieved^42^. In the context of systems based on Thiele’s hydrocarbon, replacement of *both* exocyclic C=C double bonds with B=N successfully delivers isolable BN analogues, as reported by the groups of Piers, Yamashita and Ye (Method **B**, Fig. 1)^43–46^. However, in stark contrast to their all-carbon counterparts, these bis-BN analogues are only stable in their *neutral* states. Cyclic voltammetry reveals irreversible one-electron oxidations, hinting at the generation of extremely reactive radical cations and dications derived from these systems that contain a pair of highly electron-deficient boron atoms.

Inspired by these reports, we hypothesized that an unsymmetrical mono-BN analogue of Thiele’s hydrocarbon featuring isoelectronic NHC and N-heterocyclic boryl (NHB)^47–54^ substituents might allow access to an isolable radical cation with a significantly enhanced redox range, within an overall three-state system (Method **A** + **B**, Fig. 1). Importantly, B=N substitution (Method **B**) would be expected to render the intervening radical cation stable across a wide redox range by disfavouring the second oxidation step (*cf.* bis-BN systems), albeit moderated somewhat by the presence of the opposing NHC function (Method **A**) which would mitigate the enhanced electrophilicity at boron in the doubly oxidized dication^55^.

## Results and discussion

### Syntheses of neutral organoboron analogues of Thiele’s hydrocarbon

**Fig. 2:**
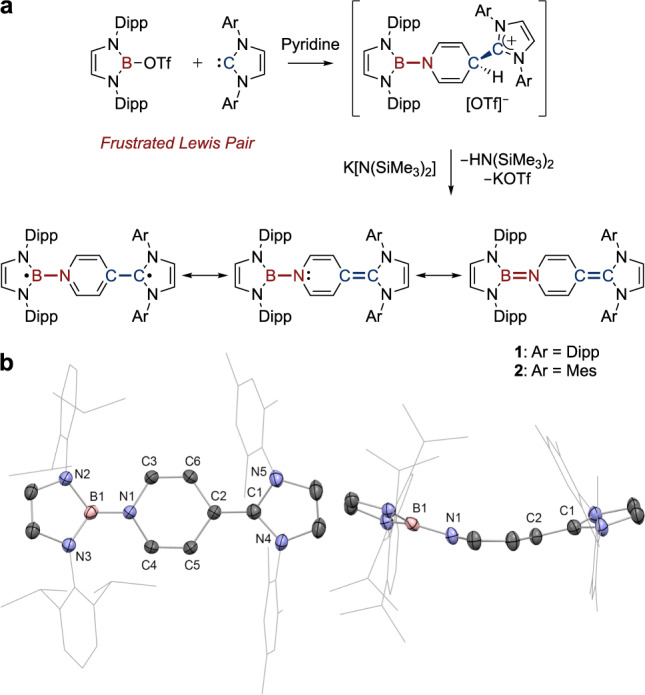
Syntheses and characterization of neutral organoboron analogues of Thiele’s hydrocarbon. **a** Syntheses of **1** and **2**. **b** Solid-state structure of **2** with side view. For clarity, H atoms are omitted, and Dipp/Mes groups are simplified as wireframes. Thermal ellipsoids set at 50% probability. Key distances (Å): B1−N1 1.424(4), C1−C2 1.356(4), N1−C3 1.415(4), N1−C4 1.413(3), C3−C6 1.325(4), C4−C5 1.334(4), C2−C6 1.460(4), C2−C5 1.447(4). (Mes = 2,4,6-trimethylphenyl).

The large steric profile inherent in both triflate-substituted borane (HCDippN)_2_BOTf and IDipp leads to the formation of a B/C frustrated Lewis pair (FLP) system (Fig. 2a)^56^. Upon exposure to pyridine at room temperature, a red solution was formed instantly, implying the facile nature of the 1,4-activation of pyridine. Subsequent deprotonation affords **1** as an orange-red solid; the ^1^H NMR spectrum displays two sets of heterocycle signals, consistent with the unsymmetrical environment of opposing NHB and NHC moieties. The boron atom of the NHB unit displays a broad signal at 19.9 ppm in the ^11^B{^1^H} NMR spectrum. However, single crystals of **1** gave weak diffraction patterns, and an X-ray structure could not be obtained. As a surrogate for **1**, we synthesized IMes-containing **2** via the same procedure. **2** gives rise to a similar ^11^B NMR signal (at *δ*_B_ = 19.0 ppm), and in this case crystallographic study unambiguously confirms the identity of **2** (and provides credence to **1**) as the target unsymmetrical organoboron analogue of Thiele’s hydrocarbon.

Structurally, **2** adopts a curved shape (Fig. 2b). The exocyclic B−N bond (1.424(4) Å) is shorter than the endocyclic B−N bonds (mean: 1.445(4) Å), consistent with a degree of double bond character, and the exocyclic C–C bond (1.356(4) Å) is slightly shorter than those of Thiele’s hydrocarbon and related bis-carbene derivatives (1.365(4) − 1.381(3) Å)^3,28–31^. DFT analyses of **1** and **2** show no significant geometric deviations between them and confirm the closed-shell nature of their ground states implied spectroscopically, in line with symmetrical bis-carbene and bis-BN analogues^28–31,43–46^. In the case of **1**, the calculated singlet-triplet gap is calculated to be 1.76 eV (169.4 kJ mol^−1^). Moreover, consideration of the natural bond orders obtained from NRT (natural resonance theory) calculations, suggests that the predominant resonance structure features exocyclic B–N single and C=C double bonds (Fig. 2a and Supporting Information Table S4). The localization of a lone pair at nitrogen implied by this resonance structure is consistent with the curved structure of **2**^12^.

**Fig. 3:**
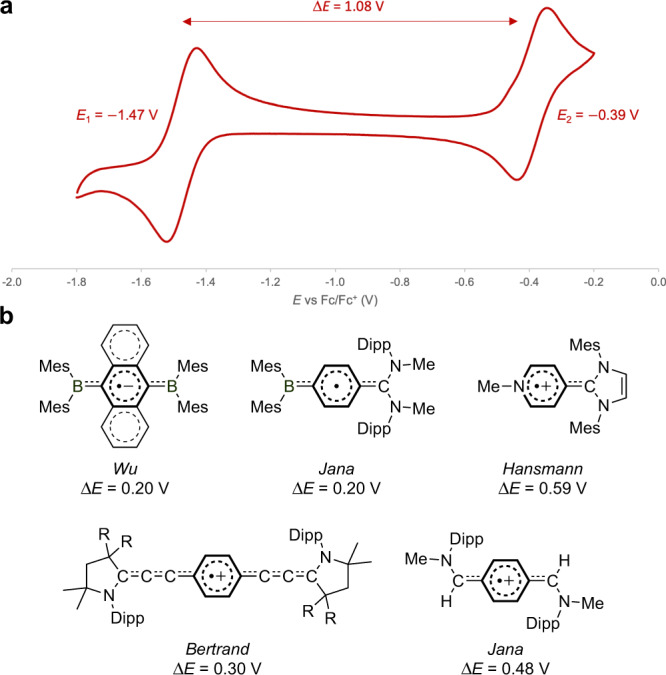
Electrochemical studies. **a** Cyclic voltammogram of **1** ([^*n*^Bu_4_N][PF_6_] in CH_2_Cl_2_ at 100 mV s^−1^). **b** Redox ranges associated with related structurally characterized radicals (Hansmann et al. reported in ref. ^12^ that a related radical cation [(MeCMesN)_2_C(C_5_H_4_N)Trip]^•**+**^ has redox range Δ*E* = 0.72 V, but this species has not been structurally characterized).

Cyclic voltammetry was used to elucidate the redox properties of **1**, revealing two reversible single-electron oxidations, consistent with the targeted three-state redox nature (Fig. 3a). The first oxidation wave (*E*_1_ = −1.47 V) falls within the range of bis-carbene systems (−1.42 to −0.95 V; Fig. 1)^28–31^, suggesting that **1** shares similarly strong reducing properties to its all-carbon congeners. Strikingly, the second oxidation wave (*E*_2_ = −0.39 V) occurs at a much more positive potential, leading to a markedly wider separation (Δ*E* = 1.08 V) between the two redox events.

The redox range associated with radical cation **1**^•**+**^ not only greatly exceeds that of the elusive radical cations of bis-carbene analogues (Δ*E* < 0.25 V; Fig. 1)^28–31^ but also other related structurally characterized radicals (Δ*E* = 0.20 − 0.59 V; Fig. 3b)^10,12,34,38,39^, hinting at a pronounced degree of radical stability conferred by BN-substitution. Notably, **1**^•**+**^ can also be viewed as a hybrid of the boryl-C_6_H_4_-carbene radical (Δ*E* = 0.20 V)^39^ reported by Jana et al. and the pyridyl-carbene radical cation (Δ*E* = 0.59 V)^12^ reported by Hansmann et al. — both of which exhibit much narrower redox ranges independently.

### Synthesis of a radical cationic organoboron analogue of Thiele’s hydrocarbon

**Fig. 4:**
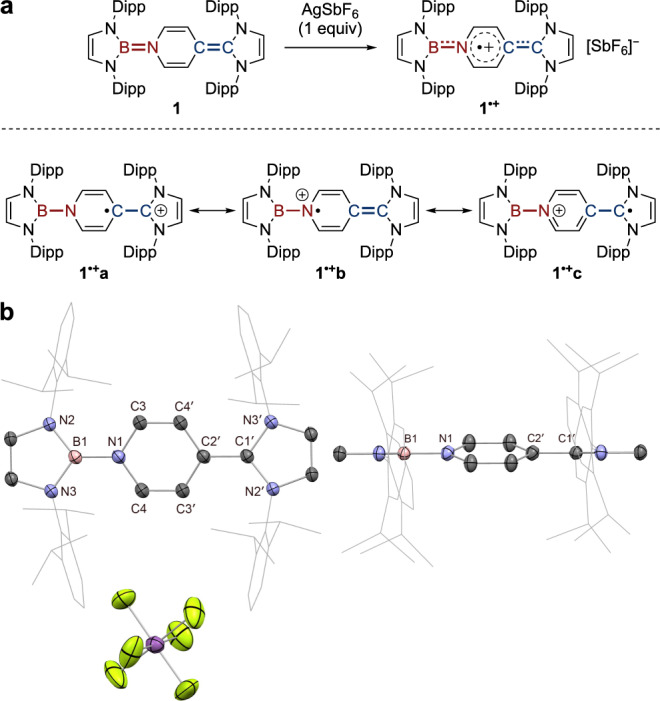
Synthesis and characterization of radical cationic organoboron analogue of Thiele’s hydrocarbon. **a** Synthesis of **1**^**•+**^, showing contributing resonance structures. **b** Solid-state structure of **1**^**•+**^**[SbF**_**6**_**]** with side view. For clarity, H atoms are omitted and Dipp groups are simplified as wireframes. Thermal ellipsoids set at 50% probability. Key distances (Å): B1−N1 1.465(4), C1’−C2’ 1.465(4), N1−C3 1.407(4), N1−C4 1.397(4), C3−C4’ 1.363(4), C3’−C4 1.363(4), C2’−C4’ 1.397(4), C2’−C3’ 1.407(4).

With this in mind, the cationic monoradical species, **1**^•**+**^, was targeted on a preparative scale by chemical oxidation. Treatment of **1** with Ag[SbF_6_] (1 equiv.) results in the immediate formation of an NMR-silent dark-brown solution (Fig. 4a). X-ray crystallography confirms its identity as the target mixed-valence radical cation **1**^•**+**^**[SbF**_**6**_**]** (Fig. 4b). Mononuclear boron radical cations are an extremely rare class of boron radical species^57–59^, in sharp contrast to their neutral and anionic counterparts^60^, presumably due to a confluence of the strong electrophilicity of a boron cation and the high reactivity of an open-shell species. Hence, **1**^•**+**^ represents a rare example of an isolable boron-containing radical cation.

**Fig. 5:**
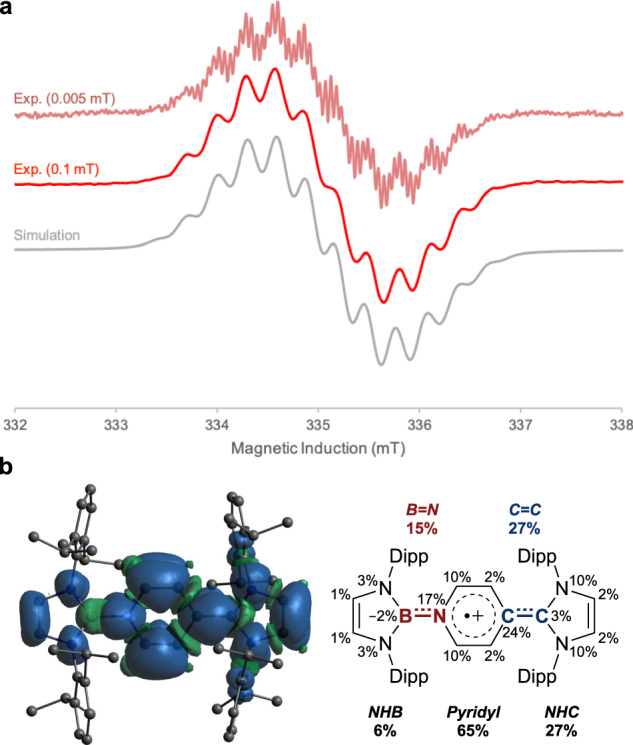
EPR studies. **a** Experimental EPR spectra at indicated field modulations (red) and simulation (grey) of **1**^•**+**^, detailed in SI. **b** Calculated spin density. The negative spin densities (ρ(α) − ρ(β) < 0), at the boron atom are the result of spin polarization of underlying orbitals by the SOMO. The polarization effect on s-character orbitals results in large Fermi-contact hyperfine interactions. A spatial difference in α and β orbitals leads to a change in the overall spin-density sign.

EPR analysis of **1**^•**+**^ (which is in good agreement with simulation) confirms the presence of an unpaired electron centred at *g* = 2.0021, which exhibits hyperfine couplings to B1 [*a*(^11^B) = 3.32 G], N1 [*a*(^14^N) = 3.14 G], N2′/N3′ [*a*(^14^N) = 2.25 G] and C3H/C4H [*a*(^1^H) = 3.18 G], hinting at the fully delocalized nature of the radical across the entire π system (Fig. 5a). Interestingly, the ^11^B hyperfine coupling constant (3.32 G) is larger than for diborene radical cations (< 1.18 G)^61,62^, and in the range of mononuclear boron radical cations (2.10 − 6.43 G)^57–59^, suggesting boron radical character in **1**^•**+**^.

Spin-density analysis confirms the delocalized picture, with a significant contribution on the central pyridyl ring (65%; Fig. 5b). Informatively, the spin population across the B=N (15%) bond is lower than the C=C (27%) bond, reflecting the more localized nature of the B=N (as opposed to C=C) bridge and resulting in an unsymmetrical distribution of the remaining spin density from the central ring onto the peripheral NHB (6%) and NHC (27%) ligands. As such, 1^•**+**^ can be described by several contributing resonance forms (Fig. 4a): (i) C-centred pyridyl radical **1**^•**+**^**a**; (ii) N-centred pyridyl radical **1**^•**+**^**b**; and (iii) NHC-centred radical **1**^•**+**^**c**. Contributions from these three resonance structures account for (i) the majority of the unpaired spin density being located on the pyridyl and NHC heterocycles; and (ii) for NRT-derived natural bond order data for **1**^•**+**^ which shows reduced bond order alternation in the central pyridyl ring compared to **1** (but greater than in **1**^**2+**^, see below), and an exocyclic C–C bond order which is also between those determined for **1** and **1**^**2+**^. It is also noteworthy that in metal-free radical borylations catalyzed by 4-substituted pyridines, a pyridine-stabilized boryl radical has been invoked as the key reactive intermediate^63–65^. **1**^•**+**^ can be regarded as a crystalline analogue of this boryl radical species.

### Synthesis of a dicationic organoboron analogue of Thiele’s hydrocarbon

**Fig. 6:**
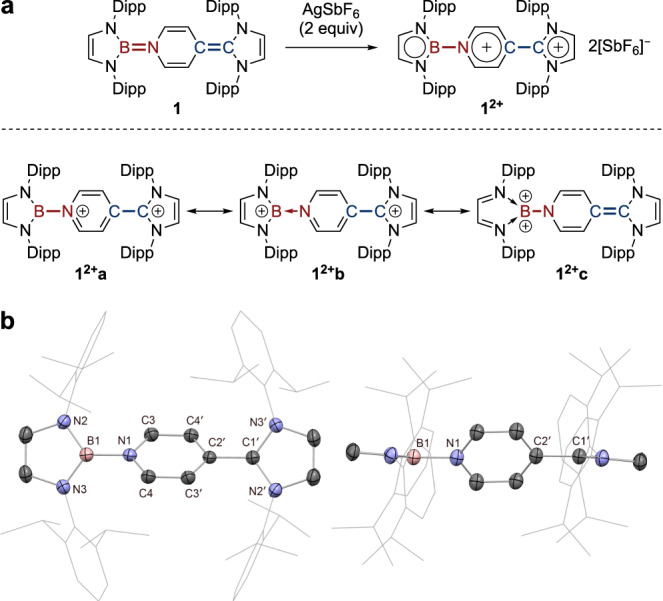
Synthesis and characterization of dicationic organoboron analogue of Thiele’s hydrocarbon. **a** Synthesis of **1**^**2+**^, showing contributing resonance structures. **b** Solid-state structure of **1**^**2+**^**[SbF**_**6**_**]**_**2**_ with side view. For clarity, [SbF_6_]^−^ counter-anions and H atoms omitted, Dipp groups simplified as wireframes. Thermal ellipsoids set at 50% probability. Key distances (Å): B1−N1 1.495(3), C1’−C2’ 1.495(3), N1−C3 1.367(3), N1−C4 1.374(3), C3−C4’ 1.375(3), C3’−C4 1.375(3), C2’−C4’ 1.374(3), C2’−C3’ 1.367(3).

Completion of the redox triad was accomplished by synthesis of the corresponding dication. Treatment of **1** with Ag[SbF_6_] (2 equiv.) results in successive colour changes from deep red to dark brown and ultimately to a persistent deep purple solution (Fig. 6a). The product is NMR-active and the ^1^H spectrum reveals a marked downfield shift of the pyridyl ring protons, suggesting increased aromaticity within the central ring, while the boron signal of the NHB unit is essentially unchanged (*δ*_B_ = 20.1 ppm). X-ray crystallography confirms its identity as the target doubly oxidized dication **1**^2**+**^**[SbF**_**6**_**]**_**2**_ (Fig. 6b). Three-coordinate borenium dications are extremely rare^66,67^, and no further redox chemistry has been reported for the isolated systems, possibly due to the presence of strongly π-donating ligands tailor-made to stabilize these highly electron-deficient species^68–70^. Hence, **1**^**2+**^ together with **1**^•**+**^ and **1** constitutes an isolable organoboron redox triad which can reversibly shuttle between dicationic, radical cationic and neutral states.

With regard to **1**^**2+**^, NPA analysis reveals the two positive charges to be highly delocalized, with boron bearing the greatest share of positive charge (+0.94). This value is larger than that determined for bis(carbodicarbene)[BH]^2+^ system (+0.36) reported by Ong et al.^66^, but close to that of bis(imino)[BPh]^2+^ dication (+1.02) reported by Inoue et al.^67^. As such, **1**^**2+**^ can also be described by several contributing resonance forms (Fig. 6a): (i) pyridinium–imidazolium **1**^**2+**^**a**; (ii) borenium–imidazolium **1**^**2+**^**b**; or (iii) borenium dication **1**^**2+**^**c**. NICS calculations are consistent with the aromatic character of the central ring suggested by the NMR measurements (NICS(0) =  −7.55; NICS(1) = −8.99), and contrast with the non-aromatic character for **1**^•+^ (NICS(0) = 1.52; NICS(1) = −0.87) and anti-aromatic character for **1** (NICS(0) = 6.67, NICS(1) = 4.14). Moreover, the natural bond orders obtained from NRT calculations (Supporting Information Table S4), suggest that **1**^**2+**^**a**/**1**^**2+**^**b** represent the overwhelmingly dominant resonance structures (exocyclic B–N/C–C single bonds; equivalent bonds within the central heterocycle).

In the solid state, both **1**^•**+**^ and **1**^**2+**^ sit on a crystallographic inversion centre (Figs. 4b and 6b). Hence, the BN- and CC-containing halves in each structure are modelled as identical fragments, and geometric discussions of **1**^•**+**^ and **1**^**2+**^ are based upon the mean values for each. Upon successive single-electron oxidations, the exocyclic B−N and C−C linkages lengthen, with accompanying reduction of the bond length alternation (BLA) in the pyridyl ring, consistent with diminishing exocyclic double-bond character and concomitant aromaticity enhancement (Table 1). This is also reflected in the progressive twisting of the central ring away from coplanarity with the peripheral NHB and NHC ligands.

**Table 1:** Mean distances (Å) and angles (°) between BN- and CC-containing fragments (DFT-calculated exocyclic bond lengths given in square parentheses).

	B1−N1/C1’−C2’	BLA (pyridyl)	Twist (pyridyl)
**2**	1.430(4), 1.356(4)	0.072(4)	10.7
**1**^**•+**^	1.465(4) [1.469, 1.416]	0.029(4)	16.4
**1**^**2+**^	1.495(3) [1.499, 1.453]	0.005(3)	46.7

**Fig. 7:**
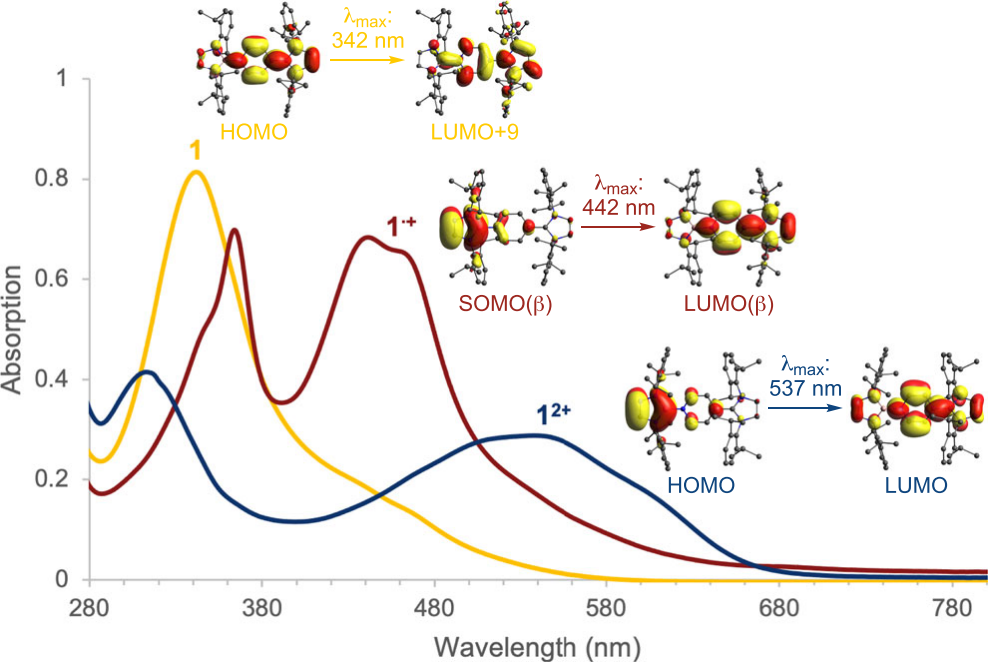
UV–vis studies. UV–vis spectra of **1**, **1**^•**+**^ and **1**^**2+**^ in CH_2_Cl_2_; key absorption assignments by TD-DFT.

The UV–vis spectra (and associated TD-DFT calculations) show a gradual red-shifting of the prominent absorption bands from **1** (*λ*_max_ = 342 nm; HOMO → LUMO+9) to **1**^•**+**^ (*λ*_max_ = 442 nm; SOMO(β) → LUMO(β)) to **1**^**2+**^ (*λ*_max_ = 537 nm; HOMO → LUMO) upon successive one-electron oxidations (Fig. 7). Interestingly, the very broad absorption band of **1**^**2+**^ (range: 400 – 700 nm) arising from the charge transfer from the NHB ligand to the central pyridyl ring, stretches across the visible region. This is in contrast to dications derived from wholly carbon systems which are typically restricted to absorptions in the UV region^6–13^, and hints at potential applications of all three states of this organoboron redox system in visible light photoredox catalysis.

To conclude, we disclose an unsymmetrical organoboron analogue of Thiele’s hydrocarbon **1**, derived from isoelectronic NHB and NHC ligands. We demonstrate that B=N substitution confers an electronic coupling enhancement of > 1 V, enabling the isolation of a boron-containing radical cation **1**^•**+**^ related to Thiele’s hydrocarbon. Further single-electron oxidation affords borenium dication **1**^**2+**^, thereby establishing a new organoboron reversible redox triad featuring **1**, **1**^•**+**^ and **1**^**2+**^. We propose that formal BN/CC substitution is critical in lowering the energy of the LUMO of the dication **1**^**2+**^ (see Supporting Information, Fig. S19) compared to its all-carbon analogue, making **1**^**2+**^ more oxidizing, and rendering the intermediate radical cation **1**^•**+**^ less prone to oxidation than its neutral counterpart **1**^13^. This hypothesis accounts for the stability of **1**^•**+**^ over an unprecedented redox range, and is consistent with the experimental observation that dicationic species derived from doubly BN-substituted systems (Fig. 1, Method **B**) are too oxidizing to be experimentally accessible. As such, we perceive that our present strategy can be extended to other transient organic radicals to imbue maximal stability and facilitate their isolation.

## Methods

### General considerations

All manipulations were carried out using standard Schlenk line or dry-box techniques under an atmosphere of argon or dinitrogen. Solvents were degassed by sparging with argon and dried by passing through a column of the appropriate drying agent. NMR spectra were measured in benzene-*d*_6_ (which was dried over potassium), with the solvent then being distilled under reduced pressure and stored under argon in Teflon valve ampoules. NMR samples were prepared under argon in 5 mm Wilmad 507-PP tubes fitted with J. Young Teflon valves. ^1^H, ^13^C{^1^H}, ^11^B{^1^H}, ^19^F{^1^H} NMR spectra were recorded on Bruker Avance III HD nanobay 400 MHz or Bruker Avance 500 MHz spectrometer at ambient temperature and referenced internally to residual protio-solvent (^1^H) or solvent (^13^C) resonances and are reported relative to tetramethylsilane (*δ* = 0 ppm). ^19^F resonances are referenced externally to CFCl_3_. Assignments were confirmed using two-dimensional ^1^H–^1^H and ^13^C–^1^H NMR correlation experiments. Chemical shifts are quoted in *δ* (ppm) and coupling constants in Hz. Elemental analyses were carried out by London Metropolitan University. (HCDippN)_2_BBr was prepared by the literature method (see Supplementary Information S1). All other reagents were used as received.

### Preparation of (HCDippN)_2_BOTf

To a mixture of (HCDippN)_2_BBr (2.00 g, 4.28 mmol) and AgOTf (1.65 g, 6.42 mmol) was added CHCl_3_ (3 mL) and stirred for 3 days at 60 °C in an ampoule. To the suspension was added benzene (20 mL) and filtered. The filtrate was dried under vacuum to yield a solid covered in a tar-like substance. To this was added *n*-hexane (250 mL) and benzene (10 mL) to dislodge the tar-like substance and filtered. The filtrate was dried under vacuum to yield (HCDippN)_2_BOTf (1.08 g, 47% yield) as a greyish-green powder. ^**1**^**H NMR** (400 MHz, C_6_D_6_, 297 K): *δ* = 1.16 (d, ^3^*J*_HH_ = 6.9 Hz, 12H, CH(C*H*_3_)_2_), 1.33 (d, ^3^*J*_HH_ = 6.9 Hz, 12H, CH(C*H*_3_)_2_), 3.16 (sept, ^3^*J*_HH_ = 6.9 Hz, 4H, C*H*(CH_3_)_2_), 6.01 (s, 2H, NC*H*), 7.10–7.12 (m, 4H, Dipp-*m*-C*H*), 7.18–7.22 (m, 2H, Dipp-*p*-C*H*); ^**11**^**B{**^**1**^**H} NMR** (128 MHz, C_6_D_6_): *δ* = 19.0; ^**19**^**F{**^**1**^**H} NMR** (377 MHz, C_6_D_6_): *δ* = −76.68. **Elemental analysis** calculated for C_27_H_36_BF_3_N_2_O_3_S: C 60.45%, H 6.76%, N 5.22%, found: C 60.11%, H 6.55%, N 4.98%.

### Preparation of 1

To a mixture of (HCDippN)_2_BOTf (200 mg, 0.37 mmol) and (HCDippN)_2_C (145 mg, 0.37 mmol) in benzene (1 mL) was added pyridine (0.1 mL, 1.24 mmol) and stirred for 5 min at room temperature to form a red solution. To the solution was added K[N(SiMe_3_)_2_] (75 mg, 0.38 mmol) at room temperature and stirred for 5 min at room temperature to form a deep red solution. Volatiles were removed under vacuum. To the residue was added benzene (5 mL) and the mixture filtered. The filtrate was dried under vacuum to yield **1** (264 mg, 83% yield) as an orange-red powder. ^**1**^**H NMR** (500 MHz, C_6_D_6_, 297 K): *δ* = 1.18 (t, ^3^*J*_HH_ = 6.6 Hz, 24H, CH(C*H*_3_)_2_), 1.30 (d, ^3^*J*_HH_ = 6.9 Hz, 12H, CH(C*H*_3_)_2_), 1.35 (d, ^3^*J*_HH_ = 6.9 Hz, 12H, CH(C*H*_3_)_2_), 3.27 (sept, ^3^*J*_HH_ = 6.9 Hz, 4H, C*H*(CH_3_)_2_), 3.38 (sept, ^3^*J*_HH_ = 6.9 Hz, 4H, C*H*(CH_3_)_2_), 4.14 (d, ^3^*J*_HH_ = 8.6 Hz, 2H, Py-C*H*), 4.81 (d, ^3^*J*_HH_ = 8.6 Hz, 2H, Py-C*H*), 5.61 (s, 2H, NC*H*), 5.80 (s, 2H, NC*H*), 6.95 (d, ^3^*J*_HH_ = 7.6 Hz, 4H, Dipp-*m*-C*H*), 7.01–7.08 (m, 8H, Dipp-C*H*); ^**13**^**C{**^**1**^**H} NMR** (126 MHz, C_6_D_6_): *δ* = 23.3 (CH(*C*H_3_)_2_), 23.7 (CH(*C*H_3_)_2_), 24.5 (CH(*C*H_3_)_2_), 24.7 (CH(*C*H_3_)_2_), 28.7 (*C*H(CH_3_)_2_), 81.5 (Py-*p*-*C*), 107.6 (Py-*C*H), 117.3 (N*C*H), 118.5 (N*C*H), 121.1 (Py-*C*H), 123.6 (Dipp-*m*-*C*H), 123.9 (Dipp-*m*-*C*H), 127.6 (Dipp-*p*-*C*H), 128.7 (Dipp-*p*-*C*H), 132.4 (N*C*N), 137.7 (Dipp-*i*-*C*), 139.8 (Dipp-*i*-*C*), 146.3 (Dipp-*o*-*C*), 147.7 (Dipp-*o*-*C*); ^**11**^**B{**^**1**^**H} NMR** (128 MHz, C_6_D_6_): *δ* = 19.9; **UV–vis** (CH_2_Cl_2_, *λ*_max_): 342 nm (*ε* = 16284 M^−1^cm^−1^).

### Preparation of 2

To a mixture of (HCDippN)_2_BOTf (200 mg, 0.37 mmol) and (HCMesN)_2_C (114 mg, 0.37 mmol) in benzene (1 mL) was added pyridine (0.1 mL, 1.24 mmol) and stirred for 5 min at room temperature to form a red solution. To the solution was added K[N(SiMe_3_)_2_] (75 mg, 0.38 mmol) at room temperature and stirred for 5 min at room temperature to form a deep red solution. Volatiles were removed under vacuum. To the residue was added *n*-hexane (25 mL) and the mixture filtered. The filtrate was dried under vacuum to yield **2** (159 mg, 56% yield) as an orange-red powder. Single crystals (yellow plates) suitable for X-ray crystallography were obtained by slow evaporation of a concentrated solution of **2** in *n*-pentane at room temperature. ^**1**^**H NMR** (500 MHz, C_6_D_6_, 297 K): *δ* = 1.20 (d, ^3^*J*_HH_ = 6.9 Hz, 12H, CH(C*H*_3_)_2_), 1.29 (d, ^3^*J*_HH_ = 6.9 Hz, 12H, CH(C*H*_3_)_2_), 2.07 (s, 6H, Mes-*p*-CC*H*_3_), 2.21 (s, 12H, Mes-*o*-CC*H*_3_), 3.32 (sept, ^3^*J*_HH_ = 6.9 Hz, 4H, C*H*(CH_3_)_2_), 4.28 (d, ^3^*J*_HH_ = 8.4 Hz, 2H, Py-C*H*), 4.92 (d, ^3^*J*_HH_ = 8.4 Hz, 2H, Py-C*H*), 5.47 (s, 2H, NC*H*), 5.83 (s, 2H, NC*H*), 6.57 (s, 4H, Mes-*m*-C*H*), 6.99–7.01 (m, 4H, Dipp-C*H*), 7.05–7.08 (m, 2H, Dipp-C*H*); ^**13**^**C{**^**1**^**H} NMR** (126 MHz, C_6_D_6_): *δ* = 18.4 (Mes-*o*-C*C*H_3_), 21.0 (Mes-*p*-C*C*H_3_), 23.6 (CH(*C*H_3_)_2_), 24.7 (CH(*C*H_3_)_2_), 28.7 (*C*H(CH_3_)_2_), 81.5 (Py-*p*-*C*), 107.6 (Py-*C*H), 116.3 (N*C*H), 118.4 (N*C*H), 121.4 (Py-*C*H), 123.6 (Dipp-*m*-*C*H), 127.6 (Dipp-*p*-*C*H), 129.3 (Mes-*m*-*C*H), 131.2 (N*C*N), 136.5 (Mes-*p*-*C*), 136.6 (Mes-*o*-*C*), 136.9 (Mes-*i*-*C*), 140.0 (Dipp-*i*-*C*), 146.3 (Dipp-*o*-*C*); ^**11**^**B{**^**1**^**H} NMR** (128 MHz, C_6_D_6_): *δ* = 20.5; **Elemental analysis** calculated for C_52_H_64_BN_5_: C 81.12%, H 8.38%, N 9.10%, found: C 80.91%, H 8.20%, N 8.89%.

### Preparation of [1][SbF_6_]

To a mixture of **1** (300 mg, 0.35 mmol) and AgSbF_6_ (121 mg, 0.35 mmol) was added CH_2_Cl_2_ (3 mL) and stirred for 5 min at room temperature to form a dark-brown solution. The mixture was filtered and the filtrate was dried under vacuum to yield **[1][SbF**_**6**_**]** (325 mg, 85% yield) as a dark-brown powder. Single crystals (brown rods and plates) suitable for X-ray crystallography were obtained by slow evaporation of a concentrated solution of **[1][SbF**_**6**_**]** in fluorobenzene at room temperature. **X-band EPR** g = 2.0021 (1xB: −9.3 MHz; 1xN_Py_: 8.8 MHz; 2xN_IDipp_: 6.3 MHz; 2xH_Py-*o*-C*H*_: −8.9 MHz); **UV–vis** (CH_2_Cl_2_, *λ*_max_): 364 nm (*ε* = 13983 M^−1^ cm^−1^), 442 nm (*ε* = 13682 M^−1^cm^−1^); **Elemental analysis** calculated for C_58_H_76_BF_6_N_5_Sb: C 63.92%, H 7.03%, N 6.43%, found: C 63.77%, H 7.13%, N 6.29%.

### Preparation of [1][SbF_6_]_2_

To a mixture of **1** (50 mg, 0.06 mmol) and AgSbF_6_ (40 mg, 0.12 mmol) was added CH_2_Cl_2_ (2 mL) and stirred for 5 min at room temperature to form a deep purple solution. The mixture was filtered and the filtrate was dried under vacuum to yield **[1][SbF**_**6**_**]**_**2**_ (43 mg, 56% yield) as a purple powder. Single crystals (purple rods) suitable for X-ray crystallography were obtained by slow evaporation of a concentrated solution of **[1][SbF**_**6**_**]**_**2**_ in CH_2_Cl_2_at room temperature. ^**1**^**H NMR** (400 MHz, CD_2_Cl_2_, 297 K): *δ* = 0.85 (d, ^3^*J*_HH_ = 6.9 Hz, 12H, CH(C*H*_3_)_2_), 0.91 (d, ^3^*J*_HH_ = 6.8 Hz, 12H, CH(C*H*_3_)_2_), 1.17 (d, ^3^*J*_HH_ = 6.8 Hz, 12H, CH(C*H*_3_)_2_), 1.22 (d, ^3^*J*_HH_ = 6.7 Hz, 12H, CH(C*H*_3_)_2_), 2.21 (sept, ^3^*J*_HH_ = 6.8 Hz, 4H, C*H*(CH_3_)_2_), 2.66 (sept, ^3^*J*_HH_ = 6.8 Hz, 4H, C*H*(CH_3_)_2_), 6.56 (s, 2H, NC*H*), 7.14 (d, ^3^*J*_HH_ = 7.2 Hz, 2H, Py-C*H*), 7.27 (d, ^3^*J*_HH_ = 7.8 Hz, 4H, Dipp-*m*-C*H*), 7.38 (d, ^3^*J*_HH_ = 7.9 Hz, 4H, Dipp-*m*-C*H*), 7.47 (t, ^3^*J*_HH_ = 7.8 Hz, 2H, Dipp-*p*-C*H*), 7.69 (t, ^3^*J*_HH_ = 7.9 Hz, 2H, Dipp-*p*-C*H*), 7.97 (d, ^3^*J*_HH_ = 7.2 Hz, 2H, Py-C*H*), 8.03 (s, 2H, NC*H*); ^**13**^**C{**^**1**^**H} NMR** (126 MHz, CD_2_Cl_2_): *δ* = 22.7 (CH(*C*H_3_)_2_), 23.4 (CH(*C*H_3_)_2_), 25.2 (CH(*C*H_3_)_2_), 25.5 (CH(*C*H_3_)_2_), 29.1 (*C*H(CH_3_)_2_), 29.9 (*C*H(CH_3_)_2_), 122.7 (N*C*H), 125.6 (Dipp-*m*-*C*H), 126.8 (Dipp-*m*-*C*H), 127.3 (Py-*C*H), 128.8 (Dipp-*i*-*C*), 130.1 (N*C*H), 130.6 (Dipp-*p*-*C*H), 133.4 (Dipp-*i*-*C*), 134.4 (Dipp-*p*-*C*H), 137.3 (Py-*p*-*C*), 137.4 (N*C*N), 144.7 (Dipp-*o*-*C*), 145.3 (Py-*C*H), 145.4 (Dipp-*o*-*C*); ^**11**^**B{**^**1**^**H} NMR** (128 MHz, CD_2_Cl_2_): *δ* = 20.1; **UV–vis** (CH_2_Cl_2_, *λ*_max_): 313 nm (*ε* = 8294 M^−1^ cm^−1^), 537 nm (*ε* = 5756 M^−1^ cm^−1^); **Elemental analysis** for C_58_H_76_BF_12_N_5_Sb_2_: C 52.55%, H 5.78%, N 5.28%, found: C 52.63%, H 5.51%, N 5.30%.

## Supplementary information

Supplementary Information

Peer Review File

## Data Availability

The crystallographic data generated in this study have been deposited in the CCDC structural database under accession codes 2063252-2063254 [www.ccdc.cam.ac.uk]. Spectroscopic data are available in the Supplementary Information and through the Oxford Research Archive (www.ora.ox.ac.uk).
