# Supplementary material for: A crystalline radical cation derived from Thiele’s hydrocarbon with redox range beyond 1 V
Source: Nat Commun. 2021 Dec 3;12:7052. doi: 10.1038/s41467-021-27104-y (PMC8642399; doi:10.1038/s41467-021-27104-y)
Supplement: Supplementary file 1 — Supplementary Information [file 41467_2021_27104_MOESM1_ESM.pdf]

# A Crystalline Radical Cation Derived from Thiele's Hydrocarbon with Redox Range Beyond 1 V

Ying Kai Loh,[a] Petra Vasko,[b] Caitilín McManus,[a] Andreas Heilmann,[a] William K Myers,[a] Simon Aldridge*[a]

[a] Inorganic Chemistry Laboratory, Dept of Chemistry, University of Oxford, South Parks Road, Oxford OX1 3QR, UK.
[b] Department of Chemistry, Nanoscience Center, University of Jyväskylä, P. O. Box 35, Jyväskylä, Finland FI-40014.
* simon.aldridge@chem.ox.ac.uk

## Supplementary Information

**Table of Contents**

1. General considerations and starting material preparations s2

2. Synthetic, spectroscopic and analytical data – $(HCDippN)_2BOTf$, 1, 2, 1[$SbF_6$], 1[$SbF_6$]$_2$ s3

3. NMR spectra – $(HCDippN)_2BOTf$, 1, 2, 1[$SbF_6$], 1[$SbF_6$]$_2$ s5

4. Cyclic voltammetric studies s13

5. EPR studies s14

6. UV-vis studies s15

7. X-ray crystallographic studies s17

8. Computational studies s19

9. Supplementary references s32

## 1. General considerations and starting material preparation

All manipulations were carried out using standard Schlenk line or dry-box techniques under an atmosphere of argon or dinitrogen. Solvents were degassed by sparging with argon and dried by passing through a column of the appropriate drying agent. NMR spectra were measured in benzene-$d_6$ (which was dried over potassium), with the solvent then being distilled under reduced pressure and stored under argon in Teflon valve ampoules. NMR samples were prepared under argon in 5 mm Wilmad 507-PP tubes fitted with J. Young Teflon valves. $^{1}H$, $^{13}C\{^{1}H\}$, $^{11}B\{^{1}H\}$, $^{19}F\{^{1}H\}$ NMR spectra were recorded on Bruker Avance III HD nanobay 400 MHz or Bruker Avance 500 MHz spectrometer at ambient temperature and referenced internally to residual protio-solvent ($^{1}H$) or solvent ($^{13}C$) resonances and are reported relative to tetramethylsilane ($\delta$ = 0 ppm). $^{19}F$ resonances are referenced externally to $CFCl_3$. Assignments were confirmed using two-dimensional $^{1}H$-$^{1}H$ and $^{13}C$-$^{1}H$ NMR correlation experiments. Chemical shifts are quoted in $\delta$ (ppm) and coupling constants in Hz. Elemental analyses were carried out by London Metropolitan University. $(HCDippN)_2BBr$ was prepared by the literature method (see below).[s1] All other reagents were used as received.

literature synthesis of $(HCDippN)_2BBr$: A mixture of *N,N'*-bis(diisopropylphenyl)-1,4-diazabutadiene (4.00 g, 10.6 mmol) and $Ph_3P{\cdot}BBr_3$ (5.45 g, 10.6 mmol) in diethyl (60 mL) was refluxed for 18 h. Solvents were removed in vacuo and the residue extracted with pentane (80 mL). After filtration, concentration of the filtrate in vacuo (to ca. 15 mL), and storage at –30 °C, $(HCDippN)_2BBr$ was obtained as yellow-orange crystals (70-75 %).

## 2. Synthetic, spectroscopic and analytical data

***Preparation of (HCDippN)$_2$BOTf***. To a mixture of (HCDippN)$_2$BBr (2.00 g, 4.28 mmol) and AgOTf (1.65 g, 6.42 mmol) was added $CHCl_3$ (3 mL) and stirred for 3 days at 60 ºC in an ampoule. To the suspension was added benzene (20 mL) and filtered. The filtrate was dried under vacuum to yield a solid covered in a tar-like substance. To this was added *n*-hexane (250 mL) and benzene (10 mL) to dislodge the tar-like substance and filtered. The filtrate was dried under vacuum to yield (HCDippN)$_2$BOTf (1.08 g, 47% yield) as a greyish-green powder. **$^1$H NMR** (400 MHz, $C_6D_6$, 297 K): δ = 1.16 (d, $^3J_{HH}$ = 6.9 Hz, 12H, CH(C*H*$_3$)$_2$), 1.33 (d, $^3J_{HH}$ = 6.9 Hz, 12H, CH(C*H*$_3$)$_2$), 3.16 (sept, $^3J_{HH}$ = 6.9 Hz, 4H, C*H*(CH$_3$)$_2$), 6.01 (s, 2H, NC*H*), 7.10−7.12 (m, 4H, Dipp-*m*-C*H*), 7.18−7.22 (m, 2H, Dipp-*p*-C*H*); **$^{11}$B{$^1$H} NMR** (128 MHz, $C_6D_6$): δ = 19.0; **$^{13}$C{$^1$H} NMR** (126 MHz, $C_6D_6$): δ = 22.5 (CH(*C*H$_3$)$_2$), 24.6 (CH(*C*H$_3$)$_2$), 29.1 (*C*H(CH$_3$)$_2$), 116.7 (N*C*H), 123.2 (Dipp-*m*-*C*H), 128.4 (Dipp-*p*-*C*H), 139.1 (Dipp-*i*-*C*), 148.1 (Dipp-*o*-*C*); **$^{19}$F{$^1$H} NMR** (377 MHz, $C_6D_6$): δ = −76.68. **Elemental analysis** calculated for $C_{27}H_{36}BF_3N_2O_3S$: C 60.45%, H 6.76%, N 5.22%, found: C 60.11%, H 6.55%, N 4.98%.

***Preparation of (HCDippN)$_2$B(NC$_4$H$_4$C)C(NDippCH)$_2$ [1]***. To a mixture of (HCDippN)$_2$BOTf (200 mg, 0.37 mmol) and (HCDippN)$_2$C (145 mg, 0.37 mmol) in benzene (1 mL) was added pyridine (0.1 mL, 1.24 mmol) and stirred for 5 min at room temperature to form a red solution. To the solution was added K[N(SiMe$_3$)$_2$] (75 mg, 0.38 mmol) at room temperature and stirred for 5 min at room temperature to form a deep red solution. Volatiles were removed under vacuum. To the residue was added benzene (5 mL) and the mixture filtered. The filtrate was dried under vacuum to yield **1** (264 mg, 83% yield) as an orange-red powder. **$^1$H NMR** (500 MHz, $C_6D_6$, 297 K): δ = 1.18 (t, $^3J_{HH}$ = 6.6 Hz, 24H, CH(C*H*$_3$)$_2$), 1.30 (d, $^3J_{HH}$ = 6.9 Hz, 12H, CH(C*H*$_3$)$_2$), 1.35 (d, $^3J_{HH}$ = 6.9 Hz, 12H, CH(C*H*$_3$)$_2$), 3.27 (sept, $^3J_{HH}$ = 6.9 Hz, 4H, C*H*(CH$_3$)$_2$), 3.38 (sept, $^3J_{HH}$ = 6.9 Hz, 4H, C*H*(CH$_3$)$_2$), 4.14 (d, $^3J_{HH}$ = 8.6 Hz, 2H, Py-C*H*), 4.81 (d, $^3J_{HH}$ = 8.6 Hz, 2H, Py-C*H*), 5.61 (s, 2H, NC*H*), 5.80 (s, 2H, NC*H*), 6.95 (d, $^3J_{HH}$ = 7.6 Hz, 4H, Dipp-*m*-C*H*), 7.01−7.08 (m, 8H, Dipp-C*H*); **$^{13}$C{$^1$H} NMR** (126 MHz, $C_6D_6$): δ = 23.3 (CH(*C*H$_3$)$_2$), 23.7 (CH(*C*H$_3$)$_2$), 24.5 (CH(*C*H$_3$)$_2$), 24.7 (CH(*C*H$_3$)$_2$), 28.7 (*C*H(CH$_3$)$_2$), 81.5 (Py-*p*-*C*), 107.6 (Py-*C*H), 117.3 (N*C*H), 118.5 (N*C*H), 121.1 (Py-*C*H), 123.6 (Dipp-*m*-*C*H), 123.9 (Dipp-*m*-*C*H), 127.6 (Dipp-*p*-*C*H), 128.7 (Dipp-*p*-*C*H), 132.4 (N*C*N), 137.7 (Dipp-*i*-*C*), 139.8 (Dipp-*i*-*C*), 146.3 (Dipp-*o*-*C*), 147.7 (Dipp-*o*-*C*); **$^{11}$B{$^1$H} NMR** (128 MHz, $C_6D_6$): δ = 19.9; **UV-vis** ($CH_2Cl_2$, $\lambda_{max}$): 342 nm (ε = 16284 $M^{-1}cm^{-1}$).

***Preparation of (HCDippN)$_2$B(NC$_4$H$_4$C)C(NMesCH)$_2$ [2]***. To a mixture of (HCDippN)$_2$BOTf (200 mg, 0.37 mmol) and (HCMesN)$_2$C (114 mg, 0.37 mmol) in benzene (1 mL) was added pyridine (0.1 mL, 1.24 mmol) and stirred for 5 min at room temperature to form a red solution. To the solution was added K[N(SiMe$_3$)$_2$] (75 mg, 0.38 mmol) at room temperature and stirred for 5 min at room temperature to form a deep red solution. Volatiles were removed under vacuum. To the residue was added *n*-hexane (25 mL) and the mixture filtered. The filtrate was dried under vacuum to yield **2** (159 mg, 56% yield) as an orange-red powder. Single crystals (yellow plates) suitable for X-ray crystallography were obtained by slow evaporation of a concentrated solution of **2** in *n*-pentane at room temperature. **$^1$H NMR** (500 MHz, $C_6D_6$, 297 K): δ = 1.20 (d, $^3J_{HH}$ = 6.9 Hz, 12H, CH(C*H*$_3$)$_2$), 1.29 (d, $^3J_{HH}$ = 6.9 Hz, 12H, CH(C*H*$_3$)$_2$), 2.07 (s, 6H, Mes-*p*-CC*H*$_3$), 2.21 (s, 12H, Mes-*o*-CC*H*$_3$), 3.32 (sept, $^3J_{HH}$ = 6.9 Hz, 4H, C*H*(CH$_3$)$_2$), 4.28 (d, $^3J_{HH}$ = 8.4 Hz, 2H, Py-C*H*), 4.92 (d, $^3J_{HH}$ = 8.4 Hz, 2H, Py-C*H*), 5.47 (s, 2H, NC*H*), 5.83 (s, 2H, NC*H*), 6.57 (s, 4H, Mes-*m*-C*H*), 6.99−7.01 (m, 4H, Dipp-C*H*), 7.05−7.08 (m, 2H, Dipp-C*H*); **$^{13}$C{$^1$H} NMR** (126 MHz, $C_6D_6$): δ = 18.4 (Mes-*o*-C*C*H$_3$), 21.0 (Mes-*p*-C*C*H$_3$), 23.6 (CH(*C*H$_3$)$_2$), 24.7 (CH(*C*H$_3$)$_2$), 28.7 (*C*H(CH$_3$)$_2$), 81.5 (Py-*p*-*C*), 107.6 (Py-*C*H), 116.3 (N*C*H), 118.4 (N*C*H), 121.4 (Py-*C*H), 123.6 (Dipp-*m*-*C*H), 127.6 (Dipp-*p*-*C*H), 129.3 (Mes-*m*-*C*H), 131.2 (N*C*N), 136.5 (Mes-*p*-*C*), 136.6 (Mes-*o*-*C*), 136.9 (Mes-*i*-*C*), 140.0 (Dipp-*i*-*C*), 146.3 (Dipp-*o*-*C*); **$^{11}$B{$^1$H} NMR** (128 MHz, $C_6D_6$): δ = 20.5; **Elemental analysis** calculated for $C_{52}H_{64}BN_5$: C 81.12%, H 8.38%, N 9.10%, found: C 80.91%, H 8.20%, N 8.89%.

***Preparation of [(HCDippN)$_2$B(NC$_4$H$_4$C)C(NDippCH)$_2$][SbF$_6$] [1][SbF$_6$]***. To a mixture of **1** (300 mg, 0.35 mmol) and $AgSbF_6$ (121 mg, 0.35 mmol) was added $CH_2Cl_2$ (3 mL) and stirred for 5 min at room temperature to form a dark brown solution. The mixture was filtered and the filtrate was dried under vacuum to yield **[1][SbF$_6$]** (325 mg, 85% yield) as a

dark brown powder. Single crystals (brown rods and plates) suitable for X-ray crystallography were obtained by slow evaporation of a concentrated solution of **[1][SbF$_6$]** in fluorobenzene at room temperature. **X-band EPR** g = 2.0021 (1xB: −9.3 MHz; 1xN$_{Py}$: 8.8 MHz; 2xN$_{IDipp}$: 6.3 MHz; 2xH$_{Py\text{-}o\text{-}CH}$: −8.9 MHz); **UV-vis** (CH$_2$Cl$_2$, $\lambda_{max}$): 364 nm ($\varepsilon$ = 13983 M$^{-1}$cm$^{-1}$), 442 nm ($\varepsilon$ = 13682 M$^{-1}$cm$^{-1}$); **Elemental analysis** calculated for $C_{58}H_{76}BF_6N_5Sb$: C 63.92%, H 7.03%, N 6.43%, found: C 63.77%, H 7.13%, N 6.29%.

***Preparation of [(HCDippN)$_2$B(NC$_4$H$_4$C)C(NDippCH)$_2$][SbF$_6$]$_2$ [1][SbF$_6$]$_2$*.** To a mixture of **1** (50 mg, 0.06 mmol) and $AgSbF_6$ (40 mg, 0.12 mmol) was added $CH_2Cl_2$ (2 mL) and stirred for 5 min at room temperature to form a deep purple solution. The mixture was filtered and the filtrate was dried under vacuum to yield **[1][SbF$_6$]$_2$** (43 mg, 56% yield) as a purple powder. Single crystals (purple rods) suitable for X-ray crystallography were obtained by slow evaporation of a concentrated solution of **[1][SbF$_6$]$_2$** in $CH_2Cl_2$at room temperature. **$^1$H NMR** (400 MHz, CD$_2$Cl$_2$, 297 K): $\delta$ = 0.85 (d, $^3J_{HH}$ = 6.9 Hz, 12H, CH(C*H*$_3$)$_2$), 0.91 (d, $^3J_{HH}$ = 6.8 Hz, 12H, CH(C*H*$_3$)$_2$), 1.17 (d, $^3J_{HH}$ = 6.8 Hz, 12H, CH(C*H*$_3$)$_2$), 1.22 (d, $^3J_{HH}$ = 6.7 Hz, 12H, CH(C*H*$_3$)$_2$), 2.21 (sept, $^3J_{HH}$ = 6.8 Hz, 4H, C*H*(CH$_3$)$_2$), 2.66 (sept, $^3J_{HH}$ = 6.8 Hz, 4H, C*H*(CH$_3$)$_2$), 6.56 (s, 2H, NC*H*), 7.14 (d, $^3J_{HH}$ = 7.2 Hz, 2H, Py-C*H*), 7.27 (d, $^3J_{HH}$ = 7.8 Hz, 4H, Dipp-*m*-C*H*), 7.38 (d, $^3J_{HH}$ = 7.9 Hz, 4H, Dipp-*m*-C*H*), 7.47 (t, $^3J_{HH}$ = 7.8 Hz, 2H, Dipp-*p*-C*H*), 7.69 (t, $^3J_{HH}$ = 7.9 Hz, 2H, Dipp-*p*-C*H*), 7.97 (d, $^3J_{HH}$ = 7.2 Hz, 2H, Py-C*H*), 8.03 (s, 2H, NC*H*); **$^{13}$C{$^1$H} NMR** (126 MHz, CD$_2$Cl$_2$): $\delta$ = 22.7 (CH(*C*H$_3$)$_2$), 23.4 (CH(*C*H$_3$)$_2$), 25.2 (CH(*C*H$_3$)$_2$), 25.5 (CH(*C*H$_3$)$_2$), 29.1 (*C*H(CH$_3$)$_2$), 29.9 (*C*H(CH$_3$)$_2$), 122.7 (N*C*H), 125.6 (Dipp-*m*-*C*H), 126.8 (Dipp-*m*-*C*H), 127.3 (Py-*C*H), 128.8 (Dipp-*i*-*C*), 130.1 (N*C*H), 130.6 (Dipp-*p*-*C*H), 133.4 (Dipp-*i*-*C*), 134.4 (Dipp-*p*-*C*H), 137.3 (Py-*p*-*C*), 137.4 (N*C*N), 144.7 (Dipp-*o*-*C*), 145.3 (Py-*C*H), 145.4 (Dipp-*o*-*C*); **$^{11}$B{$^1$H} NMR** (128 MHz, CD$_2$Cl$_2$): $\delta$ = 20.1; **UV-vis** (CH$_2$Cl$_2$, $\lambda_{max}$): 313 nm ($\varepsilon$ = 8294 M$^{-1}$cm$^{-1}$), 537 nm ($\varepsilon$ = 5756 M$^{-1}$cm$^{-1}$); **Elemental analysis** for $C_{58}H_{76}BF_{12}N_5Sb_2$: C 52.55%, H 5.78%, N 5.28%, found: C 52.63%, H 5.51%, N 5.30%.

## 3. NMR spectra

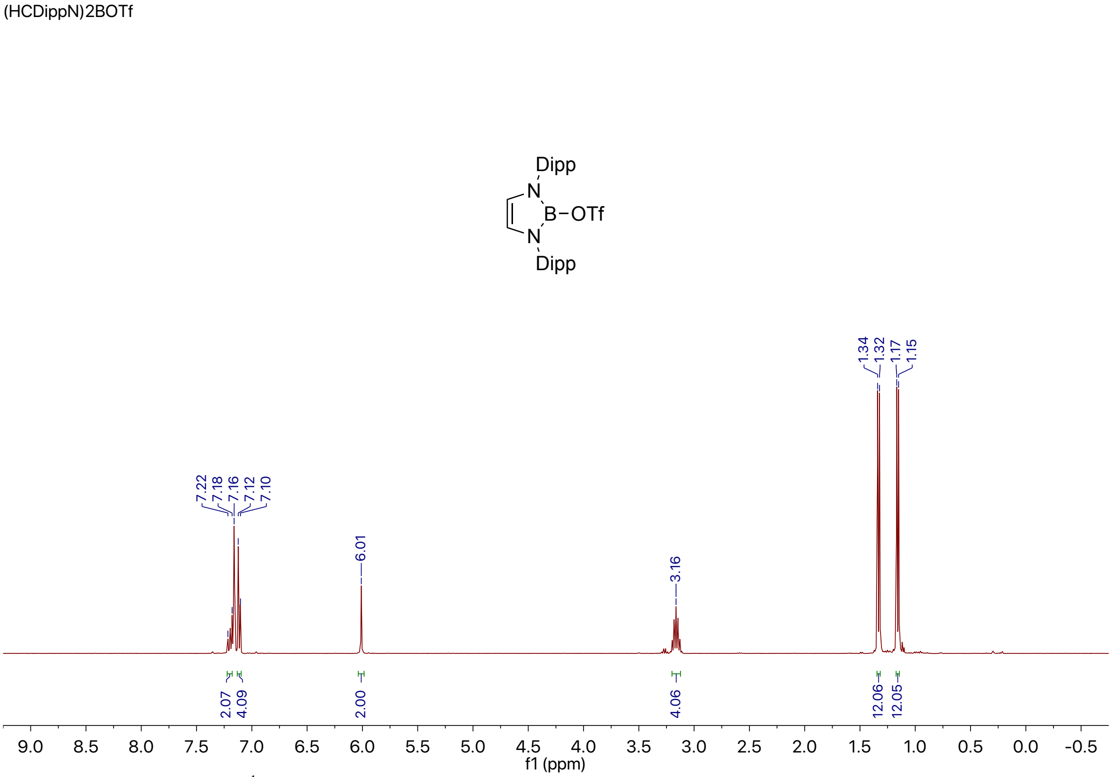


**Supplementary Figure 1.** $^{1}$H NMR spectrum of (HCDippN)$_2$BOTf (400 MHz, $C_6D_6$).

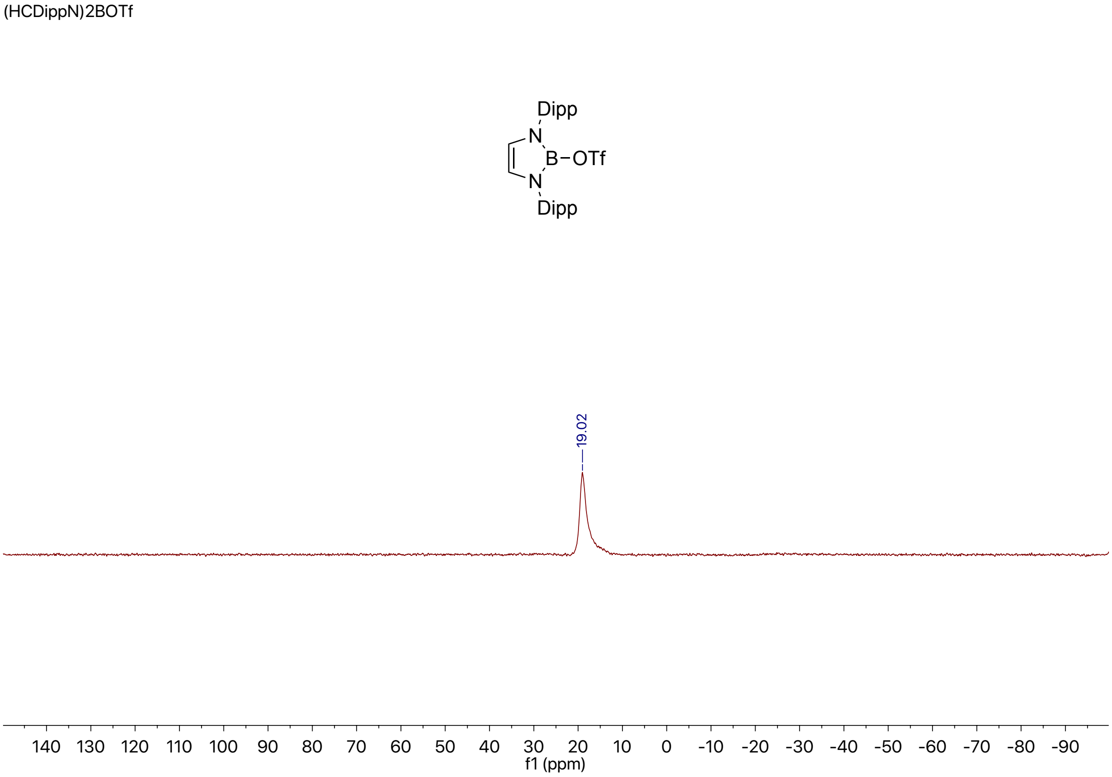


**Supplementary Figure 2.** $^{11}$B{$^{1}$H} NMR spectrum of (HCDippN)$_2$BOTf (128 MHz, $C_6D_6$).

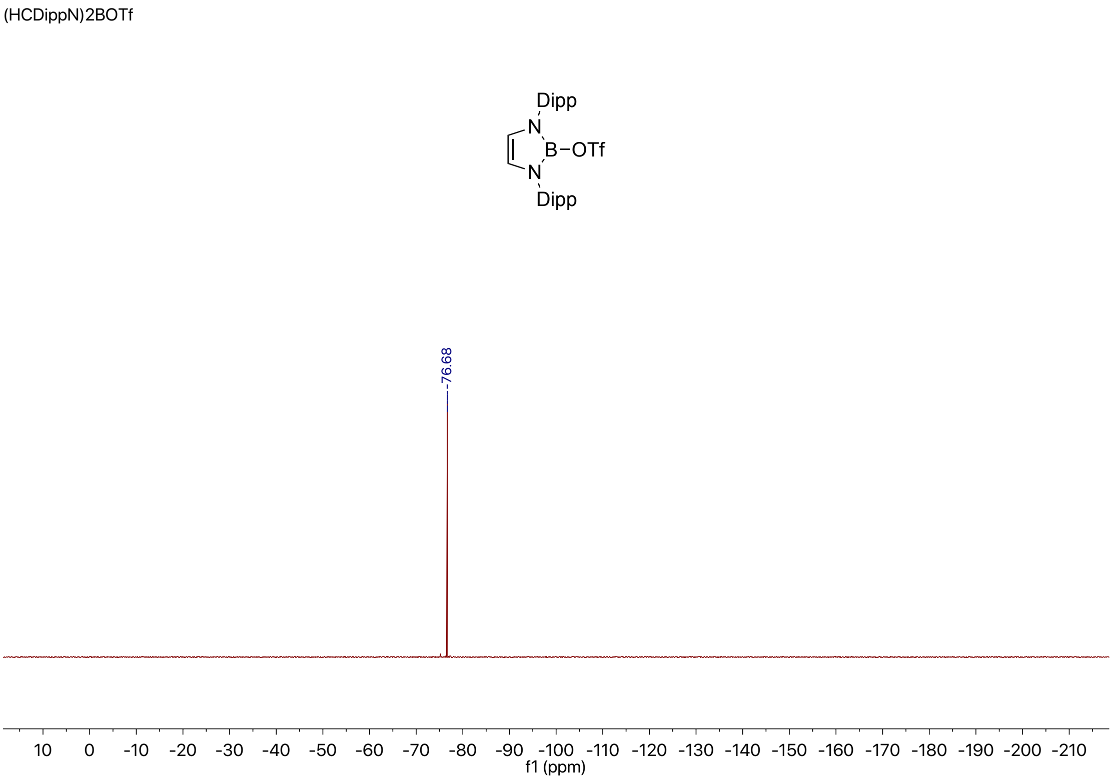


**Supplementary Figure 3.** $^{19}F\{^{1}H\}$ NMR spectrum of $(HCDippN)_2BOTf$ (377 MHz, $C_6D_6$).

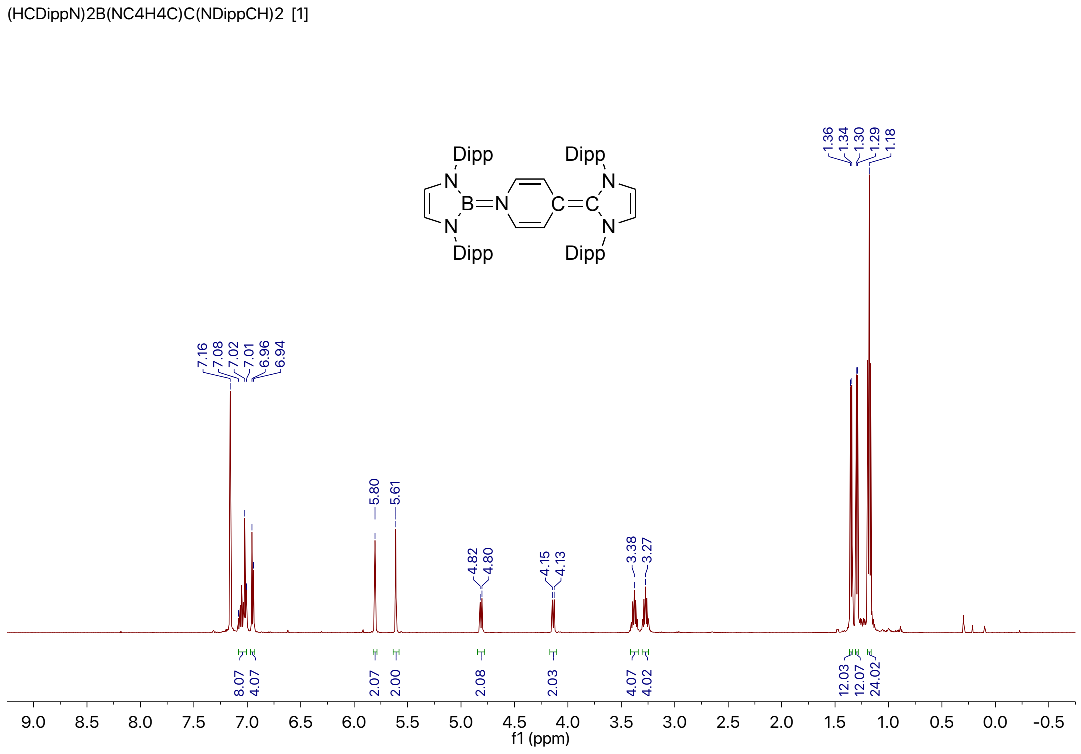


**Supplementary Figure 4.** $^1$H NMR spectrum of **1** (500 MHz, $C_6D_6$).

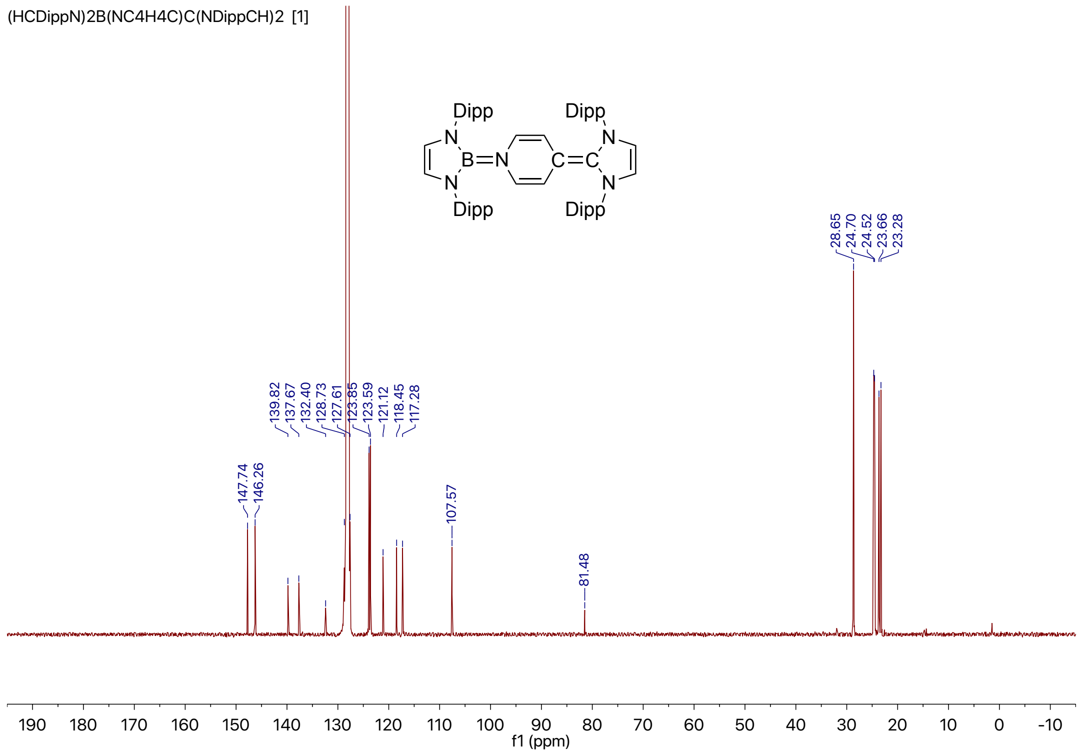


**Supplementary Figure 5.** $^{13}$C{$^1$H} NMR spectrum of **1** (126 MHz, $C_6D_6$).

(HCDippN)2B(NC4H4C)C(NDippCH)2 [1]

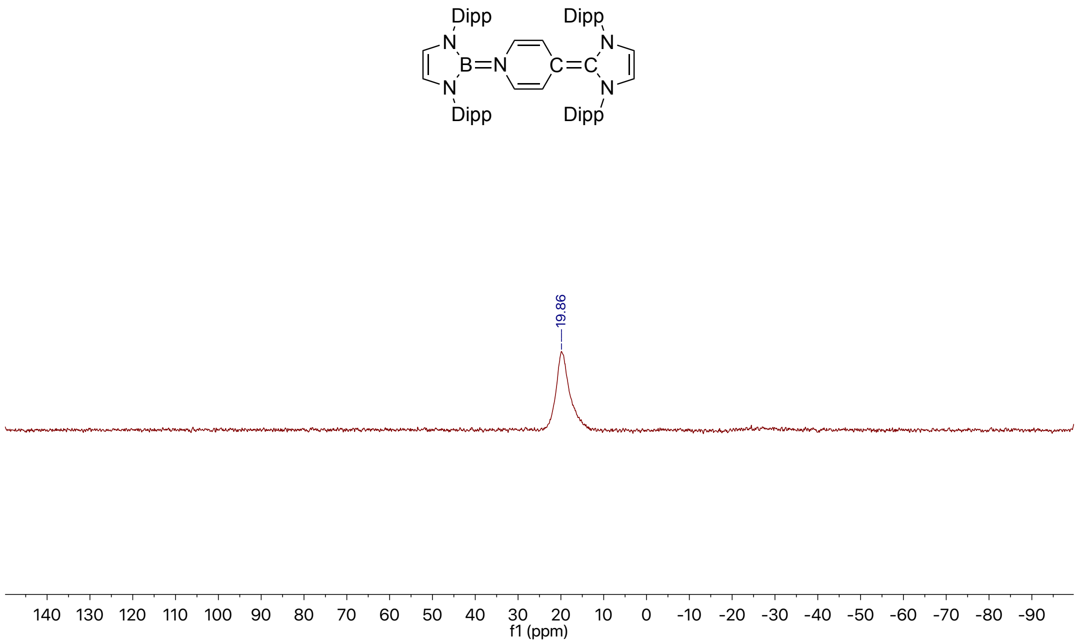


**Supplementary Figure 6.** $^{11}B\{^{1}H\}$ NMR spectrum of **1** (128 MHz, $C_6D_6$).

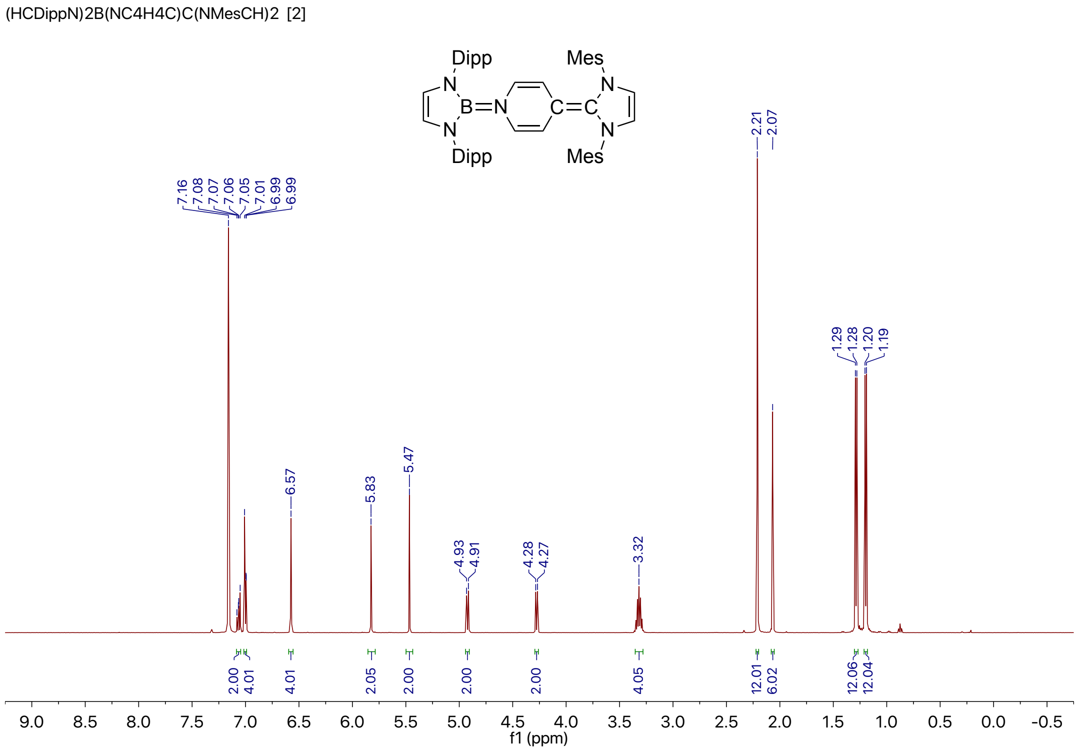


**Supplementary Figure 7.** $^{1}$H NMR spectrum of **2** (500 MHz, $C_6D_6$).

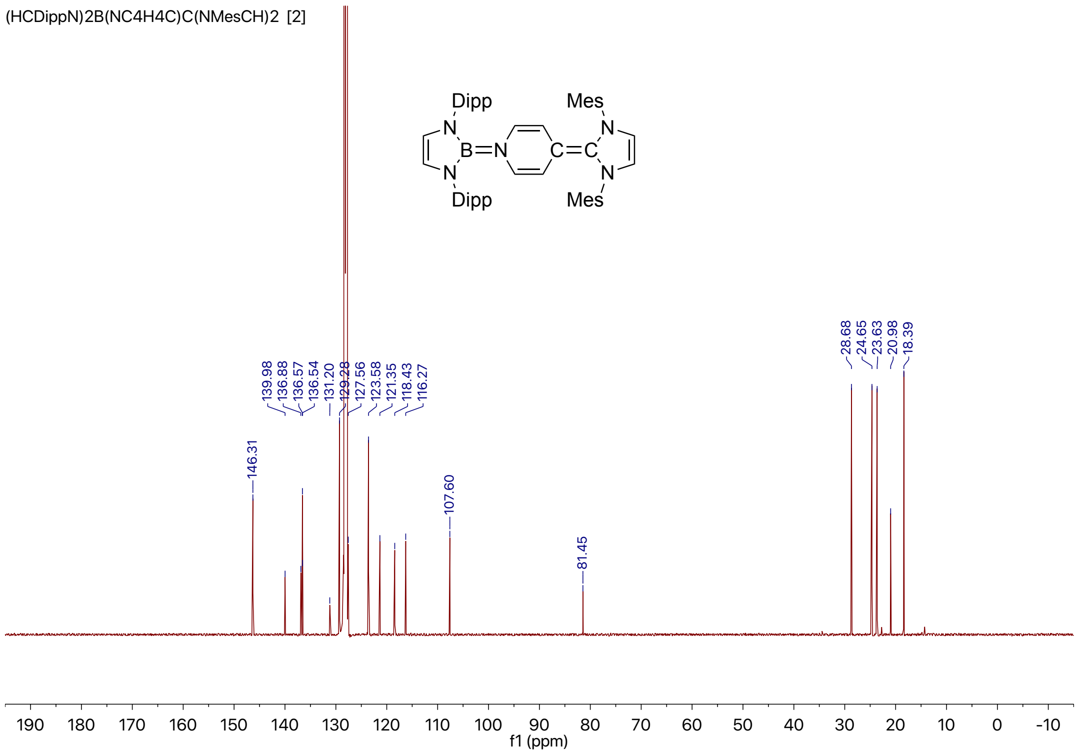


**Supplementary Figure 8.** $^{13}$C{$^{1}$H} NMR spectrum of **2** (126 MHz, $C_6D_6$).

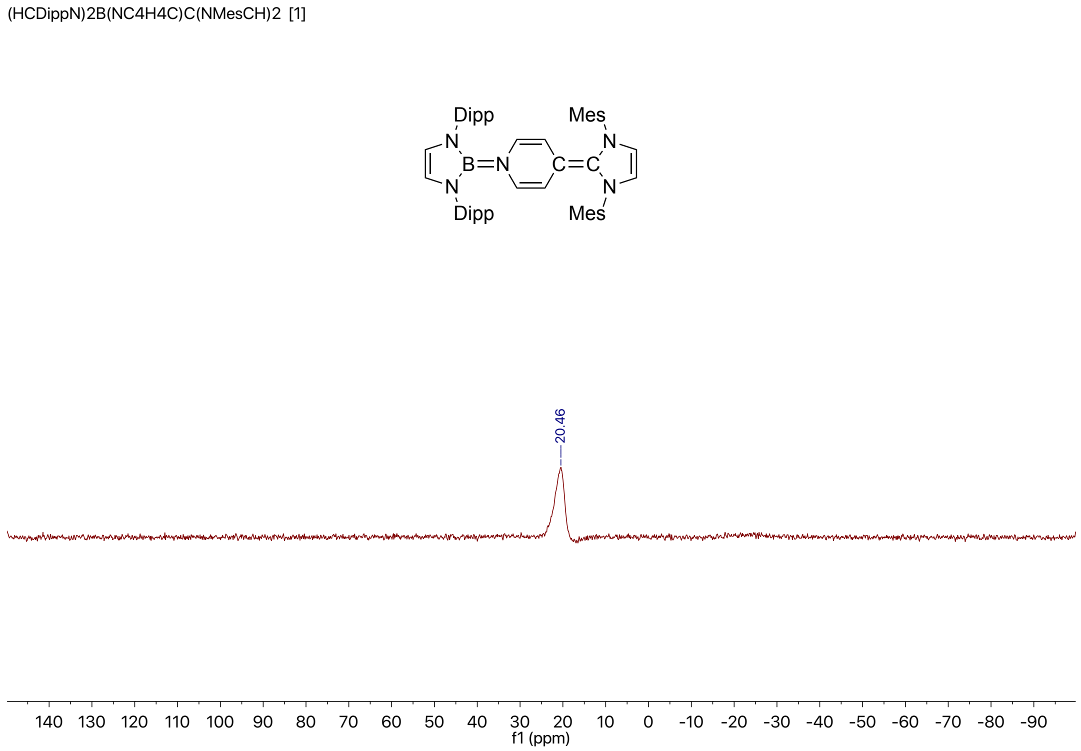


**Supplementary Figure 9.** $^{11}B\{^{1}H\}$ NMR spectrum of **2** (128 MHz, $C_6D_6$).

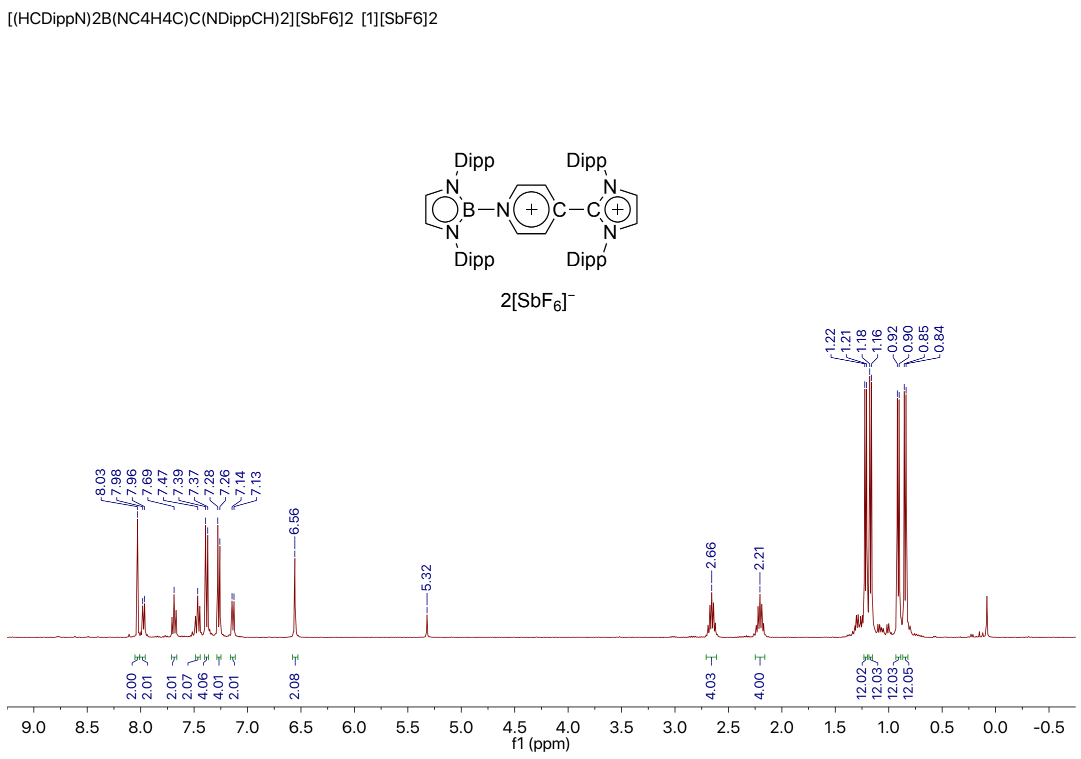


**Supplementary Figure 10.** $^{1}$H NMR spectrum of **1[SbF$_6$]$_2$** (400 MHz, $CD_2Cl_2$).

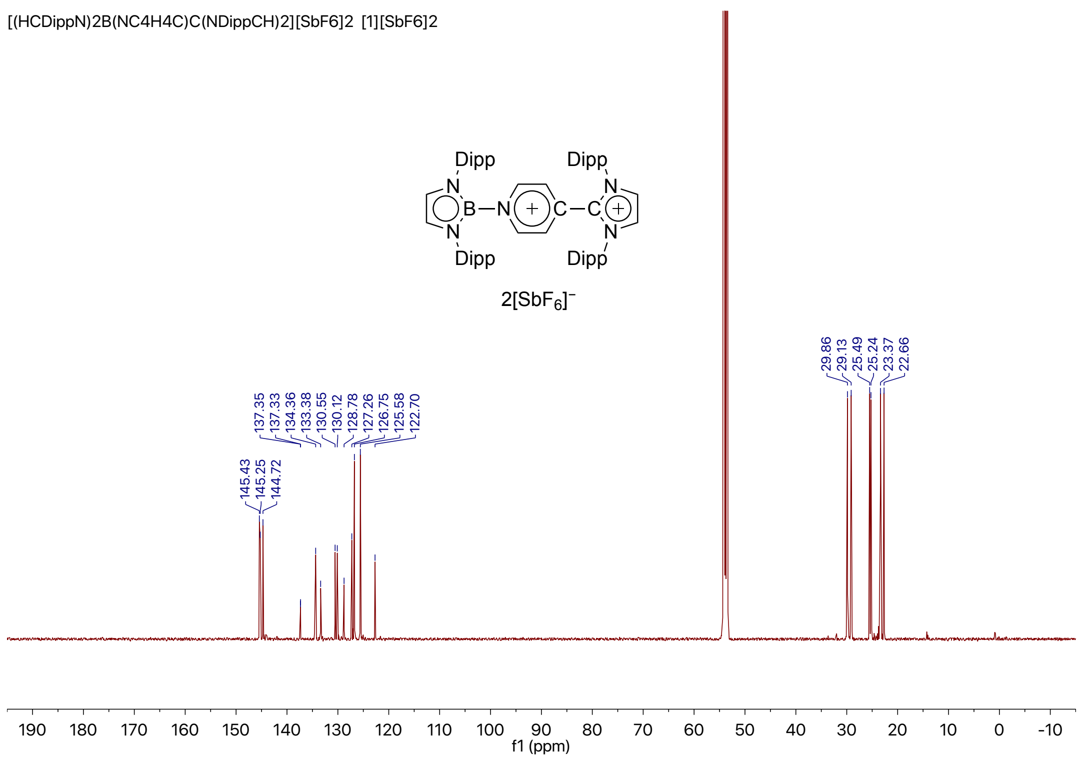


**Supplementary Figure 11.** $^{13}$C{$^{1}$H} NMR spectrum of **1[SbF$_6$]$_2$** (126 MHz, $CD_2Cl_2$).

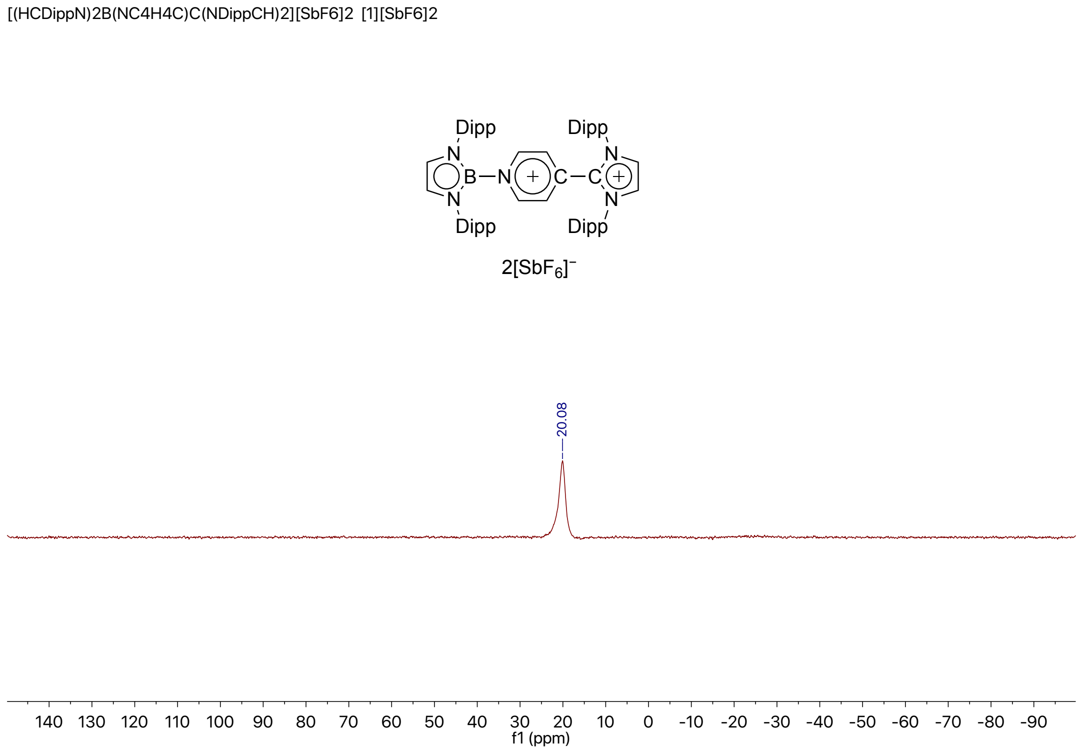


**Supplementary Figure 12.** $^{11}B\{^{1}H\}$ NMR spectrum of **1[SbF$_6$]$_2$** (126 MHz, $CD_2Cl_2$).

## 4. Cyclic voltammetry studies

All electrochemical experiments were measured as freshly distilled dichloromethane solutions in the argon atmosphere glove box using the Gamry Instruments Reference 600 potentiostat. As the electrolyte 0.1 M $^{n}Bu_4N^{+}$ $PF_6^{-}$ was used, the working, counter and reference electrodes were platinum and the voltammograms were internally referenced to decamethylferrocene. The scan rate is 100 mV $s^{-1}$ and the voltammogram shown is from the second cycle of the measurements.

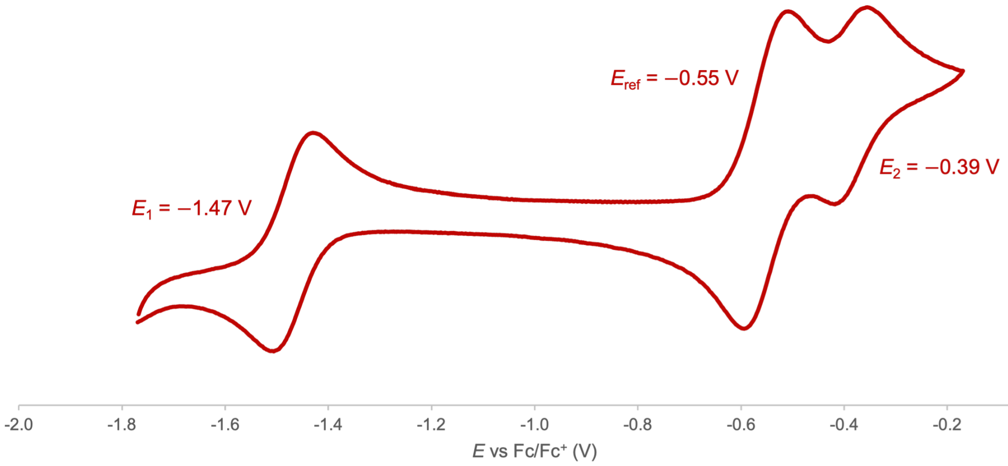


**Supplementary Figure 13.** Cyclic voltammagram of **1** in $CH_2Cl_2$, referenced internally with decamethylferrocene against $Fc/Fc^{+}$.

## 5. EPR studies

Continuous wave electron paramagnetic resonance (CW-EPR) was collected in the Centre for Advanced Electron Spin Resonance (CAESR) in the Department of Chemistry of the University of Oxford. The spectrometer was a Bruker BioSpin EMXmicro with a 6-inch magnet and ER4123-SHQE-W1 resonator at room temperature. Samples were held in 3.8 mm OD x 2.8 mm ID clear fused quartz tubes filled to 4 cm height with J. Young stable more than one day from glovebox transfer to measurement. Alternatively, samples were in filled in 1.6 mm OD x 1.2 mm ID x 10 cm tubes to 4 cm height and these were dropped into the J. Young tubes. EPR Spin quantitation used manufacturer hardware calibration that was previously found to be accurate within 10% of sample preparation. EPR simulations used the EasySpin package of routines written in MatLAB (The MathWorks, Natick, N.J.) program development environment.[2] DFT calculations were performed with the ORCA ab initio, DFT, and semi-empirical SCF-MO package[3,4] with geometry optimization starting from single crystal coordinates and subsequent EPR-NMR properties calculations. The functional was B3LYP and the basis set was EPR-II including a Def2 auxiliary basis.[5,6]

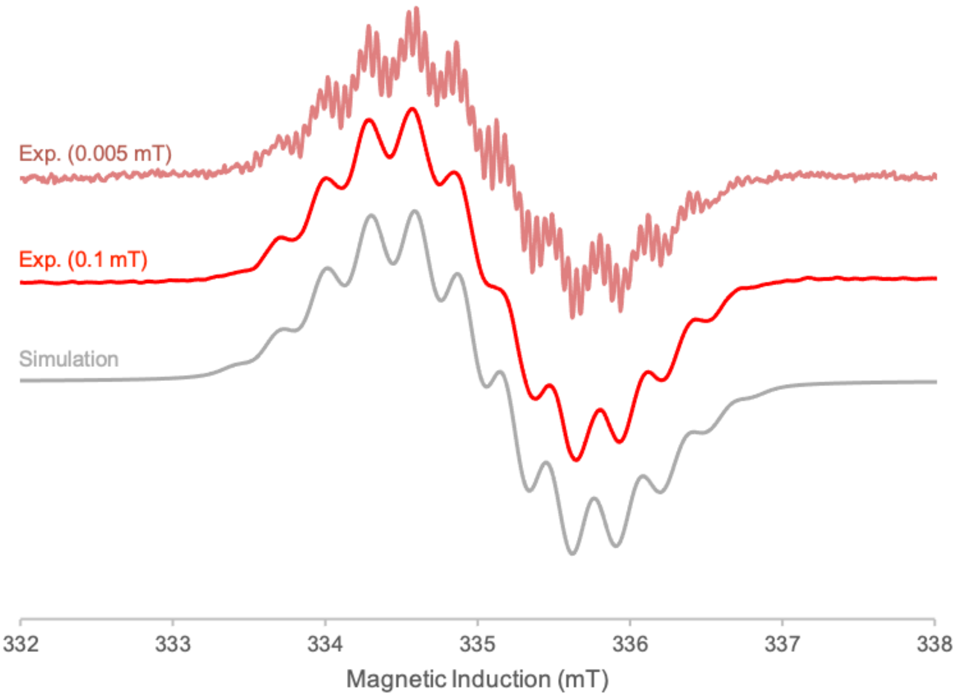


**Supplementary Figure 14.** X-band field-modulated CW-EPR absorption spectra (dχ″/dB) of **1[SbF$_6$]** (modulations 0.005 mT in salmon and 0.1 mT in red) in $CH_2Cl_2$ collected at 295 K and simulated EPR spectrum in grey**.** For the experiment, top spectrum of 0.005 mT modulation amplitude, the microwave frequency was 9.3903 GHz, a microwave power of 10 microWatts, a sweep rate was 7 mT per 600 seconds as the average of 20 sweeps with a time constant of 10.24 msec for 14000 points. For the experiment, middle spectrum of 0.1 mT modulation amplitude, the microwave frequency was 9.3903 GHz, the microwave power of 10 microWatts, the sweep rate was 7 mT per 120 seconds for one sweep with a time constant of 81.92 msec for 700 points. The simulation of **1**$^{·+}$, at bottom in grey, involves instrument parameter values and the largest isotropic values from DFT results (in MHz): H(C3) −8.8197, N(2) 6.2299, N(3) 6.514, H(C4) −8.9517, N(1) 8.8132, B(1) −9.2702, according to Scheme 3 labeling. Following the field correction of 0.085 mT, the calculated isotropic g-value was shifted by −0.001 to 2.0021 with a pseudo-Voigt lineshape of 0.175 mT used.

## 6. UV-vis studies

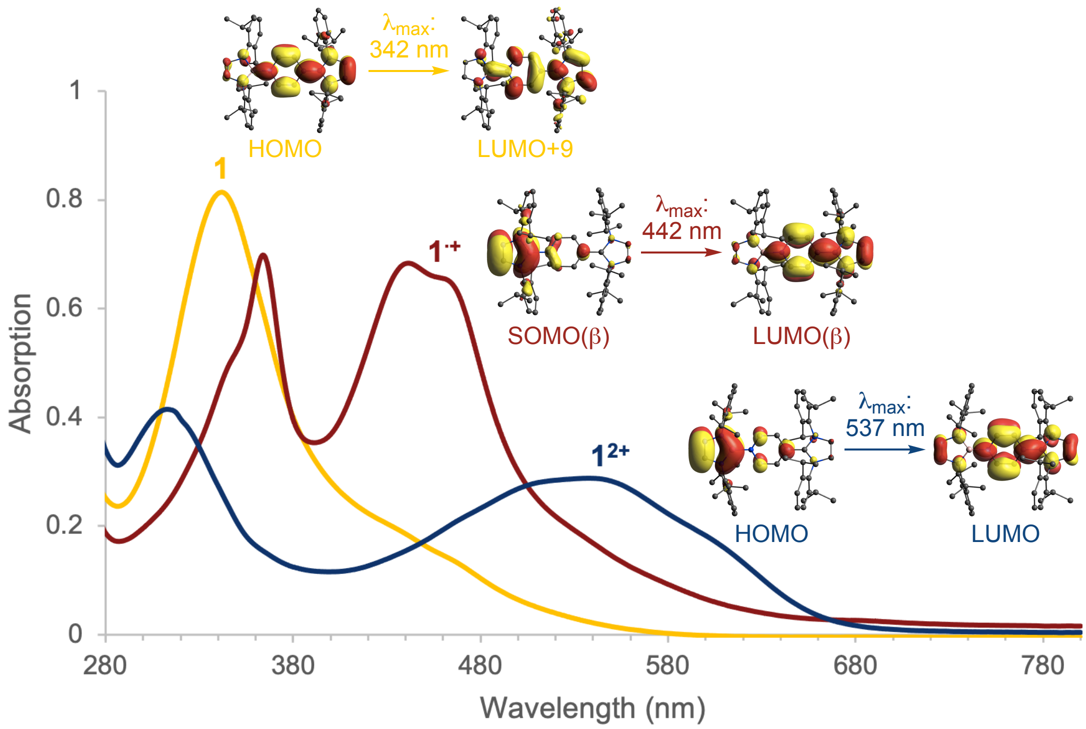


**Supplementary Figure 15.** UV-vis spectra of **1** (0.05 mM ), **1[SbF$_6$]** (0.05 mM ) and **1[SbF$_6$]$_2$** (0.05 mM ) in $CH_2Cl_2$.

**Supplementary Table 1**. TD-DFT data for **1**, **1**$^{\cdot+}$ and **1**$^{2+}$.

| | λ (nm) | f (oscillator strength) | Assignment |
|---|---|---|---|
| **1** | 352 | 0.0452 | 232 -> 242 0.44348 contribution: 39.3%<br>232 -> 243 -0.40356 contribution: 32.6%<br>232 -> 244 0.36003 contribution: 25.9% |
| | 515 | 0.0315 | 232 -> 234 0.14514 contribution: 4.2%<br>232 -> 235 0.66635 contribution: 88.8%<br>232 -> 236 0.13973 contribution: 3.9% |
| **1**$^{\cdot+}$ | 413 | 0.143 | 232A -> 234A 0.59616 contribution: 35.5%<br>232A -> 235A 0.17130 contribution: 2.9%<br>232A -> 238A 0.70345 contribution: 49.5%<br>224B -> 232B -0.25282 contribution: 6.4%<br>231B -> 232B -0.10225 contribution: 10.5% |
| | 518 | 0.0858 | 232A -> 238A 0.11270 contribution: 1.3%<br>231B -> 232B 0.98146 contribution: 96.3% |
| **1**$^{2+}$ | 381 | 0.0054 | 223 -> 232 0.69840 contribution: 97.6% |
| | 580 | 0.257 | 231 -> 232 0.70179 contribution: 98.5% |

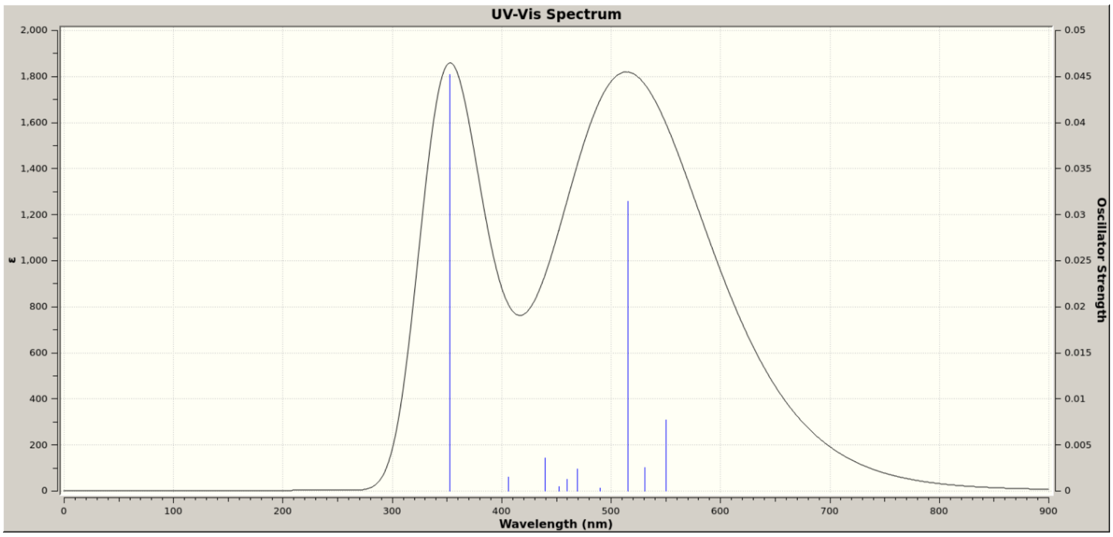


**Supplementary Figure 16.** TD-DFT simulated spectrum of **1.**

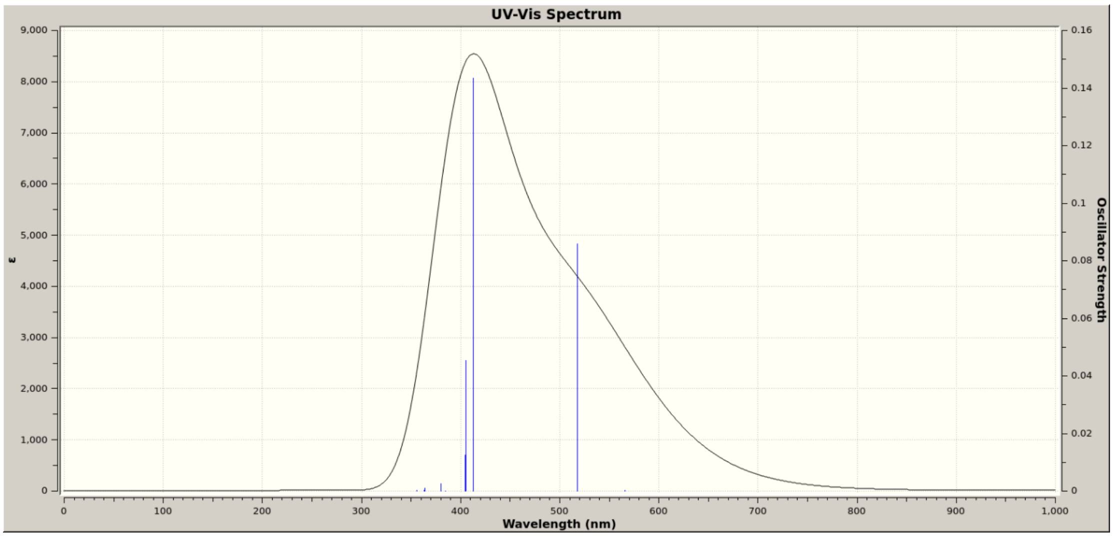


**Supplementary Figure 17.** TD-DFT simulated spectrum of $\mathbf{1}^{\bullet+}$.

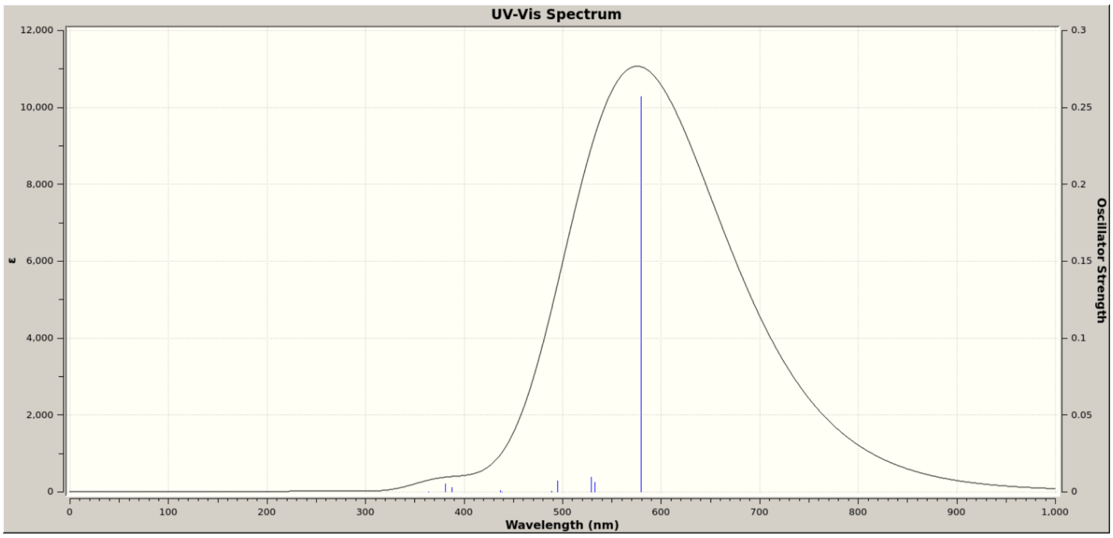


**Supplementary Figure 18.** TD-DFT simulated spectrum of $\mathbf{1}^{2+}$.

## 7. X-ray crystallographic studies

Single-crystal X-ray diffraction data for **2, 1[SbF$_6$]** and **1[SbF$_6$]$_2$** were collected at 150 K on Oxford Diffraction/Agilent SuperNova diffractometers with Cu-*Kα* ($\lambda$ = 1.54184 Å) radiation equipped with nitrogen gas Oxford Cryosystems Cryostream unit.[7] Raw frame data were reduced using CrysAlisPro.[8] The structures were solved using SHELXT[9] and refined to convergence on $F^2$ and against all independent reflections by full-matrix least-squares using SHELXL[10] in combination with the X-seed[11] or Olex2 programs.[12] Distances and angles were calculated using the full covariance matrix. Restraints were used to maintain sensible geometries for the disordered groups and approximate the displacement parameters to typical values. Selected crystallographic data are summarized in the Supplementary Table 2 and full details are given in the supplementary deposited CIF files (2063252-2063254). These data can be obtained free of charge from the Cambridge Crystallographic Data Centre via http://www.ccdc.cam.ac.uk/data_request/cif.

| **Supplementary Table 2**. Selected crystallographic and refinement data for **2**, **1[SbF$_6$]** and **1[SbF$_6$]$_2$**. | | | |
|---|---|---|---|
| | **2** | **1[SbF$_6$]** | **1[SbF$_6$]$_2$** |
| Formula | $C_{52}$ $H_{64}$ B $N_5$ | $C_{58}$ $H_{76}$ B $F_6$ $N_5$ Sb | $C_{60}$ $H_{80}$ B $Cl_4$ $F_{12}$ $N_5$ $Sb_2$ |
| Fw | 769.89 | 1089.79 | 1495.40 |
| Cryst. System | Triclinic | Triclinic | Triclinic |
| Space Group | P –1 | P –1 | P –1 |
| Wavelength/Å | 1.54184 | 1.54184 | 1.54184 |
| *a*/Å | 12.0050(5) | 10.5062(3) | 11.7766(2) |
| *b*/Å | 17.1016(7) | 13.8854(3) | 12.6941(2) |
| *c*/Å | 22.3322(12) | 22.0812(6) | 13.0385(3) |
| α/° | 93.809(4) | 73.714(2) | 72.300(2) |
| β/° | 92.653(4) | 76.539(2) | 86.382(2) |
| γ/° | 97.847(3) | 70.779(2) | 66.833(2) |
| Volume/Å$^3$ | 4524.8(4) | 2884.38(14) | 1703.61(6) |
| *Z* | 4 | 2 | 1 |
| Temp./K | 150(2) | 150(2) | 150(2) |
| Refls. Collect. | 16119 | 11931 | 6948 |
| $2\theta_{max}$ | 133.996 | 152.63 | 152.362 |
| Goodness of fit | 0.989 | 1.037 | 1.051 |
| $R[F^2>2\sigma]$, F | 0.0598 | 0.0467 | 0.0343 |
| $R_w$ (all data), $F^2$ | 0.1601 | 0.1167 | 0.0918 |
| CCDC ref | 2063252 | 20632053 | 20632054 |

## 8. Computational studies

The geometry optimizations for **1**, **1**$^{\cdot+}$, **1**$^{2+}$ and **2** were performed with the Gaussian16 (Revision C.01) programme[13] using the PBE1PBE hybrid exchange functional[14-16] and Def-TZVP basis set.[17,18] In addition, Grimme's empirical dispersion correction with Becke-Johnson damping (GD3BJ)[19] was used as well as an ultrafine integration grid. Full analytical frequency calculations were performed for the optimized structures to ensure the nature of the stationary points found (minima, no imaginary frequencies). The NBO analyses were performed using the program NBO 7.0.5.[20] TD-DFT and NICS calculations were performed as single-point calculations in the gas phase for the optimized systems **1**, **1**$^{\cdot+}$ and **1**$^{2+}$ as implemented in Gaussian16.

### NICS(0) and NICS(1) Calculations

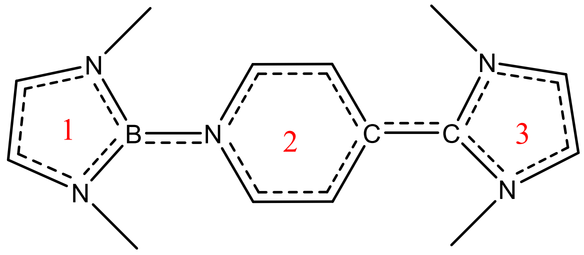


**Supplementary Table 3**. NICS(0) and NICS(1) data for **1**, **1**$^{\cdot+}$ and **1**$^{2+}$.

| | **1** | **1**$^{\cdot+}$ | **1**$^{2+}$ |
|---|---|---|---|
| Ring 1 NICS(0) | -8.1382 | -9.0253 | -9.001 |
| Ring 1 NICS(1) | -4.1778 | -5.0367 | -7.111 |
| Ring 2 NICS(0) | 6.7618 | 1.522 | -7.5506 |
| Ring 2 NICS(1) | 4.137 | -0.8654 | -8.9863 |
| Ring 3 NICS(0) | -7.0003 | -10.135 | -12.3617 |
| Ring 3 NICS(1) | -2.4116 | -5.3367 | -10.3018 |

### NRT calculations

In all three structures, the Dipp-substituents were replaced with protons for computational efficiency. The proton positions for the simplified model compounds were optimized by freezing all heavy atoms and the NRT analysis was then performed.

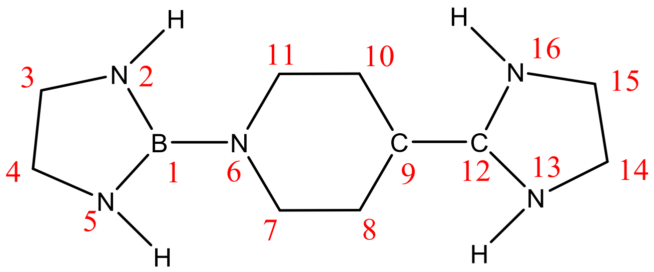


**Supplementary Table 4**. Natural Bond Orders for **1**, **1**$^{\cdot+}$ and **1**$^{2+}$.

| Bond | **1** | **1**$^{\cdot+}$ | **1**$^{2+}$ |
|---|---|---|---|
| B1-N6 | 1.0879 | 1.0029 | 1.0075 |
| B1-N2 | 1.3596 | 1.3831 | 1.3439 |

| | | | |
|---|---|---|---|
| N2-C3 | 1.2521 | 1.2526 | 1.3418 |
| C3-C4 | 1.6148 | 1.6086 | 1.5482 |
| C4-N5 | 1.2483 | 1.2565 | 1.3418 |
| N5-B1 | 1.3566 | 1.4135 | 1.3439 |
| N6-C7 | 1.1628 | 1.3040 | 1.3928 |
| C7-C8 | 1.7517 | 1.6367 | 1.4788 |
| C8-C9 | 1.1413 | 1.2922 | 1.4544 |
| C9-C10 | 1.1444 | 1.2869 | 1.4544 |
| C10-C11 | 1.7537 | 1.6527 | 1.4788 |
| C11-N6 | 1.1610 | 1.2948 | 1.3928 |
| C9-C12 | 1.4440 | 1.3029 | 1.0356 |
| C12-N13 | 1.1320 | 1.2261 | 1.3777 |
| N13-C14 | 1.2211 | 1.2927 | 1.3466 |
| C14-C15 | 1.6285 | 1.5925 | 1.5678 |
| C15-N16 | 1.2442 | 1.2901 | 1.3466 |
| N16-C12 | 1.1267 | 1.2230 | 1.3777 |

**Compound 1:**

3-Center, 4-Electron A:-B-:C Hyperbonds (A-B :C <=> A: B-C)

Hyperbond A:-B-:C %A-B/%B-C

1. N6:- B1-: N5 45.7/54.3
2. N6:- B1-: N2 45.6/54.4

Resonance structures: Over 125 resonance structures were found in total, which is in agreement with highly delocalized system, parent structure has a weight of only 5.92%. The 20 highest contributions total to 44.46%, of which ca. 54% have C9-C12 double bond and either C7-C8 or C10-C11 double bond and ca. 39% feature structures that exhibit 3 C-C/C-N double bonds in ring 2.

**Supplementary Table 5**. Natural Atomic Valencies and Charges for **1**.

| Atom | Valency | Covalent valency | Ionic valency | Formal charge |
|---|---|---|---|---|
| B1 | 3.8041 | 1.6819 | 2.1223 | -1.1331 |
| N2 | 3.6005 | 2.0539 | 1.5466 | 0.6867 |
| C3 | 3.8063 | 3.2182 | 0.5882 | -0.1937 |
| C4 | 3.8066 | 3.2200 | 0.5866 | -0.1850 |
| N5 | 3.5939 | 2.0497 | 1.5442 | 0.6760 |
| N6 | 3.4117 | 2.0892 | 1.3224 | 0.5867 |
| C7 | 3.8642 | 3.2671 | 0.5971 | -0.1358 |
| C8 | 3.8478 | 3.5466 | 0.3012 | -0.1276 |
| C9 | 3.8152 | 3.5401 | 0.2751 | -0.0620 |
| C10 | 3.8545 | 3.5490 | 0.3055 | -0.1386 |
| C11 | 3.8627 | 3.2671 | 0.5956 | -0.1304 |
| C12 | 3.7215 | 3.0116 | 0.7099 | -0.2677 |
| N13 | 3.3881 | 2.2675 | 1.1206 | 0.4602 |
| C14 | 3.7960 | 3.2218 | 0.5742 | -0.1837 |
| C15 | 3.8289 | 3.2307 | 0.5982 | -0.1687 |
| N16 | 3.3921 | 2.2755 | 1.1167 | 0.5190 |

Compound $1^{\cdot+}$:

3-Center, 4-Electron A:-B-:C Hyperbonds (A-B :C <=> A: B-C)

α-spin

Hyperbond A:-B-:C   %A-B/%B-C

1. N2:- B1-: N5  50.2/49.8

β-spin

Hyperbond A:-B-:C   %A-B/%B-C

1. N13:-C14-: C15  27.0/73.0
2. C12:- N16-: C15  54.6/45.4

Resonance structures: (α-spin) Over 180 resonance structures were found in total, which is in agreement with highly delocalized system, parent structure has a weight of only 1.73%. The 20 highest contributions total 24.84% of the calculated resonance structures, of which: ca. 4% exhibit a C9-C12 single bond and C7-C8 and C10-C11 double bonds (resembling structure $1^{\cdot+}$**a**). Ca. 66% of structures have a C9-C12 double bond and two C-C double bonds in ring 2 (resembling structure $1^{\cdot+}$**b**). Ca. 30% of the structures feature 3 double bonds in the ring 2 with cationic charge localized on N6 (resembling structure $1^{\cdot+}$**c**). (β-spin) Over 162 resonance structures found in total, which is in agreement with highly delocalized system, parent structure has a weight of only 2.52%. The 20 highest contributions total 43.41% of the calculated resonance structures, of which all feature 3 double bonds in the ring 2 and C9-C12 single bond (resembling structure $1^{\cdot+}$**c**).

**Supplementary Table 6**. Natural Atomic Valencies and Charges for $1^{\cdot+}$

| Atom | Valency | Covalent valency | Ionic valency | Formal charge |
|---|---|---|---|---|
| B1 | 3.8147 | 1.7268 | 2.0879 | -1.1439 |
| N2 | 3.6462 | 2.0818 | 1.5644 | 0.7123 |
| C3 | 3.8156 | 3.1924 | 0.6232 | -0.1808 |
| C4 | 3.8209 | 3.1961 | 0.6248 | -0.1758 |
| N5 | 3.6677 | 2.0688 | 1.5990 | 0.7443 |
| N6 | 3.6343 | 2.3117 | 1.3225 | 0.7619 |
| C7 | 3.9264 | 3.2764 | 0.6500 | -0.0558 |
| C8 | 3.8987 | 3.5310 | 0.3677 | -0.0434 |
| C9 | 3.9034 | 3.5710 | 0.3324 | -0.0374 |
| C10 | 3.8918 | 3.5197 | 0.3721 | -0.0632 |
| C11 | 3.9118 | 3.2494 | 0.6624 | -0.0612 |
| C12 | 3.7921 | 3.0052 | 0.7868 | -0.1956 |
| N13 | 3.5645 | 2.3921 | 1.1724 | 0.6075 |
| C14 | 3.8372 | 3.1958 | 0.6413 | -0.1552 |
| C15 | 3.8304 | 3.1923 | 0.6381 | -0.1572 |
| N16 | 3.5565 | 2.3945 | 1.1621 | 0.5994 |

Compound $1^{2+}$:

3-Center, 4-Electron A:-B-:C Hyperbonds (A-B :C <=> A: B-C)

Hyperbond A:-B-:C   %A-B/%B-C

1. N13:- C14-: C15  24.4/75.6
2. C 12:- N16-: C15  54.4/45.6

Resonance structures: Over 128 resonance structures were found in total. Parent structure has a weight of only 2.48%. 24 biggest contributions total 45.02% of the overall structures, of which: ca 71% exhibit 3 C-C/C-N double bonds in the ring 2 (resembling structure $\mathbf{1^{2+}a}$). Ca. 23% resemble structure $\mathbf{1^{2+}b}$ and ca. 6% of resonance structures have C9-C12 double bond and either C7-C8 or C10-C11 double bond (resembling structure $\mathbf{1^{2+}c}$).

**Supplementary Table 7**. Natural Atomic Valencies and Charges for $\mathbf{1^{2+}}$

| Atom | Valency | Covalent valency | Ionic valency | Formal charge |
|---|---|---|---|---|
| B1 | 3.7751 | 1.7370 | 2.0381 | -1.0661 |
| N2 | 3.6791 | 2.1051 | 1.5740 | 0.7849 |
| C3 | 3.8274 | 3.1369 | 0.6905 | -0.1708 |
| C4 | 3.8274 | 3.1369 | 0.6905 | -0.1708 |
| N5 | 3.6791 | 2.1051 | 1.5740 | 0.7849 |
| N6 | 3.7931 | 2.3914 | 1.4017 | 0.8602 |
| C7 | 3.9138 | 3.1961 | 0.7177 | -0.0570 |
| C8 | 3.9559 | 3.5856 | 0.3703 | -0.0264 |
| C9 | 3.9443 | 3.7775 | 0.1668 | -0.0113 |
| C10 | 3.9559 | 3.5856 | 0.3703 | -0.0264 |
| C11 | 3.9138 | 3.1961 | 0.7177 | -0.0570 |
| C12 | 3.7909 | 2.9989 | 0.7920 | -0.1866 |
| N13 | 3.7052 | 2.4466 | 1.2586 | 0.7701 |
| C14 | 3.8757 | 3.1723 | 0.7034 | -0.1146 |
| C15 | 3.8757 | 3.1723 | 0.7034 | -0.1146 |
| N16 | 3.7052 | 2.4466 | 1.2586 | 0.7701 |

**Frontier orbital energies**

**1**

HOMO: -0.12611 au = -3.432 eV = -331.1 kJ/mol

LUMO: -0.01229 au = -0.3344 eV = -32.3 kJ/mol

HOMO-LUMO gap: 3.097 eV = 298.8 kJ/mol

$\mathbf{1^{\cdot+}}$

SOMO (alpha): -0.25129 au = -6.837 eV = -659.76 kJ/mol

LUMO (alpha): -0.12027 au = -3.273 eV = -315.77 kJ/mol

LUMO (beta): -0.18070 au = -4.9171 eV = -474.43 kJ/mol

SOMO-LUMO (alpha) gap: 0.13102 au = 3.564 eV = 343.99 kJ/mol

$\mathbf{1^{2+}}$

HOMO: -0.40190 au = -10.936 eV = -1055.19 kJ/mol

LUMO: -0.30782 au = -8.376 eV = -808.18 kJ/mol

HOMO-LUMO gap: 0.09408 au = 2.56 eV = 247.0 kJ/mol

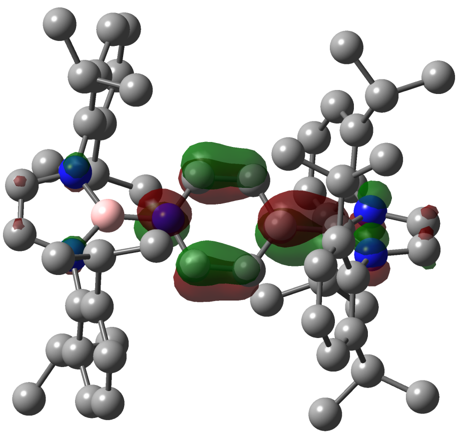

**Supplementary Figure 19.** DFT calculated LUMO for $\mathbf{1}^{2+}$. Isovalue is set at 0.005 a.u.

**Xyz-coordinates of optimized structures:**

**$(HCDippN)_2B(NC_5H_4C)C(NDippCH)_2$ [1]**

140

| | | | |
|---|---|---|---|
| C | 2.380308 | -5.329636 | -0.674276 |
| C | 2.709901 | -4.705896 | 0.514611 |
| C | 3.105465 | -3.371589 | 0.534384 |
| C | 3.153111 | -2.675536 | -0.679504 |
| C | 2.829823 | -3.287505 | -1.899835 |
| C | 2.444995 | -4.624784 | -1.865561 |
| N | 3.561672 | -1.311985 | -0.665166 |
| C | 2.800163 | -0.217012 | -0.214210 |
| N | 3.753563 | 0.804148 | -0.058743 |
| C | 5.031391 | 0.299449 | -0.290779 |
| C | 4.916693 | -0.983701 | -0.641087 |
| C | 3.545205 | 2.175792 | 0.239343 |
| C | 3.927934 | 2.649281 | 1.501510 |
| C | 3.744112 | 4.001900 | 1.771610 |
| C | 3.182321 | 4.844313 | 0.827092 |
| C | 2.802987 | 4.352536 | -0.411506 |
| C | 2.988159 | 3.013841 | -0.737267 |
| C | 1.452747 | -0.161440 | 0.029349 |
| C | 0.533552 | -1.273312 | -0.175150 |
| C | -0.791484 | -1.156105 | -0.021229 |
| N | -1.421755 | 0.041901 | 0.332640 |
| C | -0.559037 | 1.092496 | 0.661754 |
| C | 0.772271 | 1.021552 | 0.542811 |
| B | -2.848594 | 0.209430 | 0.341257 |
| N | -3.569093 | 1.441452 | 0.143592 |
| C | -3.081622 | 2.651107 | -0.417491 |
| C | -2.986331 | 3.797482 | 0.381661 |
| C | -2.494225 | 4.964762 | -0.193583 |
| C | -2.098498 | 4.993046 | -1.519852 |
| C | -2.194117 | 3.849024 | -2.293634 |
| C | -2.688420 | 2.661588 | -1.763849 |
| C | -3.395659 | 3.777104 | 1.836890 |
| C | -4.673153 | 4.584834 | 2.057574 |
| C | -2.784579 | 1.431147 | -2.638114 |
| C | -3.720940 | 1.660518 | -3.821566 |
| C | 4.429956 | 1.695587 | 2.565202 |
| C | 2.830070 | -2.581528 | -3.242504 |
| C | 3.434917 | -2.705034 | 1.850849 |
| C | 2.597358 | 2.459833 | -2.088751 |
| H | 3.034023 | 5.893230 | 1.059540 |
| H | 2.070009 | -6.368766 | -0.675948 |
| C | -4.935259 | 1.157019 | 0.208590 |
| C | -5.111260 | -0.156889 | 0.439626 |
| N | -3.868219 | -0.787078 | 0.530179 |
| C | -3.734377 | -2.182698 | 0.740297 |
| C | -3.943423 | -3.055521 | -0.337507 |
| C | -3.756175 | -4.417578 | -0.127180 |
| C | -3.366350 | -4.900395 | 1.110980 |
| C | -3.165648 | -4.023602 | 2.163020 |
| C | -3.349984 | -2.653662 | 2.002435 |
| C | -4.308992 | -2.542579 | -1.713574 |
| C | -3.163816 | -2.757608 | -2.702193 |
| C | -3.111836 | -1.714637 | 3.162671 |
| C | -1.631976 | -1.668384 | 3.539060 |
| C | -3.977302 | -2.078144 | 4.366203 |
| C | -5.603219 | -3.167794 | -2.225618 |
| C | -2.277041 | 4.267781 | 2.752681 |
| C | -1.406625 | 0.967125 | -3.103858 |
| H | -5.676285 | 1.929639 | 0.080460 |
| H | 0.894436 | -2.250993 | -0.451027 |
| H | -6.028030 | -0.708615 | 0.574082 |
| H | -1.443529 | -2.005301 | -0.163518 |
| H | -1.036270 | 1.979787 | 1.050934 |
| H | 1.327837 | 1.886984 | 0.867251 |
| H | -3.403875 | -0.714402 | 2.835554 |
| H | -4.474791 | -1.466900 | -1.631910 |
| H | -3.607887 | 2.737339 | 2.096699 |
| H | -2.857354 | -4.408748 | 3.129008 |
| H | -3.905095 | -5.109529 | -0.949278 |
| H | -2.412436 | 5.863098 | 0.408772 |
| H | -3.216126 | -5.964696 | 1.255427 |
| H | -3.217057 | 0.629971 | -2.035289 |
| H | -1.878280 | 3.876828 | -3.331059 |
| H | 5.896360 | 0.936272 | -0.221829 |
| H | 4.028128 | 4.399224 | 2.738367 |
| H | -5.036795 | -2.098778 | 4.100671 |
| H | -3.839523 | -1.346330 | 5.166556 |
| H | -3.713277 | -3.060018 | 4.768596 |
| H | 5.015412 | 0.916716 | 2.069119 |
| C | 3.247832 | 1.004851 | 3.247485 |
| C | 5.335843 | 2.355651 | 3.596082 |
| H | 5.663910 | -1.710677 | -0.906357 |
| H | 2.655051 | -5.258976 | 1.445929 |
| H | -1.710773 | 5.909343 | -1.951565 |
| H | -0.945846 | 1.707923 | -3.762581 |
| H | -1.489617 | 0.030855 | -3.662059 |
| H | -0.735223 | 0.800993 | -2.259655 |
| H | 2.353370 | 5.021395 | -1.134358 |
| H | 2.182946 | -5.116849 | -2.796459 |
| H | -1.284119 | -2.646211 | 3.884937 |
| H | -1.465407 | -0.948929 | 4.345425 |
| H | -1.015574 | -1.375375 | 2.687637 |

| | | | |
|---|---|---|---|
| H | -1.359066 | 3.693269 | 2.608577 |
| H | -2.575313 | 4.171100 | 3.799899 |
| H | -2.042273 | 5.320173 | 2.572574 |
| H | -5.500357 | -4.247483 | -2.364607 |
| H | -5.879077 | -2.734540 | -3.190616 |
| H | -6.426669 | -2.998389 | -1.527979 |
| H | -2.248069 | -2.267685 | -2.363652 |
| H | -3.424585 | -2.347287 | -3.681866 |
| H | -2.947059 | -3.821809 | -2.830688 |
| C | 1.777671 | -1.479112 | -3.337086 |
| H | 2.543452 | -3.356048 | -3.962857 |
| C | 4.201602 | -2.069627 | -3.677269 |
| H | 3.712747 | -1.670721 | 1.643928 |
| C | 4.628357 | -3.373508 | 2.528037 |
| C | 2.215187 | -2.674590 | 2.768832 |
| H | -4.715741 | 1.961245 | -3.485208 |
| H | -3.820983 | 0.743405 | -4.408706 |
| H | -3.340050 | 2.440750 | -4.486453 |
| H | -4.517681 | 5.638315 | 1.807789 |
| H | -4.988996 | 4.529388 | 3.102851 |
| H | -5.490176 | 4.213174 | 1.435133 |
| C | 3.828743 | 2.243333 | -2.969325 |
| C | 1.567969 | 3.311480 | -2.816160 |
| H | 2.137200 | 1.482797 | -1.909552 |
| H | 4.882267 | -2.847495 | 3.452224 |
| H | 4.410772 | -4.413540 | 2.786348 |
| H | 5.507356 | -3.365545 | 1.879201 |
| H | 2.450043 | -2.143596 | 3.694789 |
| H | 1.377319 | -2.167837 | 2.287045 |
| H | 1.893056 | -3.685248 | 3.035235 |
| H | 4.169330 | -1.781086 | -4.731421 |
| H | 4.503728 | -1.192844 | -3.103958 |
| H | 4.969424 | -2.839110 | -3.562789 |
| H | 1.753428 | -1.075342 | -4.353207 |
| H | 0.781537 | -1.854533 | -3.095341 |
| H | 1.993484 | -0.657966 | -2.652584 |
| H | 3.535734 | 1.798448 | -3.924257 |
| H | 4.324921 | 3.196020 | -3.176290 |
| H | 4.552835 | 1.576635 | -2.498644 |
| H | 1.250346 | 2.798576 | -3.726722 |
| H | 0.682096 | 3.485090 | -2.202079 |
| H | 1.979488 | 4.279300 | -3.118065 |
| H | 5.764073 | 1.594596 | 4.252658 |
| H | 6.158276 | 2.898948 | 3.124415 |
| H | 4.786855 | 3.055461 | 4.232005 |
| H | 3.604865 | 0.293497 | 3.997675 |
| H | 2.613120 | 1.739609 | 3.751054 |
| H | 2.628069 | 0.462426 | 2.531549 |

**$[(HCDippN)_2B(NC_4H_4C)C(NDippCH)_2]^{\cdot+}$ $[1]^{\cdot+}$**

140

| | | | |
|---|---|---|---|
| N | 3.733378 | -1.064209 | 0.394609 |
| N | 3.709422 | 1.110691 | -0.274909 |
| C | 3.393586 | -2.371266 | 0.841030 |
| C | 3.339868 | 2.393216 | -0.766958 |
| C | 3.503602 | -3.444341 | -0.054143 |
| C | 5.043360 | -0.605533 | 0.262109 |
| H | 5.885308 | -1.243967 | 0.475633 |
| C | 2.901693 | -2.537715 | 2.142488 |
| C | 3.417328 | 3.500188 | 0.089098 |
| C | 2.994176 | 4.732914 | -0.397964 |
| H | 3.049287 | 5.605995 | 0.242828 |
| C | 0.674501 | 1.134557 | 0.321095 |
| H | 1.247459 | 2.021066 | 0.549692 |
| C | 5.029209 | 0.682738 | -0.137395 |
| H | 5.857156 | 1.340636 | -0.346299 |
| C | 3.926281 | 3.379852 | 1.508809 |
| H | 4.129227 | 2.324115 | 1.701735 |
| C | 3.109027 | -4.703892 | 0.385776 |
| H | 3.188832 | -5.551730 | -0.285671 |
| C | 2.852929 | 2.501170 | -2.076777 |
| C | 2.788537 | -1.386433 | 3.117041 |
| H | 3.170631 | -0.491356 | 2.620515 |
| C | 0.688491 | -1.140426 | -0.234133 |
| H | 1.272142 | -2.029177 | -0.424320 |
| C | 2.509564 | -3.815062 | 2.531197 |
| H | 2.124928 | -3.971222 | 3.533066 |
| C | 4.012262 | -3.260085 | -1.467397 |
| H | 4.222273 | -2.198108 | -1.611315 |
| C | 2.430362 | 3.753152 | -2.512997 |
| H | 2.048226 | 3.863525 | -3.521939 |
| C | 2.781060 | 1.315455 | -3.013263 |
| H | 3.163313 | 0.443313 | -2.477528 |
| C | 2.499946 | 4.860283 | -1.685053 |
| H | 2.173131 | 5.828810 | -2.046587 |
| C | 2.612123 | -4.889704 | 1.664791 |
| H | 2.308286 | -5.878522 | 1.989507 |
| C | 3.651005 | -1.623128 | 4.354574 |
| H | 3.309518 | -2.495496 | 4.917625 |
| H | 3.605119 | -0.758735 | 5.021672 |
| H | 4.695306 | -1.788989 | 4.082267 |
| C | 5.236979 | 4.143267 | 1.687186 |
| H | 5.997416 | 3.797579 | 0.983581 |
| H | 5.624075 | 4.009089 | 2.700106 |
| H | 5.095230 | 5.214509 | 1.521934 |

| | | | |
|---|---|---|---|
| C | 2.883059 | 3.841342 | 2.524648 |
| H | 2.647401 | 4.901601 | 2.404204 |
| H | 3.256730 | 3.699488 | 3.541600 |
| H | 1.949662 | 3.279946 | 2.429515 |
| C | 1.334440 | -1.121234 | 3.503587 |
| H | 0.713561 | -0.936949 | 2.624286 |
| H | 1.266239 | -0.248522 | 4.158233 |
| H | 0.906746 | -1.972786 | 4.039513 |
| C | 5.317326 | -4.022161 | -1.686109 |
| H | 6.080500 | -3.717081 | -0.966853 |
| H | 5.703964 | -3.838721 | -2.691444 |
| H | 5.170016 | -5.099687 | -1.576207 |
| C | 2.962483 | -3.665100 | -2.500784 |
| H | 2.712461 | -4.726088 | -2.422659 |
| H | 3.337731 | -3.486687 | -3.511475 |
| H | 2.037180 | -3.094850 | -2.382017 |
| C | 3.673067 | 1.524174 | -4.234920 |
| H | 3.335313 | 2.374127 | -4.833440 |
| H | 3.655815 | 0.638617 | -4.875160 |
| H | 4.707553 | 1.712614 | -3.940746 |
| C | 1.342603 | 1.013502 | -3.428708 |
| H | 0.698942 | 0.856363 | -2.560471 |
| H | 1.305839 | 0.113826 | -4.048408 |
| H | 0.918663 | 1.835325 | -4.011885 |
| N | -3.704933 | 1.063144 | 0.176171 |
| N | -3.695536 | -1.028763 | -0.416201 |
| C | -1.448979 | -0.001047 | -0.015871 |
| C | -3.369356 | 2.367602 | 0.661499 |
| C | -3.341875 | -2.372346 | -0.760344 |
| C | -3.294066 | 2.555528 | 2.044801 |
| C | -5.016007 | 0.679993 | -0.012849 |
| H | -5.823345 | 1.374703 | 0.141251 |
| C | -3.139630 | 3.385763 | -0.267872 |
| C | -3.128109 | -2.677537 | -2.107123 |
| C | -2.775930 | -3.988456 | -2.411927 |
| H | -2.595069 | -4.265241 | -3.444199 |
| C | -0.663062 | -1.168754 | -0.274189 |
| H | -1.116440 | -2.115709 | -0.512873 |
| C | -5.010201 | -0.614662 | -0.379028 |
| H | -5.810947 | -1.292039 | -0.620169 |
| C | -3.226101 | -1.635890 | -3.198522 |
| H | -3.559095 | -0.699506 | -2.743791 |
| C | -2.863276 | 0.007779 | -0.074314 |
| C | -2.955373 | 3.828076 | 2.493332 |
| H | -2.878253 | 4.014867 | 3.558156 |
| C | -3.234051 | -3.312268 | 0.269007 |
| C | -3.201475 | 3.148622 | -1.759669 |
| H | -3.411761 | 2.088688 | -1.924331 |
| C | -0.676977 | 1.161556 | 0.300013 |
| H | -1.141554 | 2.104395 | 0.532638 |
| C | -2.811366 | 4.640632 | 0.234519 |
| H | -2.621446 | 5.456663 | -0.453111 |
| C | -3.521119 | 1.428876 | 3.027991 |
| H | -3.895594 | 0.565324 | 2.472416 |
| C | -2.879842 | -4.608294 | -0.090864 |
| H | -2.779284 | -5.365817 | 0.677855 |
| C | -3.448086 | -2.951993 | 1.721883 |
| H | -3.728270 | -1.896797 | 1.771350 |
| C | -2.650066 | -4.942206 | -1.415472 |
| H | -2.372651 | -5.957699 | -1.674275 |
| C | -2.715794 | 4.858469 | 1.599082 |
| H | -2.454079 | 5.842876 | 1.970058 |
| C | -4.337111 | 3.948503 | -2.394149 |
| H | -4.176125 | 5.022956 | -2.276435 |
| H | -4.400722 | 3.736637 | -3.463946 |
| H | -5.300635 | 3.705145 | -1.940542 |
| C | -4.260024 | -2.026747 | -4.251197 |
| H | -5.241877 | -2.196673 | -3.803834 |
| H | -4.357725 | -1.235487 | -4.997907 |
| H | -3.969797 | -2.940561 | -4.775129 |
| C | -1.859278 | -1.376838 | -3.830830 |
| H | -1.479895 | -2.272954 | -4.328560 |
| H | -1.932166 | -0.585147 | -4.580372 |
| H | -1.124910 | -1.070639 | -3.083217 |
| C | -1.863207 | 3.458324 | -2.429037 |
| H | -1.047868 | 2.881719 | -1.987809 |
| H | -1.911644 | 3.217861 | -3.493821 |
| H | -1.609590 | 4.517515 | -2.340626 |
| C | -4.577796 | 1.791064 | 4.067730 |
| H | -5.517781 | 2.086499 | 3.596687 |
| H | -4.775810 | 0.935364 | 4.717154 |
| H | -4.248812 | 2.615770 | 4.704218 |
| C | -2.208918 | 1.013170 | 3.692823 |
| H | -1.790670 | 1.835323 | 4.279302 |
| H | -2.376343 | 0.171391 | 4.369174 |
| H | -1.462039 | 0.713269 | 2.955208 |
| C | -4.593273 | -3.758445 | 2.329132 |
| H | -4.365137 | -4.826969 | 2.339230 |
| H | -4.769723 | -3.448124 | 3.361587 |
| H | -5.520529 | -3.621073 | 1.768416 |
| C | -2.160775 | -3.121274 | 2.527776 |
| H | -1.344898 | -2.526568 | 2.112613 |
| H | -2.319031 | -2.806140 | 3.562018 |
| H | -1.838694 | -4.165472 | 2.543710 |
| N | 1.399411 | -0.000335 | 0.051393 |
| B | 2.868075 | 0.012895 | 0.058488 |

**[(HCDippN)$_2$B(NC$_4$H$_4$C)C(NDippCH)$_2$]$^{2+}$ [1]$^{2+}$**

140

| | | | |
|---|---|---|---|
| N | -3.713794 | -1.018168 | -0.370100 |
| C | -3.294275 | -3.397318 | -0.074237 |
| C | -5.024704 | -0.636989 | -0.237664 |
| H | -5.832418 | -1.298826 | -0.500578 |
| C | 2.940049 | 4.124889 | -2.280776 |
| H | 2.915651 | 4.448314 | -3.314041 |
| N | -3.714215 | 1.016679 | 0.371177 |
| C | 3.662996 | -1.830436 | 3.072630 |
| H | 4.336911 | -1.082644 | 2.646494 |
| C | 0.621809 | 1.094108 | -0.404768 |
| H | 1.206534 | 1.944279 | -0.727194 |
| C | 2.942423 | -4.124837 | 2.279823 |
| H | 2.918553 | -4.448772 | 3.312936 |
| C | -2.337413 | -3.516689 | -2.709315 |
| H | -1.971940 | -3.583054 | -3.727246 |
| N | 3.663424 | -1.100730 | 0.291556 |
| C | 1.735139 | 3.084948 | 2.541861 |
| H | 1.400786 | 4.125104 | 2.560058 |
| H | 1.786721 | 2.734943 | 3.575575 |
| H | 0.965500 | 2.503239 | 2.026923 |
| C | -2.358957 | 4.650132 | 1.914903 |
| H | -2.007613 | 5.593863 | 2.315750 |
| C | 3.352320 | -2.455704 | 0.624096 |
| C | 3.099855 | 2.951714 | 1.867696 |
| H | 3.395218 | 1.900636 | 1.921130 |
| N | 3.663053 | 1.102015 | -0.291380 |
| C | -5.024964 | 0.634877 | 0.238977 |
| H | -5.832943 | 1.296333 | 0.502038 |
| C | -2.799175 | 1.079136 | 3.130326 |
| H | -3.132261 | 0.211897 | 2.551848 |
| C | 2.408592 | -1.095713 | 3.550046 |
| H | 1.934422 | -0.526770 | 2.745435 |
| H | 2.660150 | -0.389371 | 4.344635 |
| H | 1.673053 | -1.800746 | 3.947178 |
| C | 3.325979 | -2.822206 | 1.977807 |
| C | -0.748220 | 1.122876 | -0.405730 |
| H | -1.242698 | 2.024531 | -0.736339 |
| C | 3.040469 | 3.348212 | 0.409088 |
| C | 3.351375 | 2.456733 | -0.624410 |
| C | -3.258494 | -2.275606 | -0.909234 |
| C | 3.041405 | -3.346828 | -0.409706 |
| C | 0.622262 | -1.093786 | 0.405408 |
| H | 1.207342 | -1.943731 | 0.727788 |
| C | -2.357241 | -4.650377 | -1.915633 |
| H | -2.005584 | -5.593784 | -2.316968 |
| C | -3.259413 | 2.274540 | 0.909732 |
| C | -3.295957 | 3.395900 | 0.074284 |
| C | 4.383607 | 2.472459 | -4.251895 |
| H | 4.733537 | 1.698966 | -4.938284 |
| H | 5.249551 | 3.051245 | -3.925502 |
| H | 3.728078 | 3.134720 | -4.822330 |
| C | -2.338384 | 3.516777 | 2.709044 |
| H | -1.972643 | 3.583723 | 3.726842 |
| C | -3.797057 | -1.263900 | -4.272723 |
| H | -3.510790 | -2.101664 | -4.912221 |
| H | -3.832542 | -0.367126 | -4.894932 |
| H | -4.803917 | -1.460559 | -3.898581 |
| C | -3.813245 | -3.345666 | 1.346837 |
| H | -3.922943 | -2.295341 | 1.636598 |
| C | -2.794384 | 2.293322 | 2.227575 |
| C | -3.796407 | 1.263950 | 4.273857 |
| H | -3.510267 | 2.102097 | 4.912909 |
| H | -3.831254 | 0.367412 | 4.896438 |
| H | -4.803499 | 1.460010 | 3.900025 |
| C | -2.793829 | -2.293636 | -2.227220 |
| C | 2.664719 | -4.638888 | -0.051506 |
| H | 2.425828 | -5.358154 | -0.826705 |
| C | 4.160741 | -3.757955 | -2.612583 |
| H | 5.143146 | -3.643676 | -2.150467 |
| H | 4.232568 | -3.427496 | -3.651213 |
| H | 3.917276 | -4.823064 | -2.618261 |
| C | 3.099997 | -2.949604 | -1.868149 |
| H | 3.394405 | -1.898236 | -1.921197 |
| C | 1.735223 | -3.083742 | -2.542005 |
| H | 1.401811 | -4.124190 | -2.560673 |
| H | 1.786213 | -2.733155 | -3.575552 |
| H | 0.965180 | -2.503009 | -2.026568 |
| C | -5.196061 | 3.994699 | -1.435769 |
| H | -5.912434 | 3.529132 | -0.755187 |
| H | -5.590914 | 3.917086 | -2.450738 |
| H | -5.140789 | 5.054445 | -1.176789 |
| C | 3.661446 | 1.830505 | -3.072799 |
| H | 4.335378 | 1.082861 | -2.646444 |
| C | -1.402443 | 0.765288 | 3.662945 |
| H | -0.678259 | 0.638422 | 2.855099 |
| H | -1.420800 | -0.153535 | 4.253052 |
| H | -1.035862 | 1.563435 | 4.311611 |
| C | 4.967473 | -0.655035 | 0.169335 |
| H | 5.802672 | -1.316904 | 0.334655 |
| C | 2.606406 | 5.022738 | -1.278232 |
| H | 2.318829 | 6.035374 | -1.536971 |
| C | -0.747752 | -1.123078 | 0.406488 |
| H | -1.241864 | -2.024910 | 0.737168 |
| C | -2.849303 | -3.989185 | 2.341371 |

| | | | |
|---|---|---|---|
| H | -2.749178 | -5.062350 | 2.168911 |
| H | -3.222249 | -3.858504 | 3.359056 |
| H | -1.848466 | -3.551357 | 2.292919 |
| C | 4.385154 | -2.472683 | 4.251569 |
| H | 4.735010 | -1.699361 | 4.938186 |
| H | 5.251151 | -3.051327 | 3.925061 |
| H | 3.729628 | -3.135137 | 4.821790 |
| C | -2.799459 | -1.079108 | -3.129506 |
| H | -3.132674 | -0.212215 | -2.550581 |
| C | 2.407056 | 1.095617 | -3.549990 |
| H | 1.671466 | 1.800522 | -3.947256 |
| H | 1.932932 | 0.526878 | -2.745199 |
| H | 2.658593 | 0.389074 | -4.344407 |
| C | 3.324367 | 2.822590 | -1.978279 |
| C | -2.832265 | -4.592102 | -0.615452 |
| H | -2.845559 | -5.490787 | -0.010718 |
| C | 2.662981 | 4.639904 | 0.050415 |
| H | 2.424035 | 5.359428 | 0.825360 |
| C | -3.815296 | 3.343475 | -1.346627 |
| H | -3.924826 | 2.293001 | -1.635901 |
| C | 4.967254 | 0.656712 | -0.169393 |
| H | 5.802227 | 1.318830 | -0.334850 |
| C | 2.608872 | -5.022386 | 1.276973 |
| H | 2.321933 | -6.035297 | 1.535347 |
| C | -5.193831 | -3.997262 | 1.436065 |
| H | -5.910518 | -3.531547 | 0.755918 |
| H | -5.588410 | -3.920237 | 2.451184 |
| H | -5.138365 | -5.056868 | 1.176555 |
| C | -2.851752 | 3.986763 | -2.341697 |
| H | -2.751627 | 5.059985 | -2.169595 |
| H | -3.225054 | 3.855747 | -3.359210 |
| H | -1.850880 | 3.548992 | -2.293470 |
| C | -2.834344 | 4.591114 | 0.614887 |
| H | -2.848240 | 5.489551 | 0.009799 |
| C | 4.160082 | 3.761355 | 2.611457 |
| H | 3.915653 | 4.826245 | 2.616783 |
| H | 5.142446 | 3.647784 | 2.149080 |
| H | 4.232539 | 3.431392 | 3.650201 |
| C | -1.403067 | -0.764455 | -3.662536 |
| H | -1.036412 | -1.562150 | -4.311714 |
| H | -0.678616 | -0.637656 | -2.854922 |
| H | -1.422039 | 0.154628 | -4.252224 |
| C | -2.920377 | -0.000552 | 0.000466 |
| C | -1.467061 | -0.000243 | 0.000396 |
| N | 1.313467 | 0.000292 | 0.000277 |
| B | 2.812270 | 0.000522 | 0.000169 |

**$(HCDippN)_2B(NC_4H_4C)C(NMesCH)_2$ [2]**

122

| | | | |
|---|---|---|---|
| N | 3.324829 | -0.953395 | 0.327748 |
| N | 0.755272 | -0.597594 | 0.423381 |
| N | 2.035609 | -2.661744 | -0.461553 |
| N | -4.071863 | 1.434744 | 0.100083 |
| N | -2.732914 | 3.196126 | 0.104286 |
| C | 3.884580 | 0.263215 | 0.796366 |
| C | -1.647265 | 0.996558 | 0.468166 |
| C | 0.549462 | -4.588649 | -0.385507 |
| C | 0.768330 | 0.760566 | 0.785344 |
| H | 1.727193 | 1.166268 | 1.069176 |
| C | -1.657424 | -0.455221 | 0.372308 |
| H | -2.583681 | -1.008953 | 0.318822 |
| C | 4.119909 | 0.425452 | 2.167762 |
| C | 1.035657 | -3.465945 | -1.067745 |
| C | -2.730701 | 1.801263 | 0.247577 |
| C | -1.005380 | 3.729278 | -1.530119 |
| C | 3.385407 | -2.995747 | -0.588988 |
| H | 3.688098 | -3.941134 | -1.009927 |
| C | 4.166268 | 1.281236 | -0.124729 |
| C | 4.144874 | -1.989440 | -0.124146 |
| H | 5.218838 | -1.916328 | -0.061560 |
| C | -0.333479 | 1.519436 | 0.807530 |
| H | -0.213856 | 2.544656 | 1.128031 |
| C | -0.525016 | -1.170365 | 0.350259 |
| H | -0.553739 | -2.246449 | 0.273357 |
| C | -1.608773 | 3.967836 | -0.293471 |
| C | -4.636154 | 0.189712 | 0.476632 |
| C | 1.055727 | -4.955356 | 0.991060 |
| H | 1.802680 | -4.209588 | 1.270399 |
| C | -4.724924 | -0.146039 | 1.828872 |
| C | -0.441651 | -5.352503 | -0.994017 |
| H | -0.834819 | -6.223169 | -0.479826 |
| C | 4.876916 | 2.674997 | 1.715660 |
| H | 5.256372 | 3.624255 | 2.078003 |
| C | -5.086478 | -0.673672 | -0.523295 |
| C | 0.552536 | -3.112588 | -2.335378 |
| C | 3.799471 | -0.677999 | 3.150281 |
| H | 3.641189 | -1.589391 | 2.569433 |
| C | 0.121322 | 4.472026 | -1.863314 |
| H | 0.605314 | 4.285008 | -2.817224 |
| C | 4.659983 | 2.487912 | 0.360826 |
| H | 4.872622 | 3.294793 | -0.332564 |
| C | 3.903810 | 1.102662 | -1.603215 |
| H | 3.605421 | 0.063800 | -1.757349 |
| C | -5.633431 | -1.893168 | -0.143519 |
| H | -5.980831 | -2.575985 | -0.913231 |
| C | -1.128890 | 4.952114 | 0.573702 |

| | | | |
|---|---|---|---|
| C | -5.726292 | -2.266566 | 1.193272 |
| C | 4.613115 | 1.649550 | 2.608698 |
| H | 4.789835 | 1.804101 | 3.667393 |
| C | -1.540903 | 2.688978 | -2.460607 |
| H | -1.319175 | 1.687662 | -2.080814 |
| H | -1.096934 | 2.791779 | -3.451561 |
| H | -2.627400 | 2.760926 | -2.554259 |
| C | 0.645125 | 5.435876 | -1.011312 |
| C | -5.270754 | -1.379807 | 2.162761 |
| H | -5.341854 | -1.654282 | 3.211188 |
| C | -4.849537 | 2.574651 | -0.103161 |
| H | -5.919445 | 2.502301 | -0.198593 |
| C | -4.040517 | 3.638922 | -0.110364 |
| H | -4.252316 | 4.682028 | -0.270317 |
| C | -0.444151 | -3.900440 | -2.901743 |
| H | -0.839122 | -3.638138 | -3.877523 |
| C | -0.938815 | -5.011935 | -2.240561 |
| H | -1.715661 | -5.614677 | -2.698279 |
| C | -0.004195 | 5.674147 | 0.195946 |
| H | 0.386243 | 6.429379 | 0.871861 |
| C | 1.737271 | -6.321257 | 0.986815 |
| H | 1.033097 | -7.115496 | 0.723204 |
| H | 2.143465 | -6.550104 | 1.975713 |
| H | 2.557844 | -6.351272 | 0.266224 |
| C | -0.063856 | -4.901101 | 2.028403 |
| H | -0.529766 | -3.913451 | 2.058912 |
| H | 0.329090 | -5.120984 | 3.024733 |
| H | -0.845271 | -5.634125 | 1.809230 |
| C | 1.072532 | -1.901050 | -3.075250 |
| H | 1.861866 | -1.459150 | -2.463794 |
| C | -4.925910 | -0.308902 | -1.966806 |
| H | -5.480906 | 0.597283 | -2.222756 |
| H | -5.275387 | -1.116733 | -2.610699 |
| H | -3.875201 | -0.111712 | -2.197292 |
| B | 1.947705 | -1.343425 | 0.127413 |
| C | -4.217998 | 0.790243 | 2.879541 |
| H | -3.126465 | 0.855429 | 2.836597 |
| H | -4.508693 | 0.451385 | 3.874475 |
| H | -4.603140 | 1.801841 | 2.727931 |
| C | -6.271600 | -3.609251 | 1.577926 |
| H | -6.735173 | -3.582091 | 2.565909 |
| H | -5.472387 | -4.356535 | 1.610964 |
| H | -7.016326 | -3.957595 | 0.859715 |
| C | 4.947481 | -0.938923 | 4.119942 |
| H | 5.878944 | -1.143960 | 3.587199 |
| H | 4.718302 | -1.801398 | 4.751133 |
| H | 5.120322 | -0.086899 | 4.783043 |
| C | -1.786250 | 5.181822 | 1.899776 |
| H | -2.783096 | 5.618258 | 1.789091 |
| H | -1.190475 | 5.857552 | 2.514506 |
| H | -1.915608 | 4.239391 | 2.438067 |
| C | 2.502673 | -0.376410 | 3.900829 |
| H | 2.600375 | 0.538710 | 4.492356 |
| H | 2.257136 | -1.194833 | 4.583313 |
| H | 1.664661 | -0.242615 | 3.213873 |
| C | 5.161236 | 1.347101 | -2.432301 |
| H | 5.501030 | 2.383312 | -2.350343 |
| H | 4.963514 | 1.146777 | -3.488716 |
| H | 5.980029 | 0.699714 | -2.110055 |
| C | 1.901940 | 6.172452 | -1.364951 |
| H | 2.061236 | 6.192239 | -2.444633 |
| H | 2.772552 | 5.686148 | -0.912241 |
| H | 1.878789 | 7.202444 | -1.002984 |
| C | 1.693231 | -2.287197 | -4.414846 |
| H | 2.494347 | -3.017938 | -4.282641 |
| H | 2.113049 | -1.405442 | -4.906740 |
| H | 0.949970 | -2.721396 | -5.089439 |
| C | 2.752652 | 1.991912 | -2.068324 |
| H | 1.840471 | 1.798280 | -1.500247 |
| H | 2.541579 | 1.818323 | -3.127642 |
| H | 2.999845 | 3.050213 | -1.945155 |
| C | -0.018909 | -0.847857 | -3.249983 |
| H | -0.826372 | -1.218330 | -3.888641 |
| H | 0.390929 | 0.052113 | -3.716229 |
| H | -0.451725 | -0.571231 | -2.286877 |

**For EPR simulation:**

**$[(HCDippN)_2B(NC_4H_4C)C(NDippCH)_2]^{\bullet+}$ $[1]^{\bullet+}$**

140

| | | | |
|---|---|---|---|
| N | 9.15434373543757 | 11.93682870877447 | 3.67109215471535 |
| N | 8.92347354699295 | 10.07446914915655 | 2.33044512253287 |
| C | 9.07880191352909 | 13.25351308052618 | 4.25813237488727 |
| C | 8.86646198932185 | 10.81128712792764 | 4.46640966012809 |
| H | 8.78007748740632 | 10.89126738272625 | 5.53979318486658 |
| C | 7.40290558392967 | 8.86298028634328 | 0.82218087372941 |
| C | 8.72433255419977 | 9.11073842050864 | 1.2780871891580 |
| C | 8.73207304973959 | 9.71237529370569 | 3.67649846219658 |
| H | 8.51955588554315 | 8.69155241435011 | 3.95912669687676 |
| C | 10.14923549834735 | 13.73169589571136 | 5.05638065355375 |
| C | 7.89249152562195 | 14.01438219154912 | 4.07024938365705 |
| C | 9.21959348520633 | 11.92047405561534 | -0.19085449725546 |
| H | 8.69960557011855 | 10.97879015802980 | -0.29315162998264 |
| C | 7.80556615605591 | 15.27130841902630 | 4.69302493470503 |
| H | 6.90800599000472 | 15.86897415839214 | 4.57031859122286 |
| C | 9.84260447928495 | 8.43268801837307 | 0.73255610505465 |
| C | 11.41757922113451 | 12.91514684095379 | 5.31570517440569 |

| | | | |
|---|---|---|---|
| H | 11.38144557141854 | 12.02434164305732 | 4.68201772429108 |
| C | 6.18001788679400 | 9.52770709649900 | 1.46036761717398 |
| H | 6.52747474844970 | 10.15988375392286 | 2.28044547453696 |
| C | 10.00897873428859 | 14.99684567494695 | 5.65987273106015 |
| H | 10.81096842295715 | 15.38289545740881 | 6.28239337035665 |
| C | 10.16598409704500 | 13.53787023265404 | 1.24388995004930 |
| C | 6.70233465025682 | 13.47533991310967 | 3.27463159987361 |
| H | 7.06977705509477 | 12.70609657519132 | 2.58988254872640 |
| C | 11.25743075524751 | 8.64625482798410 | 1.27291643685197 |
| H | 11.17650465951451 | 9.24136876631787 | 2.18645894224669 |
| C | 8.85369763343664 | 15.76251992041395 | 5.48033734474034 |
| H | 8.76354190927690 | 16.73237249697046 | 5.96195298049013 |
| C | 8.32815156177101 | 7.31765502099464 | -0.82917930898402 |
| H | 8.17424875875734 | 6.62678418207154 | -1.65342804337180 |
| C | 7.23073006332031 | 7.95889018781152 | -0.24210191269732 |
| H | 6.23036739389635 | 7.74938495179974 | -0.61146886674332 |
| C | 9.61867123836884 | 7.54559054552430 | -0.33695453676337 |
| H | 10.45874588206802 | 7.02069191587207 | -0.78228560737884 |
| C | 12.70499692532415 | 13.69492173522545 | 4.95961541231037 |
| H | 12.70342158135640 | 14.03547436253100 | 3.91800654985366 |
| H | 13.58022609260033 | 13.05423149837666 | 5.11155019915098 |
| H | 12.83095134143355 | 14.57585801782252 | 5.59654232805035 |
| C | 11.48659583753890 | 12.43981556392996 | 6.78806564578010 |
| H | 11.63077228839283 | 13.28618180319443 | 7.46791688506213 |
| H | 12.32705092974317 | 11.75276501246213 | 6.93021889906109 |
| H | 10.57167606720731 | 11.92147097525261 | 7.09109445210204 |
| C | 5.68095085310438 | 12.79274121224719 | 4.21561994295249 |
| H | 6.14990335681477 | 11.99499966563334 | 4.79812380525248 |
| H | 4.86108953083560 | 12.35562528154346 | 3.63450692320624 |
| H | 5.25135795840392 | 13.51819182422876 | 4.91516475534277 |
| C | 11.94401393462710 | 7.31398516360467 | 1.65078892564399 |
| H | 11.32962497134733 | 6.72865577034927 | 2.34146170876032 |
| H | 12.90648552628594 | 7.50889000198045 | 2.13540958052896 |
| H | 12.13838649779288 | 6.69412948383777 | 0.76952688650475 |
| C | 6.00443431272320 | 14.54439778461728 | 2.41059440732991 |
| H | 5.47027023306711 | 15.27982887276168 | 3.02132310308475 |
| H | 5.26368803794849 | 14.06372642539519 | 1.76296772085495 |
| H | 6.71671370688318 | 15.08339451205911 | 1.77777535157669 |
| C | 12.12572161639409 | 9.44565355302525 | 0.27383817569316 |
| H | 12.32313363076913 | 8.86016279746301 | -0.63102228493016 |
| H | 13.09005830891748 | 9.70540361407486 | 0.72478477940162 |
| H | 11.63266092347002 | 10.37343048972730 | -0.03359623411071 |
| C | 5.41847830490672 | 10.43270734843631 | 0.46652537245132 |
| H | 6.06920476078003 | 11.20049084452751 | 0.03437352359134 |
| H | 4.59115665883702 | 10.93694840108462 | 0.97807561978823 |
| H | 4.99353247978306 | 9.85064794009769 | -0.35829210190375 |
| C | 5.22358070655391 | 8.47839767792393 | 2.07233054927113 |
| H | 4.77268833622305 | 7.84668844331251 | 1.30007369444381 |
| H | 4.41076942125301 | 8.97717882221272 | 2.61106661586029 |
| H | 5.75032891447884 | 7.82490025913913 | 2.77431185622781 |
| N | 10.73800530086656 | 14.30269758953034 | -3.63071940244359 |
| N | 10.89461729647987 | 16.07803450778977 | -2.32385647179866 |
| C | 10.23084321077003 | 13.92824371313533 | -1.18146353517164 |
| C | 10.84722577551288 | 12.94846955435751 | -4.14205518361776 |
| C | 11.09819832489861 | 15.38754719243308 | -4.42524653415122 |
| H | 11.26462637929696 | 15.27355374736593 | -5.48410782307400 |
| C | 11.80903247745449 | 17.41295897775279 | -0.46036934483962 |
| C | 10.70059987573474 | 17.04717440963437 | -1.26330147385111 |
| C | 11.19288880312560 | 16.47910242933947 | -3.62215759101925 |
| H | 11.42466539939574 | 17.50651859154708 | -3.85263055327940 |
| C | 9.76973832138839 | 12.40693501085080 | -4.88442665297422 |
| C | 12.08178707196562 | 12.27330785958620 | -3.97459451849111 |
| C | 10.50502982875665 | 14.32574060772914 | 0.17799293325419 |
| H | 11.00909003323565 | 15.25313558780867 | 0.40182719064934 |
| C | 12.19676977331402 | 10.98800712110674 | -4.53293208905139 |
| H | 13.12745290355232 | 10.43976844878240 | -4.42417340158398 |
| C | 9.41865910876347 | 17.62298403388017 | -1.11671305418429 |
| C | 8.48073430299019 | 13.18388079692255 | -5.16616999556709 |
| H | 8.47578987066428 | 14.07972089522783 | -4.53675258102177 |
| C | 13.19961804837155 | 16.81357974200386 | -0.68263069961730 |
| H | 13.07692479306953 | 15.87465763476435 | -1.23298176046880 |
| C | 10.59900565782668 | 14.72204230322442 | -2.31444882490973 |
| C | 9.94342295806143 | 11.11949690960417 | -5.42545326412861 |
| H | 9.14160857227791 | 10.67030060615064 | -6.00325240338793 |
| C | 9.55346222484297 | 12.66026466871410 | -1.29496977136866 |
| H | 9.26668251154950 | 12.26222310818635 | -2.25858537428147 |
| C | 13.29411508634371 | 12.92142619618141 | -3.30231967711047 |
| H | 12.96069058997360 | 13.82223152225446 | -2.77906784158249 |
| C | 8.25263199590821 | 17.28643731262899 | -2.04641802946609 |
| H | 8.58962640612875 | 16.53561269078462 | -2.76626297850845 |
| C | 11.13774647759004 | 10.41292877893890 | -5.24434131096799 |
| H | 11.25040440275581 | 9.42258235733175 | -5.67668919039718 |
| C | 10.32286621958746 | 18.94451392535887 | 0.72745587713544 |
| H | 10.17334684219777 | 19.68250683182401 | 1.51017644467083 |
| C | 11.59026895160835 | 18.37774933281951 | 0.53728130174982 |
| H | 12.41196953516495 | 18.68791222368730 | 1.17453438592614 |
| C | 9.25252120332157 | 18.57358373086626 | -0.09165655388859 |
| H | 8.28049339654225 | 19.03345543071941 | 0.05916314947515 |
| C | 7.20411965167821 | 12.37682974183837 | -4.83663812324917 |
| H | 7.18857922184814 | 12.03534152425124 | -3.79645737762197 |
| H | 6.32150507976200 | 13.00211925325707 | -5.00708459784333 |
| H | 7.10536268521596 | 11.49626295989429 | -5.47839627649539 |
| C | 8.43697843839399 | 13.65204051581321 | -6.64244150510939 |
| H | 8.34430754571800 | 12.79740404236031 | -7.32036241986418 |
| H | 7.57563923289120 | 14.30619357171438 | -6.81092426146206 |
| H | 9.33973700957218 | 14.20285198538036 | -6.92491577026396 |
| C | 14.32160889126537 | 13.36452765569241 | -4.37489492622670 |

| | | | |
|---|---|---|---|
| H | 13.86515632176361 | 14.03056140606979 | -5.11369043521481 |
| H | 15.15872073284932 | 13.89538259708388 | -3.90950019306874 |
| H | 14.72838463803315 | 12.50033040160437 | -4.91023626618727 |
| C | 7.81794682083126 | 18.52754619364008 | -2.86121320168728 |
| H | 8.66226215946767 | 18.97422349812781 | -3.39577044419105 |
| H | 7.05893448960987 | 18.24535829938787 | -3.59811089687778 |
| H | 7.38525819039497 | 19.29883165796675 | -2.21633160950785 |
| C | 13.95411778000195 | 12.00993745671153 | -2.24777336944383 |
| H | 14.40664266341259 | 11.12228172436328 | -2.70132914152174 |
| H | 14.75155196979784 | 12.55613460259396 | -1.73274444299593 |
| H | 13.22883807859947 | 11.67679397577790 | -1.50021335509516 |
| C | 7.05475859577464 | 16.67866631238888 | -1.28411484919366 |
| H | 6.64861704510486 | 17.37961241651994 | -0.54790058851859 |
| H | 6.25060784790735 | 16.43094559496924 | -1.98459737482602 |
| H | 7.34309842545286 | 15.76440996820160 | -0.75705631613135 |
| C | 13.94401511552532 | 16.48200244441899 | 0.62991145267685 |
| H | 13.33694993226701 | 15.86661240317785 | 1.30103097282933 |
| H | 14.86250688303754 | 15.93030745561097 | 0.40620642754653 |
| H | 14.23573107479390 | 17.38648085840904 | 1.17284349734911 |
| C | 14.05892232287781 | 17.75308931965630 | -1.56234843358039 |
| H | 14.29753352232387 | 18.67575749034100 | -1.02297376887038 |
| H | 15.00158439812966 | 17.26706572140513 | -1.83613974213436 |
| H | 13.54095157110205 | 18.03338157541627 | -2.48498476446519 |
| H | 10.40983867434662 | 13.83920767449494 | 2.25310315705225 |
| N | 9.51824546807696 | 12.31550315283241 | 1.11029820269366 |
| B | 9.20492485124716 | 11.48565824550135 | 2.30118141774144 |

## 9. Supplementary References


(1) Herrmanndörfer, D., Kaaz, M., Puntigam, O., Bender, J., Nieger, M. & Gudat, D. The Reaction between Diazadienes and Element Tribromides $EBr_3$ (E = P, B) Revisited: Metal-Free Synthesis of Halogenated N-Heterocyclic Phosphanes and Boranes. *Eur. J. Inorg. Chem.* 4819−4828 (2015).

(2) Stoll, S. & Schweiger, A. EasySpin, A Comprehensive Software Package for Spectral Simulation and Analysis in EPR. *J. Magn. Reson.* **178**, 42−55 (2006).

(3) Neese, F. The ORCA Program System. *WIREs Comput. Mol. Sci.* **2**, 73−78 (2012).

(4) Neese, F. Software Update: the ORCA Program System, Version 4.0. *WIREs Comput. Mol. Sci. 8*, e1327 (2017).

(5) Barone V. *Recent Advances in Density Functional Methods*; World Scientific: Singapore, 1995.

(6) Weigend, F. Accurate Coulomb-Fitting Basis Sets for H to Rn. *Phys. Chem. Chem. Phys.* **8**, 1057−1065 (2006).

(7) Cosier, J. & Glazer, A. M. A Nitrogen-Gas-Stream Cryostat for General X-Ray Diffraction Studies. *J. Appl. Crystallogr.* **19**, 105−107 (1986).

(8) CrysAlisPro, Agilent Technologies, Version 1.171.35.8.

(9) Sheldrick, G. M. SHELXT - Integrated Space-Group and Crystal Structure Determination. *Acta Cryst. Sect. A* **71**, 3−8 (2015).

(10) Sheldrick, G. M. *Acta Cryst. Sect. A* **2008**, *64*, 112−122.

(11) Barbour, L. J. X-seed – A software tool for supramolecular crystallography. *J. Supramol. Chem.* **1**, 189-191 (2001).

(12) Dolomanov, O. V., Bourhis, L. J., Gildea, R. J., Howard, J. A. K. & Puschmann, H. OLEX2: a complete structure solution, refinement and analysis program. *J. Appl. Crystallogr.* **42**, 339-341 (**2009**).

(13) Frisch, M. J., Trucks, G. W., Schlegel, H. B., Scuseria, G. E., Robb, M. A., Cheeseman, J. R., Scalmani, G., Barone, V., Mennucci, B., Petersson, G. A., Nakatsuji, H., Li, X., Caricato, M., Li, X., Hratchian, H. P., Izmaylov, A. F., Bloino, J., Zheng, G., Sonnenberg, J. L., Hada, M., Ehara, M., Toyota, K., Fukuda, R., Hasegawa, J., Ishida, M., Nakajima, T., Honda, Y., Kitao, O., Nakai, H., Vreven, T., Montgomery, J. A., Jr., Peralta, J. E., Ogliaro, F., Bearpark, M. J., Heyd, J. J., Brothers, E. N., Kudin, K. N., Staroverov, V. N., Kobayashi, R., Normand, J., Raghavachari, K., Rendell, A. P., Burant, J. C., Iyengar, S. S., Tomasi, J., Cossi, M., Rega, N., Millam, J. M., Klene, M., Adamo, C., Jaramillo, J., Gomperts, R., Stratmannm R. E., Yazyev, O, Austin, A.J., Cammi, R., Pomelli, C., Ochterski, J. W., Martin, R. L., Morokuma, K., Zakrzewski, V. G., Voth, G. A., Salvador, P, Dannenberg, J. J., Dapprich, S., Daniels, A. D., Farkas, O., Foresman, J. B., Ortiz, J. V., Cioslowski, J. & Fox, D. J. *Gaussian 09*, *Revision D.01*, Gaussian, Inc., Wallingford, CT 2009.

(14) Perdew, J. P., Burke, K. & Ernzerhof, M. Generalized Gradient Approximation Made Simple. *Phys. Rev. Lett.* **77**, 3865−3868 (1996).

(15) Perdew, J. P. & Burke, K., Ernzerhof, Generalized Gradient Approximation Made Simple [Phys. Rev. Lett. 77, 3865 (1996)]. *Phys. Rev. Lett.* **78**, 1396 (1997).

(16) Adamo, C. & Barone, V. Toward Reliable Density Functional Methods Without Adjustable Parameters: The PBE0 Model. *J. Chem. Phys.* **110**, 6158−6170 (1999).

(17) Schaefer, A., Horn, H. & Ahlrichs, R. Fully Optimized Contracted Gaussian Basis Sets for Atoms Li to Kr. *J. Chem. Phys.* **97**, 2571−2577 (1992).

(18) Schaefer, A., Huber, C. & Ahlrichs, R. Fully Optimized Contracted Gaussian Basis Sets of Triple Zeta Valence Quality for Atoms Li to Kr. *J. Chem. Phys.* **100**, 5829−5835 (1994).

(19) Grimme, S., Ehrlich, S. & Goerigk, L. Effect of the Damping Function in Dispersion Corrected Density Functional Theory. *J. Comput. Chem.* **32**, 1456−1465 (2011).

(20) NBO 7.0. Glendening, E. D., Badenhoop, J. K., Reed, A. E., Carpenter, J. E., Bohmann, J. A., Morales, C. M., Karafiloglou, P., Landis, C. R. & Weinhold, F. Theoretical Chemistry Institute, University of Wisconsin, Madison, WI, 2018.
