# Supplementary material for: A crystalline radical cation derived from Thiele’s hydrocarbon with redox range beyond 1 V
Source: Nat Commun. 2021 Dec 3;12:7052. doi: 10.1038/s41467-021-27104-y (PMC8642399; doi:10.1038/s41467-021-27104-y)
Supplement: Supplementary file 2 — Peer Review File [file 41467_2021_27104_MOESM2_ESM.pdf]

nature portfolio

Peer Review File

A Crystalline Radical Cation Derived from Thiele's Hydrocarbon with Redox Range Beyond 1 V

Open Access This file is licensed under a Creative Commons Attribution 4.0 International License, which permits use, sharing, adaptation, distribution and reproduction in any medium or format, as long as you give appropriate credit to the original author(s) and the source, provide a link to the Creative Commons license, and indicate if changes were made. In the cases where the authors are anonymous, such as is the case for the reports of anonymous peer reviewers, author attribution should be to 'Anonymous Referee' followed by a clear attribution to the source work. The images or other third party material in this file are included in the article's Creative Commons license, unless indicated otherwise in a credit line to the material. If material is not included in the article's Creative Commons license and your intended use is not permitted by statutory regulation or exceeds the permitted use, you will need to obtain permission directly from the copyright holder. To view a copy of this license, visit http://creativecommons.org/licenses/by/4.0/.

REVIEWER COMMENTS

Reviewer #1 (Remarks to the Author):

This manuscript describes the preparation of an unsymmetrical organoboron derivatives of Tiele's hydrocarbon. N-heterocyclic carbene (NHC) and its boron analogue (NHB) have been utilized as synthons in synthesis. Cyclic voltammetry revealed two reversible single-electron oxidations. In fact, upon oxidation by AgSbF6 (1 eq and 2 eq), corresponding radical cation and dication were isolated and fully characterized. EPR spectrum of radical cation fits well with simulated one. Obtained hyperfine coupling constant for 11B, 14N, 1H together with calculated spin density strongly suggest the delocalization of the radical across the entire pi-system. A beautiful gradual red-shifting was shown in the UV-vis spectra of neutral, radical cation and dication, and TD-DFT was also provided/discussed.
In summary, this is a great contribution by Aldridge group and the manuscript including a significant breakthrough in the main group/organic chemistry is very well discussed. Thus, I recommend for the acceptance in Nature Comm.
Following minor points can be considered for the revised version:

1) NMR: 11B NMR for neutral compounds (1 and 2) and dication 2++ should be discussed/mentioned (whilst they showed more or less similar values). This can be related to the resonance structure(s).

2) DFT: considering the resonance structures for radical cation and dication, NRT calculations can be used. Also, the aromaticity of these species (they have three rings in the molecules) would be worth to discuss. For example, NICS values can be used (if possible).

Reviewer #2 (Remarks to the Author):

This paper reports synthesis of an unsymmetrical BN analogue of Thiele's hydrocarbon and its one- and two-electron oxidation reactions. The present molecules are very interesting and attractive considering their stepwise oxidation. This characteristic property is very strong point of this paper. In contrast, the unavoidable disorder due to the same substituents on NHC and NHB moieties in crystallographic study for radical cation and dication would be the weakest point. If the authors would provide crystallographic analysis of all the products without disorder due to the same substituents on NHC and NHB moieties, the reviewer would like to suggest to publish this paper in Nat. Commun. with minor revision. Please consider the following points.

1) As pointed out above, the reviewer strongly suggests to get crystal structures of radical cation and dication prepared from 2, instead of those from 1. The detailed analysis of bond lengths and angles should provide further understanding the bonding situation. Structural parameters, which would be obtained from non-disordered crystal structure, should be related to the appropriateness of two resonance structures in Schemes 3 and 4. On the assumption that the authors would provide these data, the reviewer guesses the third resonance structure (on the right) in each Scheme would be acceptable only in the case the C-N bond lengths in NHB moiety would be shortened in comparison with those of the neutral precursor. Also, these resonance structures have no contribution of B=N double bond, although the B-N bond lengths seem to be relatively short.
2) In Figure 2, the boron nucleus has a negative distribution of the spin density. Please explain this negative value.

Reviewer #3 (Remarks to the Author):

The structures are chemically reliable, but data could probably be improved significantly. The data processing software version is outdated by three years. I strongly recommend to repeat the data reduction employing the latest version of CrysAlisPro. 3D profile fitting and smart background

treatment in this version should help to improve the data of 2, but also of the other two structures. An analytical absorption correction from crystal faces would also be very advisable, especially for the [SbF6]- containing compounds.

Reviewer #4 (Remarks to the Author):

In this contribution, Aldridge et al. report on very exciting unsymmetrical BN-embedded Thiele's hydrocarbon derivatives where the CPh2 substituents are an NHC and an NHB. This substitution pattern has never been as convincingly demonstrated as in this manuscript. While bis-NHC-and bis-boryl-substituted Thiele's hydrocarbon systems have been recently disclosed, their stable redox states are limited to one or two. Such a hybrid NHC/NHB strategy provides access to the systems with three stable redox states. Of note, this approach gives rise to a significantly enhanced redox range (> 1.0 V), thereby preventing disproportionation of the intervening radical cation. The literature is properly cited although a relevant paper involving a bis-BN analogue has been left out (Yamashita et al. Chem. Sci. 2018).
Given that many organic mixed-valence systems with three stable redox states have fueled a variety of fields, the manuscript will be of high interest to the organic, inorganic and material communities. In my opinion, this study is suited for an article in Nat. Comm. provided that the following points are addressed:
1. The experimental work is well done, but the theoretical support is modest. The radical cation will be resistant towards disproportionation if the oxidative formation of the radical cation from the neutral state is more favorable than further oxidation to the dication. The separation of the two redox waves in experimental CVs is related to chemical potential change (delta µ), which is easily calculated by frontier molecular orbital energies. How does the BN/CC isosterism effect the FMO energies and thus widen the redox range? Can the authors provide rationale?
2. As shown in Scheme 2, species 1 and 2 have biradical character (open shell singlet or triplet). Can the authors calculate it? How does it compare with their bis-NHC analogs?
3. Are there any experimental evidences for the formation of a FLP consisting of {(HCDippN)2B}OTf and IDipp? As NHCs and CAACs are known to react with pyridinium salts, {(HCDippN)2B}OTf may form a salt with pyridine followed by para-attack by NHC.
4. 13C NMR data are not given for (HCDippN)2BOTf.
5. No EA are given for (HCDippN)2BOTf and 1.
6. Please indicate the contribution of each transition from TD-DFT calculations.

Reviewer #1 (Remarks to the Author):

This manuscript describes the preparation of an unsymmetrical organoboron derivatives of Tiele's hydrocarbon. N-heterocyclic carbene (NHC) and its boron analogue (NHB) have been utilized as synthons in synthesis. Cyclic voltammetry revealed two reversible single-electron oxidations. In fact, upon oxidation by AgSbF6 (1 eq and 2 eq), corresponding radical cation and dication were isolated and fully characterized. EPR spectrum of radical cation fits well with simulated one. Obtained hyperfine coupling constant for 11B, 14N, 1H together with calculated spin density strongly suggest the delocalization of the radical across the entire pi-system. A beautiful gradual red-shifting was shown in the UV-vis spectra of neutral, radical cation and dication, and TD-DFT was also provided/discussed.
In summary, this is a great contribution by Aldridge group and the manuscript including a significant breakthrough in the main group/organic chemistry is very well discussed. Thus, I recommend for the acceptance in Nature Comm.
Following minor points can be considered for the revised version:

1) NMR: 11B NMR for neutral compounds (1 and 2) and dication 2++ should be discussed/mentioned (whilst they showed more or less similar values). This can be related to the resonance structure(s).
Response: we have added in the chemical shifts for compound **1**, **2**, and [**1**]$^{2+}$ into the manuscript proper. While in theory these data might be used to inform the discussion of electronic structure - in reality (as this reviewer points out) the chemical shifts for these three species are essentially identical**.**

2) DFT: considering the resonance structures for radical cation and dication, NRT calculations can be used. Also, the aromaticity of these species (they have three rings in the molecules) would be worth to discuss. For example, NICS values can be used (if possible).
Response: We have carried out both NRT and NICS (NICS(0) and NICS(1)) calculations as set out below (details of these are also included in the revised supporting information). To summarize: the NICS calculations are certainly informative as to the aromatic/non-aromatic/anti-aromatic character of the three systems, and have now been included in the revised manuscript. Using the NRT approach to determine the weight attached to *individual* resonance structures proves to be more difficult to apply in these highly delocalized systems (even when the model is simplified to remove the N-bound Dipp groups), with >100 resonance structures being obtained for all three systems (neutral, radical cation, dication), and the most heavily weighted contributing structure being <6% in all cases. However, where the NRT approach does prove to be very useful is in providing natural bond orders for each of the bonds (B1-N6, C9-C12 etc. - as below) in the neutral, radical cationic and dicationic species. In reality this provides a much more useful approach for discussing the bond orders within the three molecules, summed over the various resonance forms. Indeed, this approach provides an informative basis for a more extended discussion of resonance structures for **1**, **1**$^{\bullet+}$ and **1**$^{2+}$, of the type suggested by reviewer 2.

NICS(0) and NICS(1) Calculations (For full systems with N-bound Dipp substituents)

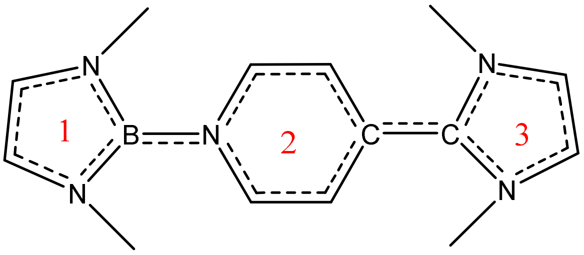

Table S3. NICS(0) and NICS(1) data for **1**, **1•+** and **12+**.

	**1**	**1•+**	**12+**
Ring 1 NICS(0)	-8.1382	-9.0253	-9.001
Ring 1 NICS(1)	-4.1778	-5.0367	-7.111
Ring 2 NICS(0)	6.7618	1.522	-7.5506
Ring 2 NICS(1)	4.137	-0.8654	-8.9863
Ring 3 NICS(0)	-7.0003	-10.135	-12.3617
Ring 3 NICS(1)	-2.4116	-5.3367	-10.3018

<u>**NRT calculations**</u>
(Dipp-substituents replaced with H. The simplified model compounds were first optimized by freezing all heavy atoms and only allowing the hydrogen atoms to move)

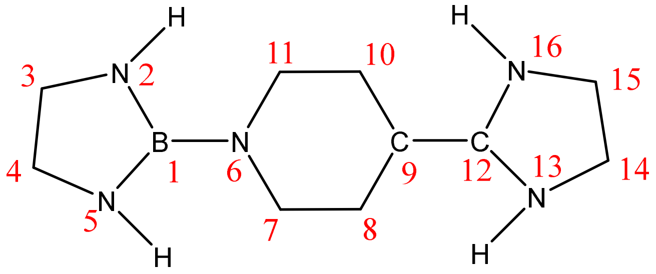

Table S4. Natural Bond Orders for **1**, **1•+** and **12+**.

Bond	Order (neutral)	Order (radical)	Order (dication)
B1-N6	1.0879	1.0029	1.0075
B1-N2	1.3596	1.3831	1.3439
N2-C3	1.2521	1.2526	1.3418
C3-C4	1.6148	1.6086	1.5482
C4-N5	1.2483	1.2565	1.3418
N5-B1	1.3566	1.4135	1.3439
N6-C7	1.1628	1.3040	1.3928
C7-C8	1.7517	1.6367	1.4788
C8-C9	1.1413	1.2922	1.4544
C9-C10	1.1444	1.2869	1.4544
C10-C11	1.7537	1.6527	1.4788
C11-N6	1.1610	1.2948	1.3928
C9-C12	1.4440	1.3029	1.0356
C12-N13	1.1320	1.2261	1.3777
N13-C14	1.2211	1.2927	1.3466
C14-C15	1.6285	1.5925	1.5678
C15-N16	1.2442	1.2901	1.3466
N16-C12	1.1267	1.2230	1.3777

<u>Neutral **1**:</u>
3-Center, 4-Electron A:-B-:C Hyperbonds (A-B :C <=> A: B-C)
Hyperbond A:-B-:C %A-B/%B-C
1. N6:- B1-: N5 45.7/54.3
2. N6:- B1-: N2 45.6/54.4

Resonance structures: Over 125 resonance structures found in total, which is in agreement with highly delocalized system, parent structure has a weight of only 5.92%. The 20 highest contributions total to 44.46%, of

which ca. 54% have C9-C12 double bond and either C7-C8 or C10-C11 double bond and ca. 39% feature structures that exhibit 3 C-C/C-N double bonds in ring 2.

Table S5. Natural Atomic Valencies and Charges for **1**.

Atom	Valency	Covalent valency	Ionic valency	Formal charge
B1	3.8041	1.6819	2.1223	-1.1331
N2	3.6005	2.0539	1.5466	0.6867
C3	3.8063	3.2182	0.5882	-0.1937
C4	3.8066	3.2200	0.5866	-0.1850
N5	3.5939	2.0497	1.5442	0.6760
N6	3.4117	2.0892	1.3224	0.5867
C7	3.8642	3.2671	0.5971	-0.1358
C8	3.8478	3.5466	0.3012	-0.1276
C9	3.8152	3.5401	0.2751	-0.0620
C10	3.8545	3.5490	0.3055	-0.1386
C11	3.8627	3.2671	0.5956	-0.1304
C12	3.7215	3.0116	0.7099	-0.2677
N13	3.3881	2.2675	1.1206	0.4602
C14	3.7960	3.2218	0.5742	-0.1837
C15	3.8289	3.2307	0.5982	-0.1687
N16	3.3921	2.2755	1.1167	0.5190

Radical monocation $\mathbf{1}^{\cdot+}$: (NBO treats the α and β spin orbitals separately)
3-Center, 4-Electron A:-B-:C Hyperbonds (A-B :C <=> A: B-C)
α-spin
Hyperbond A:-B-:C   %A-B/%B-C
1. N2:- B1-: N5 50.2/49.8

β-spin
Hyperbond A:-B-:C   %A-B/%B-C
1. N13:-C14-: C15 27.0/73.0
2. C12:- N16-: C15 54.6/45.4

Resonance structures: (α-spin) Over 180 resonance structures found in total, which is in agreement with highly delocalized system, parent structure has a weight of only 1.73%. The 20 highest contributions total 24.84% of the calculated resonance structures, of which: ca. 4% exhibit a C9-C12 single bond and C7-C8 and C10-C11 double bonds (resembling structure $1^{\cdot+}$a). Ca. 66% of structures have a C9-C12 double bond and two C-C double bonds in ring 2 (resembling structure $1^{\cdot+}$b). Ca. 30% of the structures feature 3 double bonds in the ring 2 with cationic charge localized on N6 (resembling structure $1^{\cdot+}$c) (β-spin) Over 162 resonance structures found in total, which is in agreement with highly delocalized system, parent structure has a weight of only 2.52%. The 20 highest contributions total 43.41% of the calculated resonance structures, of which all feature 3 double bonds in the ring 2 and C9-C12 single bond (resembling structure $1^{\cdot+}$c).

Table S6. Natural Atomic Valencies and Charges for $\mathbf{1}^{\cdot+}$

Atom	Valency	Covalent valency	Ionic valency	Formal charge
B1	3.8147	1.7268	2.0879	-1.1439
N2	3.6462	2.0818	1.5644	0.7123
C3	3.8156	3.1924	0.6232	-0.1808
C4	3.8209	3.1961	0.6248	-0.1758
N5	3.6677	2.0688	1.5990	0.7443
N6	3.6343	2.3117	1.3225	0.7619
C7	3.9264	3.2764	0.6500	-0.0558
C8	3.8987	3.5310	0.3677	-0.0434
C9	3.9034	3.5710	0.3324	-0.0374
C10	3.8918	3.5197	0.3721	-0.0632
C11	3.9118	3.2494	0.6624	-0.0612
C12	3.7921	3.0052	0.7868	-0.1956
N13	3.5645	2.3921	1.1724	0.6075
C14	3.8372	3.1958	0.6413	-0.1552
C15	3.8304	3.1923	0.6381	-0.1572
N16	3.5565	2.3945	1.1621	0.5994

Dication $\mathbf{1}^{2+}$:
3-Center, 4-Electron A:-B-:C Hyperbonds (A-B :C <=> A: B-C)
Hyperbond A:-B-:C   %A-B/%B-C
1. N13:- C14-: C15 24.4/75.6

2. C 12:- N16-: C15 54.4/45.6

Resonance structures: Over 128 resonance structures in total. Parent structure has a weight of only 2.48%. 24 biggest contributions total 45.02% of the overall structures, of which: ca 71% exhibit 3 C-C/C-N double bonds in the ring 2 (resembling structure 1^{2+}a). Ca. 23% resemble structure 1^{2+}b and ca. 6% of resonance structures have C9-C12 double bond and either C7-C8 or C10-C11 double bond (resembling structure 1^{2+}c).

Table S7. Natural Atomic Valencies and Charges for $\mathbf{1^{2+}}$

Atom	Valency	Covalent valency	Ionic valency	Formal charge
B1	3.7751	1.7370	2.0381	-1.0661
N2	3.6791	2.1051	1.5740	0.7849
C3	3.8274	3.1369	0.6905	-0.1708
C4	3.8274	3.1369	0.6905	-0.1708
N5	3.6791	2.1051	1.5740	0.7849
N6	3.7931	2.3914	1.4017	0.8602
C7	3.9138	3.1961	0.7177	-0.0570
C8	3.9559	3.5856	0.3703	-0.0264
C9	3.9443	3.7775	0.1668	-0.0113
C10	3.9559	3.5856	0.3703	-0.0264
C11	3.9138	3.1961	0.7177	-0.0570
C12	3.7909	2.9989	0.7920	-0.1866
N13	3.7052	2.4466	1.2586	0.7701
C14	3.8757	3.1723	0.7034	-0.1146
C15	3.8757	3.1723	0.7034	-0.1146
N16	3.7052	2.4466	1.2586	0.7701

Frontier orbital energies
Neutral (Dipp substituents on both ends):
HOMO: -0.12611 au = -3.432 eV = -331.1 kJ/mol
LUMO: -0.01229 au = -0.3344 eV = -32.3 kJ/mol
HOMO-LUMO gap: 3.097 eV = 298.8 kJ/mol
Monocation radical
SOMO (alpha): -0.25129 au = -6.837 eV = -659.76 kJ/mol
LUMO (alpha): -0.12027 au = -3.273 eV = -315.77 kJ/mol
LUMO (beta): -0.18070 au = -4.9171 eV = -474.43 kJ/mol
SOMO-LUMO (alpha) gap: 0.13102 au = 3.564 eV = 343.99 kJ/mol
Dication
HOMO: -0.40190 au = -10.936 eV = -1055.19 kJ/mol
LUMO: -0.30782 au = -8.376 eV = -808.18 kJ/mol
HOMO-LUMO gap: 0.09408 au = 2.56 eV = 247.0 kJ/mol

Reviewer #2 (Remarks to the Author):

This paper reports synthesis of an unsymmetrical BN analogue of Thiele's hydrocarbon and its one- and two-electron oxidation reactions. The present molecules are very interesting and attractive considering their stepwise oxidation. This characteristic property is very strong point of this paper. In contrast, the unavoidable disorder due to the same substituents on NHC and NHB moieties in crystallographic study for radical cation and dication would be the weakest point. If the authors would provide crystallographic analysis of all the products without disorder due to the same substituents on NHC and NHB moieties, the reviewer would like to suggest to publish this paper in Nat. Commun. with minor revision. Please consider the following points.

1) As pointed out above, the reviewer strongly suggests to get crystal structures of radical cation and dication prepared from 2, instead of those from 1. The detailed analysis of bond lengths and angles should provide further understanding the bonding situation. Structural parameters, which would be obtained from non-disordered crystal structure, should be related to the appropriateness of two resonance structures in Schemes 3 and 4. On the assumption that the authors would provide these data, the reviewer guesses the third resonance structure (on the right) in each Scheme would be acceptable only in the case the C-N bond lengths in NHB moiety would be shortened in comparison with those of the neutral precursor. Also, these resonance structures have no contribution of B=N double bond, although the B-N bond lengths seem to be relatively short.
Response: The idea of obtaining structural data on compounds $\mathbf{2}^{\bullet+}$ and $\mathbf{2}^{2+}$ in order to get round the issues related to crystallographic disorder in $\mathbf{1}^{\bullet+}$ and $\mathbf{1}^{2+}$ is indeed a good one - and is similar to the approach we took in synthesizing/characterizing the neutral compound **2** as a surrogate for **1**. Indeed, with this in mind we spent a good deal of time both prior to submission of the original manuscript, and in the three-month revision period trying to obtain samples of these two species (i.e. $\mathbf{2}^{\bullet+}$ and $\mathbf{2}^{2+}$) suitable for single crystal X-ray diffraction. Unfortunately - and in much the same way that we saw for the neutral compound **1** - crystals of $\mathbf{2}^{\bullet+}$ and $\mathbf{2}^{2+}$ gave (at best) very weak diffraction, and we have not been able to obtain molecular structures of these systems in the solid state.
In response to the reviewer's point regarding structural data on the mono- and dications - of course the solid-state structures of the two disordered system do yield some metrical data that are relevant to the discussion of resonance structures for $\mathbf{1}^{\cdot+}$ and $\mathbf{1}^{2+}$, in that it is still possible to gauge the extent of aromaticity in the pyridyl ring through consideration of bond-length alternation. In addition, the mean exocyclic CC/BN distances for the three systems **1**, $\mathbf{1}^{\bullet+}$ and $\mathbf{1}^{2+}$ also allow for discussion of the trend in mean exocyclic double/single bond character (as do the inter-plane torsion angles). That is not to say that non-disordered structures would not offer a more complete basis for structural discussions - the data pertaining to the individual exocyclic BN and CC bonds would of course be beneficial. In the absence of these data, however, the NRT calculations suggested by reviewer 1 (and the natural bond orders derived therefrom) offer a powerful alternative method to use for the discussion of the weighting attached to different possible resonance structures. This approach can be augmented by consideration of the DFT-calculated exocyclic BN and CC bond lengths for for $\mathbf{1}^{\bullet+}$ and $\mathbf{1}^{2+}$ (which we have added in to Table 1). Indeed, it could be argued that for systems such as these (containing only lighter elements from the first period) NRT offers a more definitive approach for the discussion of contributing resonance forms than does an analysis based solely on crystallographic data. As such, we believe that the (revised) discussions of the possible contributing resonance forms for compounds $\mathbf{1}^{\bullet+}$ and $\mathbf{1}^{2+}$ (and the associated revised resonance schemes) are now on a rigorous footing. In particular, resonance descriptions of $\mathbf{1}^{\bullet+}$ and $\mathbf{1}^{2+}$ as containing B-N single bonds fit well with the calculated natural bond orders for this linkage (in both systems) which are close to one. In addition, the very minor contribution of the left-hand resonance structure $\mathbf{1}^{2+}$c (Scheme 4) - is (as the reviewer suggests) borne out by the NRT analysis.

2) In Figure 2, the boron nucleus has a negative distribution of the spin density. Please explain this negative value.
Response: The negative spin densities, rho_alpha - rho_beta < 0, at the boron atom are the result of spin polarisation of underlying orbitals by the SOMO. This effect from the SOMO to s-character orbitals results in large Fermi-contact hyperfine interactions. Some literature calls this a Coulomb and others an exchange interaction, regardless it amounts to a spatial difference in alpha and beta orbitals and can amount a change in sign of the overall spin density.
Tabulated below are the Mulliken spin densities of (one) imidazole nitrogen and the C2 carbon, and the corresponding nitrogen and boron of the opposing diazaboroline.

```
 68 N s       :     0.003628  s :     0.003628
      pz      :     0.003671  p :     0.092723
      px      :     0.074178
      py      :     0.014874
      dz2     :    -0.000058  d :    -0.000106
      dxz     :     0.000042
      dyz     :    -0.000021
      dx2y2   :    -0.000054
      dxy     :    -0.000016
 69 C s       :     0.028328  s :     0.028328
```

```
      pz      :     0.012251  p :     0.205783
      px      :     0.178688
      py      :     0.014844
      dz2     :    -0.000083  d :     0.001258
      dxz     :     0.000810
      dyz     :     0.000220
      dx2y2   :     0.000004
      dxy     :     0.000306

  1 N s       :     0.001527  s :     0.001527
      pz      :     0.003241  p :     0.030199
      px      :     0.024977
      py      :     0.001980
      dz2     :     0.000003  d :    -0.000058
      dxz     :    -0.000002
      dyz     :    -0.000008
      dx2y2   :    -0.000026
      dxy     :    -0.000025
139 B s       :    -0.004699  s :    -0.004699
      pz      :    -0.003315  p :    -0.012645
      px      :    -0.007654
      py      :    -0.001676
      dz2     :     0.000181  d :     0.003220
      dxz     :     0.001720
      dyz     :     0.000141
      dx2y2   :     0.000355
      dxy     :     0.000823
```

Reviewer #3 (Remarks to the Author):

The structures are chemically reliable, but data could probably be improved significantly. The data processing software version is outdated by three years. I strongly recommend to repeat the data reduction employing the latest version of CrysAlisPro. 3D profile fitting and smart background treatment in this version should help to improve the data of 2, but also of the other two structures. An analytical absorption correction from crystal faces would also be very advisable, especially for the $[SbF_6]^-$ containing compounds.
Response: The data reduction for all three structures has been repeated using the latest version of CrysAlisPro (v. 41.93a). 3D profile fitting and smart background treatment has been implemented in all cases. In addition, an analytical absorption correction (Clark and Reid) from the crystal faces has been performed for all three datasets, as suggested by the reviewer. This has indeed improved the quality of the data as proposed.

Neutral compound - Rint improved from 12.42% to 12.06%; R1 improved from 6.06% to 5.98%
Monocation - Rint improved from 8.57% to 7.80%; R1 improved from 5.00% to 4.67%.
Dication - Rint improved from 3.63% to 3.43%; R1 improved from 3.62% to 3.43%.

The new cif files have been updated on CCDC (Deposition Numbers 2063252-2063254).

Reviewer #4 (Remarks to the Author):

In this contribution, Aldridge et al. report on very exciting unsymmetrical BN-embedded Thiele's hydrocarbon derivatives where the CPh2 substituents are an NHC and an NHB. This substitution pattern has never been as convincingly demonstrated as in this manuscript. While bis-NHC-and bis-boryl-substituted Thiele's hydrocarbon systems have been recently disclosed, their stable redox states are limited to one or two. Such a hybrid NHC/NHB strategy provides access to the systems with three stable redox states. Of note, this approach gives rise to a significantly enhanced redox range (> 1.0 V), thereby preventing disproportionation of the intervening radical cation. The literature is properly cited although a relevant paper involving a bis-BN analogue has been left out (Yamashita et al. Chem. Sci. 2018).
Response: This additional reference has now been cited (as new ref 45) (Katsuma, Y., Asakawa, H., Yamashita, M. Reactivity of highly Lewis acidic diborane(4) towards pyridine and isocyanide: formation of boraalkene–pyridine complex and ortho- functionalized pyridine derivatives. *Chem. Sci*. **9**, 1301-1310 (2018)).

Given that many organic mixed-valence systems with three stable redox states have fueled a variety of fields, the manuscript will be of high interest to the organic, inorganic and material communities. In my opinion, this study is suited for an article in Nat. Comm. provided that the following points are addressed:
1. The experimental work is well done, but the theoretical support is modest. The radical cation will be resistant towards disproportionation if the oxidative formation of the radical cation from the neutral state is more favorable than further oxidation to the dication. The separation of the two redox waves in experimental CVs is related to chemical potential change (delta μ), which is easily calculated by frontier molecular orbital energies. How does the BN/CC isosterism effect the FMO energies and thus widen the redox range? Can the authors provide rationale?

Response: Indeed, the chemical potential change can be calculated using the method of Hansmann and Munz (*Chem. Sci.* **11**, 4138-4149 (2020)), using the FMO energies for the dication (E_{LUMO} = -8.38 eV) and for the radical cation (E_{HOMO} = -6.84 eV, $E_{LUMO}(\beta)$ = -4.92 eV), yields the following chemical potentials

μ_2 (reduction of dication) = 8.38 eV
μ_1 (reduction of radical cation) = (6.84 + 4.92)/2 eV = 5.88 eV

Clearly reduction of the dication is much more favourable than reduction of the monocation to the neutral species - in line with the experimental observation of an extremely wide redox range for the intermediate radical cation state.
The role of formal BN/CC 'substitution' in influencing the FMOs in these systems, and as a consequence the redox stability range associated with the radical cation intermediate oxidation state, can perhaps most readily be understood by reference to the LUMOs of the three dicationic species sketched out in the ChemDraw diagram below. These refer to the dications derived from the bis(NHC) system (originally synthesized by Ghadwal and co-workers - *Angew. Chem. Int. Ed.* **57**, 5838−5842 (2018)), our mixed BN/CC system (i.e. **1**$^{2+}$) and the (hypothetical) bis(BN) system (shown top-to-bottom in the diagram). As outlined above, the analysis of Hansmann and Munz suggests that the energy of the LUMO of this dication is important in estimating the reduction potential associated with the dication; this in turn is key to the redox stability of the intermediate radical cation (which is governed by the relative potentials associated with the reduction processes of the mono- and dications).
Experimentally it is observed that (i) the all carbon system has a narrow redox range associated with the radical cation, (ii) our mixed BN/CC system has a much wider redox range associated with the radical cation; and (iii) for the bis(BN) system, the dication cannot be isolated (too oxidizing). These observations suggest that the LUMO of the dicationic species becomes increasingly more stabilized with increasing BN for CC substitution. As such, the bis(BN) dication is very easily reduced (indeed the dication is so oxidizing that no examples have yet been isolated - see Kinjo *et al.*, *Chem. Sci.* **8**, 7419-7423 (2017) for a related example), our mixed BN/CC system is still easily reduced (but the dication in this case is at least isolable), and the dication of the CC/CC system is the least readily reduced (to the extent that the reduction potential of the dication and mono-cation are relatively more similar, and the intermediate radical is only stable over a narrow redox range).
The underlying reason for the stabilization of the LUMO of the dication with increasing BN incorporation can be understood by reference to the changing degree of conjugation within the three LUMO orbitals. This is most readily illustrated by reference to the DFT-calculated LUMO of **1**$^{2+}$ (also reproduced below). Here it is clear that, while conjugation extends readily from the central ring across the exocyclic C-C bond, the same is not the case with the corresponding B-N exocyclic linkage (presumably due to poorer energy match between formal boryl/pyridine fragments compared to their all-carbon analogues). Similar situations pertain for the LUMOs of the CC/CC and BN/BN systems - i.e. ready conjugation through exocyclic CC, but to a much lesser extent through BN linkages. Reduced conjugation is also associated with increasing localization of positive charge in the central ring (and to a reduced extent on pendant imidazolium heterocycles) as CC for BN substitution increases, and consequently with successive stabilization of the LUMO (which becomes more localized in the more positively charged central ring).

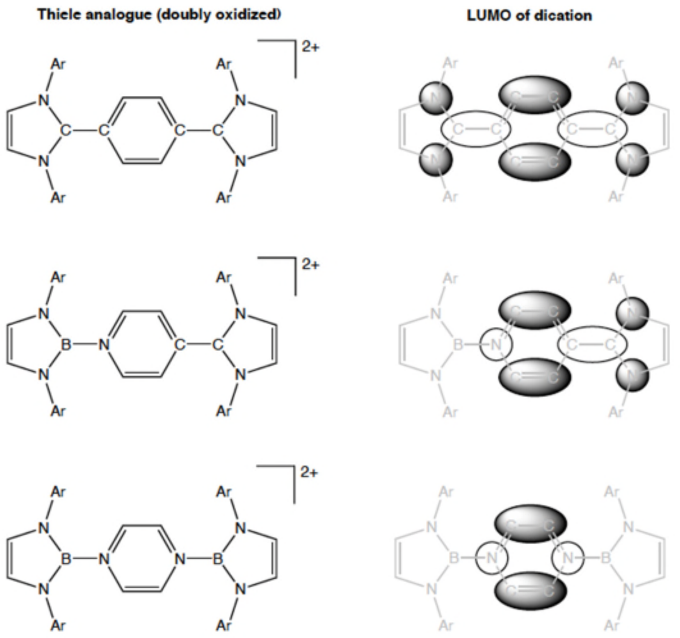

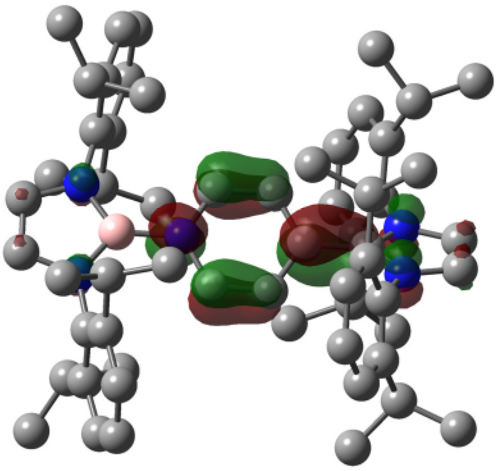

With this in mind, we have added in an additional section to the conclusions paragraph:

'We propose that formal BN/CC substitution is critical in lowering the energy of the LUMO of the dication $\mathbf{1}^{2+}$ (see Supporting Information, Figure S19) compared to its all-carbon analogue, making $\mathbf{1}^{2\bullet+}$ more oxidizing, and rendering the intermediate radical cation $\mathbf{1}^{\bullet+}$ less prone to oxidation than its neutral counterpart **1**.[13] This hypothesis accounts for the stability of $\mathbf{1}^{\bullet+}$ over an unprecedented redox range, and is consistent with the experimental observation that dicationic species derived from doubly BN-substituted systems (Scheme 1) are too oxidizing to be experimentally accessible.'

2. As shown in Scheme 2, species 1 and 2 have biradical character (open shell singlet or triplet). Can the authors calculate it? How does it compare with their bis-NHC analogs?
Response: Neutral **1** doesn't show any significant diradicaloid character computationally. The singlet-triplet gap is relatively high at 1.76 eV, 169.4 kJ mol^{-1}.

3. Are there any experimental evidences for the formation of a FLP consisting of {(HCDippN)2B}OTf and IDipp? As NHCs and CAACs are known to react with pyridinium salts, {(HCDippN)2B}OTf may form a salt with pyridine followed by para-attack by NHC.
Response: The combination of {$(HCDippN)_2B$}OTf and IDipp can be shown by multinuclear NMR measurements to constitute an FLP (NMR of mixture is simply the superposition of the spectra of the individual components). In terms of the reaction with pyridine, this is very likely to proceed via coordination at boron followed by para nucleophilic attack by the carbene (indeed the stepwise reaction of the borane with pyridine can be shown to give an adduct, which then reacts with sequentially added carbene to generate the same intermediate).

4. 13C NMR data are not given for (HCDippN)2BOTf.
5. No EA are given for (HCDippN)2BOTf and 1.

Response: these are now included in the ESI for (HCDippN)2BOTf. As noted in the ESI for compound **1** - crystalline samples are impossible to obtain in our hands - hence the synthesis of **2** as a 'surrogate.' Since **2** does crystallize, we could then obtain elemental microanalysis for **2**.

6. Please indicate the contribution of each transition from TD-DFT calculations.
Response: The data shown below has been added into the revised supporting information (Table s1)

352 nm 0.0452
232 -> 242 0.44348 contribution: 39.3%
232 -> 243 -0.40356 contribution: 32.6%
232 -> 244 0.36003 contribution: 25.9%

515 nm 0.0315
232 -> 234 0.14514 contribution: 4.2%
232 -> 235 0.66635 contribution: 88.8%
232 -> 236 0.13973 contribution: 3.9%

413 nm 0.143
232A -> 234A 0.59616 contribution: 35.5%
232A -> 235A 0.17130 contribution: 2.9%
232A -> 238A 0.70345 contribution: 49.5%
224B -> 232B -0.25282 contribution: 6.4%
231B -> 232B -0.10225 contribution: 10.5%

518 nm 0.0858
232A -> 238A 0.11270 contribution: 1.3%
231B -> 232B 0.98146 contribution: 96.3%

381 nm 0.0054
223 -> 232 0.69840 contribution: 97.6%

580 nm 0.257
231 -> 232 0.70179 contribution: 98.5%

REVIEWER COMMENTS

Reviewer #1 (Remarks to the Author):

The authors have substantially and satisfactory revised the manuscript according to the reviewer's comments. Thus I would like to recommend this manuscript for publication in Nature Communications.

I have only minor point regarding the TOC: CV Figure is overlapping with Scheme in my PDF file.

Reviewer #2 (Remarks to the Author):

This paper was adequately revised according to the first round of reviewing. This reviewer just suggests to include the explanation of the negative spin density on the boron nucleus into the main text before the final acceptance.

Reviewer #4 (Remarks to the Author):

In my opinion, the authors have addressed all the concerns by reviewers and uploaded a suitable revised MS. I therefore support the publication of the present form of MS without further changes.

Responses to reviewers' comments

Reviewer #1 (Remarks to the Author):

The authors have substantially and satisfactory revised the manuscript according to the reviewer's comments. Thus I would like to recommend this manuscript for publication in Nature Communications.

I have only minor point regarding the TOC: CV Figure is overlapping with Scheme in my PDF file.

Response: As requested by the editorial team the ToC figure has been removed

Reviewer #2 (Remarks to the Author):

This paper was adequately revised according to the first round of reviewing. This reviewer just suggests to include the explanation of the negative spin density on the boron nucleus into the main text before the final acceptance.

Response: This is now included in the caption to Figure 5 ('EPR Studies')

Reviewer #4 (Remarks to the Author):

In my opinion, the authors have addressed all the concerns by reviewers and uploaded a suitable revised MS. I therefore support the publication of the present form of MS without further changes.
